# The global distribution of *Banana bunchy top virus* reveals little evidence for frequent recent, human-mediated long distance dispersal events

**DOI:** 10.1093/ve/vev009

**Published:** 2015-09-10

**Authors:** Daisy Stainton, Darren P. Martin, Brejnev M. Muhire, Samiuela Lolohea, Mana’ia Halafihi, Pascale Lepoint, Guy Blomme, Kathleen S. Crew, Murray Sharman, Simona Kraberger, Anisha Dayaram, Matthew Walters, David A. Collings, Batsirai Mabvakure, Philippe Lemey, Gordon W. Harkins, John E. Thomas, Arvind Varsani

**Affiliations:** ^1^School of Biological Sciences and Biomolecular Interaction Centre, University of Canterbury, Private Bag 4800, Christchurch, 8140, New Zealand; ^2^Department of Clinical Laboratory Sciences, University of Cape Town, Cape Town, South Africa; ^3^Tonga College, Tongatapu, Kingdom of Tonga; ^4^Ministry of Agriculture and Food, Forests and Fisheries, Kingdom of Tonga; ^5^Bioversity International, PO Box 18937, Bujumbura, Burundi; ^6^Bioversity International Uganda Office, Naguru, Kampala, Uganda; ^7^Queensland Department of Agriculture, Fisheries and Forestry, Ecosciences Precinct, GPO Box 267, Brisbane, QLD 4001, Australia; ^8^South African National Bioinformatics Institute, MRC Unit for Bioinformatics Capacity Development, University of the Western Cape, Bellville, 7535, South Africa; ^9^KU Leuven, University of Leuven, Department of Microbiology and Immunology, Rega Institute for Medical Research, Clinical and Epidemiological Virology, Minderbroedersstraat 10, B-3000 Leuven, Belgium; ^10^The University of Queensland, Centre for Plant Science, Queensland Alliance for Agriculture and Food Innovation, Ecosciences Precinct, PO Box 46, Brisbane, QLD, 4001, Australia; ^1^^1^Department of Plant Pathology and Emerging Pathogens Institute, University of Florida, Gainesville, FL 32611, USA

**Keywords:** phylogeography, *Banana bunchy top virus*, Nanoviridae, babuvirus, recombination, reassortment.

## Abstract

*Banana bunchy top virus* (BBTV; family Nanoviridae, genus *Babuvirus*) is a multi-component single-stranded DNA virus, which infects banana plants in many regions of the world, often resulting in large-scale crop losses. We analyzed 171 banana leaf samples from fourteen countries and recovered, cloned, and sequenced 855 complete BBTV components including ninety-four full genomes. Importantly, full genomes were determined from eight countries, where previously no full genomes were available (Samoa, Burundi, Republic of Congo, Democratic Republic of Congo, Egypt, Indonesia, the Philippines, and the USA [HI]). Accounting for recombination and genome component reassortment, we examined the geographic structuring of global BBTV populations to reveal that BBTV likely originated in Southeast Asia, that the current global hotspots of BBTV diversity are Southeast Asia/Far East and India, and that BBTV populations circulating elsewhere in the world have all potentially originated from infrequent introductions. Most importantly, we find that rather than the current global BBTV distribution being due to increases in human-mediated movements of bananas over the past few decades, it is more consistent with a pattern of infrequent introductions of the virus to different parts of the world over the past 1,000 years.

## 1 Introduction

Bananas are grown in over 130 countries and are ranked fourth, after wheat, rice, and maize, in importance as a food crop in the world (Perrier et al. 2011; http://www.fao.org/docrep/i3627e/i3627e/i3627e.pdf). Domesticated bananas are thought to have originated somewhere in the vicinity of New Guinea, Indonesia, the Philippines, or the Southeast Asia Peninsula (Perrier et al. 2011) between 7,000 and 10,000 years ago (Denham et al. 2003). Banana cultivation subsequently spread to other parts of the world reaching Cameroon in West Africa and the Indian Ocean island of Madagascar possibly as early as 3,000 years ago. During the period between 1,500 and 700 years ago, different banana varieties were likely introduced and reintroduced to Africa and the south-west Indian Ocean Islands many times (Lejju, Robertshaw, and Taylor 2006; Randrianja and Ellis 2009).

Banana bunchy top disease (BBTD) is one of the most important diseases of banana, causing severe crop losses in many banana-growing regions outside the Americas (Dale 1987; Rybicki and Pietersen 1999; Rybicki 2015). Banana plants apparently displaying BBTD symptoms were described in Fiji as early as the 1880s (Magee 1927). In the 1930s, the banana aphid, *Pentalonia nigronervosa*, was found to transmit the disease in a persistent manner (Magee 1940). However, it was not until the 1990s that an icosahedral single-stranded DNA virus with six genome components was identified as the causative agent. This virus, *Banana bunchy top virus* (BBTV) (Harding, Burns, and Dale 1991; Thomas and Dietzgen 1991; Harding et al. 1993; Burns, Harding, and Dale 1994, 1995), is now recognized as the type member of the genus *Babuvirus* in the family Nanoviridae.

The six genome components of BBTV are each approximately 1,000 nt long and are called DNA-R, DNA-U3, DNA-S, DNA-M, DNA-C, and DNA-N (formerly DNA-1 to DNA-6, respectively) (King et al. 2012). DNA-R encodes a replication-associated protein (*rep*), DNA-S a capsid protein (*cp*), DNA-M a movement protein (*mp*), DNA-C a cell-cycle link protein (*Clink*), and DNA-N a nuclear shuttle protein (*nsp*) genes (Hafner et al. 1997b; Aronson et al. 2000; Wanitchakorn, Harding, and Dale 2000; Wanitchakorn et al. 2000). The function of DNA-U3 is currently unknown. All components of individual viruses contain two sequence elements which are highly similar across the components: a common region stem-loop (CR-SL) element and a common region major (CR-M) element (Burns, Harding, and Dale 1995). The CR-SL is involved in replication and contains both a hairpin structure with a highly conserved non-anucleotide sequence (TATTATTAC) and three repeated five nucleotide long sequences, called iterons, that are likely involved in the recognition and/or binding of Rep to the virion strand origin of replication (*v-ori*) (Burns, Harding, and Dale 1995; Herrera-Valencia et al. 2006). The CR-M is thought to be involved in transcription (Burns Harding, and Dale 1995) and also contains most of the binding sites for a primer molecule that is involved in complementary strand DNA synthesis (Hafner, Harding, and Dale 1997).

Components of BBTV isolates broadly fall into two geographically well-defined phylogenetic groups, the South Pacific group (SPG) and the Asian group (AG) (Karan, Harding, and Dale 1994). Despite these phylogenetic groups having been defined based on the geographic origins of genomic component sequences available in the mid-1990s, subsequently determined BBTV sequences have continued to phylogenetically cluster within one or the other of these groups, with almost all sequences sampled outside of Southeast Asia (SEA) falling into the SPG. Although the SPG and AG have also been, respectively, referred to as the Pacific/Indian Ocean and the SEA groups (Yu et al. 2012), here we will continue to use their original names.

It is likely that this geographic structuring has arisen because the rates of natural and/or human-mediated long-distance BBTV movement have been low enough for geographically separated populations of these viruses to have differentiated from one another. It remains unknown, however, whether the current geographical distribution of BBTV variants arose (1) concomitantly with the slow, pre-historic spread of banana cultivation across the Pacific, the Indian Ocean, Asia, and Africa, (2) during the pre-globalization ebb and flow of banana varieties across the Pacific and Indian Oceans between 100 and 1,500 years ago, or (3) during the modern globalization era as a consequence of poorly regulated agricultural trade. It is additionally plausible that the current distribution of BBTV might have arisen during this entire span of time. Importantly, the degrees of geographic structure evident within contemporary genomic sequence data might be high enough to yield insights into when and from where the BBTV populations in particular continents, countries, or territories were founded. Such insights would be extremely valuable in determining, for example, whether modern movements of banana germplasm across the globe have had an appreciable impact on BBTV distributions.

The potential for human-mediated dissemination of BBTV is high since cultivated bananas are sterile and are propagated vegetatively. Also, a banana plant infected with BBTV can take between 25 and 85 days to develop visible symptoms (Hooks et al. 2008) meaning that infected but symptomless banana propagules could be inadvertently transferred to regions where *P. nigronervosa* is present. The BBTV variants within infected propagules might then be successfully transmitted and establish new BBTV populations within bananas or wild hosts.

It is also likely that, as is the case with other related single-stranded DNA viruses (Duffy and Holmes 2008; Duffy, Shackelton, and Holmes 2008; van der Walt et al. 2008; Firth et al. 2009; Harkins et al. 2009, 2014; Grigoras et al. 2010; Kraberger et al. 2013), BBTV is evolving at a sufficient rate that evidence of such movement events should be encoded within the phylogenetic relationships of genomic component sequences sampled from extant BBTV populations.

Phylogenetic inference of BBTV movement dynamics might, however, be confounded by two other evolutionary processes that occur in BBTV, genome component reassortment, and homologous recombination. Because of genome components each being packaged individually into separate virions, new infections that are propagated from mixed BBTV infections will frequently contain an assortment of different genome components. BBTV isolates that have genome components derived from two or more different parental viruses have been inferred using a variety of phylogenetic (Hu et al. 2007; Yu et al. 2012) and statistical recombination detection methods (Martin et al. 2010; Stainton et al. 2012). Similar examples of component reassortment have also been found in a number of other nanovirus species (Grigoras et al. 2014; Savory and Ramakrishnan 2014).

The known sequences of many individual BBTV genome components also carry evidence of homologous recombination (Hyder et al. 2011; Stainton et al. 2012; Wang et al. 2013; Banerjee et al. 2014). Although the accuracy of phylogenetic reconstructions for individual genome components could be significantly undermined by homologous recombination, both recombination and reassortment will undermine the accuracy of full-genome phylogenetic reconstructions (Schierup and Hein 2000; Posada and Crandall 2002).

**Figure 1.**
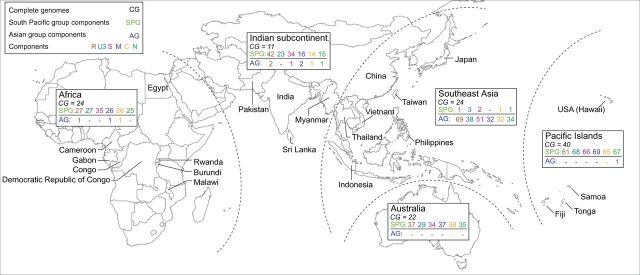
Geographical distribution of BBTV isolates. Summaries of component numbers and full genomes are provided for different regions. Accession numbers and specific component information can be found in Fig. 2 and Supplementary Table S1.

Both to gain a more detailed view of global BBTV diversity and to assess the geographical structuring of BBTV populations at higher resolution than has previously been achievable, we determined the sequences of 855 full BBTV genome components from samples collected from across much of the known BBTV geographic range (Fig. 1, Supplementary Table S1). Accounting for recombination, reassortment, and inferred rates of BBTV evolution, we find that the diversity and phylogeographic structure of contemporary-known BBTV populations is entirely consistent with there having been infrequent introductions of the virus to different parts of the world over the past 1,000 years.

## 2 Materials and methods

### 2.1 Extraction and sequencing

**Figure 2.**
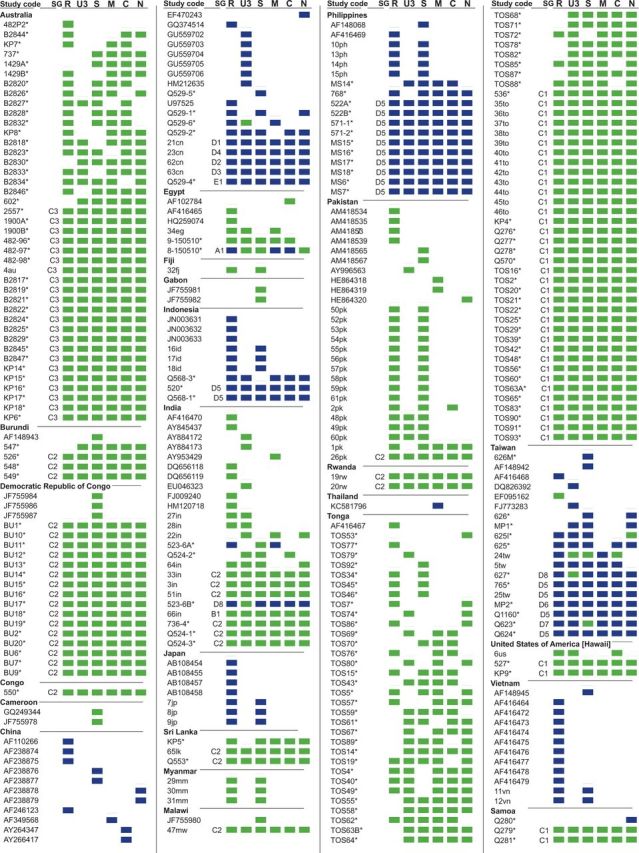
Overview of sequenced BBTV components. Isolates are grouped by country, with each individual component depicted with a blue or green square depending on whether the component falls phylogenetically into the AG (blue) or SPG (green) group (phylogenetic tree not shown). Isolates with asterisks were sequenced as part of this study. Full-genome subgroups (SG), based on percentage pairwise identities, are shown for all isolates with all six components sequenced. Full isolate information, including accession numbers and references, are in Supplementary Table S1.

Samples were collected from 171 banana plants displaying stunting, bunched-up leaves, and Morse-code like streaking between leaf margins and the midrib: all of which are symptoms characteristic of BBTD. Samples were collected between 1989 and 2012 from Australia (*n* = 40 isolates), four African countries (*n* = 23 isolates), three Pacific island groups (*n* = 69 isolates), two Indian Subcontinent countries (*n* = 8 isolates), and four SEA/Far East countries (*n* = 31 isolates), summarized in Fig. 2 and Supplementary Table S1.

DNA extractions, amplification, and sequencing of BBTV genome components were carried out as described previously (Stainton et al. 2012). Briefly, sampled leaf material (fresh or dried) was homogenized, and total DNA was extracted using an Epoch plant purification kit (Epoch Life Science Inc., USA). Circular DNA was preferentially enriched using the TempliPhi amplification kit (GE Healthcare, USA) as described previously (Owor et al. 2007; Shepherd et al. 2008). BBTV genome components were polymerase chain reaction amplified using component-specific back-to-back primers described in Stainton et al*.* (2012). The resulting amplicons were resolved on an agarose gel, gel purified, cloned and single transformed plasmid clones were sequenced at Macrogen Inc. (South Korea). Sequence contigs were assembled using DNA Baser Sequence Assembler v4 (Heracle Biosoft SRL, Romania). Where possible, all six components were sequenced from each sample, although for some samples we were unable to recover all of the components (Fig. 2, Supplementary Table S1). Sequences from this study have been deposited in GenBank (accession numbers KM607005 - KM607859).

### 2.2 Datasets

All full components sequenced as part of this study, along with all full BBTV component sequences available in GenBank (downloaded 1st March 2014, see Supplementary Table S1 for isolate information) were split into individual component-specific datasets (CSD) all starting at the ‘TATTAC’ region of the nonanucleotide sequence motif. These sequences were aligned using MUSCLE (Edgar 2004) implemented in MEGA5 (Edgar 2004; Tamura et al. 2011). Aligned component sequences identified from the same sample were then concatenated into a single sequence. As outlined in Stainton et al*.* (2012), blank sequences (i.e., composed entirely of ‘-’ characters) were used where component sequences were not available both to maintain the component order and for alignment purposes. These concatenated sequences were labeled as the concatenated dataset (CD) (Supplementary Table S1 contains information on cognate BBTV components). From this dataset, a new BBTV genome dataset called the full-genome dataset (FGD) was created containing all available full-genome sequences (all six components sequenced). *Abaca bunchy top virus* (ABTV) sequences were used as an out group for all datasets except for the generation of the maximum clade credibility (MCC) tree. Following recombination and reassortment analysis, a recombination-free CSD (RF-CSD) for each component and a recombination and reassortment-free FGD (RF-FGD) were constructed from the CSDs and FGD, respectively (see below for recombination and reassortment details).

### 2.3 Pairwise nucleotide sequence identity analyses

Percentage pairwise nucleotide identities of complete BBTV genomes (in the context of the FGD) and of individual components (in the context of the CSDs) were determined using Sequence Demarcation Tool (SDT) v1.2 (Muhire, Varsani, and Martin 2014) with the MUSCLE-based alignment option. CSDs and the FGD were all analyzed with SDT without accounting for recombination. Distributions of pairwise nucleotide identities of the FGD and CSDs were used to tentatively classify BBTV genomes into groups and subgroups based on the majority of the components in a similar way to Varsani et al*.* (2014).

All CSD were split into AG and SPG based on neighbor-joining trees reconstructed using the Jukes–Cantor model as implemented in MEGA 5 (Tamura et al. 2011) (data not shown), and percentage pairwise identity was calculated for each group using SDT v1.2.

Percentage pairwise identities were determined for the FGD and CSD sequences for five geographic regions: Africa (Burundi, Cameroon, Congo, Democratic Republic of Congo, Egypt, Gabon, Malawi, and Rwanda), the Indian Subcontinent (India, Myanmar, Pakistan, and Sri Lanka), SEA/Far East Asia (China, Indonesia, Japan, Philippines, Taiwan, Thailand, and Vietnam), Pacific Islands (Fiji, Hawaii, Kingdom of Tonga, and Samoa), and Australia.

### 2.4 Recombination and reassortment analyses

All recombination and reassortment events were detected using RDP4.27 (Martin et al. 2010), a recombination detection program which implements the following detection methods: RDP (Martin and Rybicki 2000), GENECONV (Padidam, Sawyer, and Fauquet 1999), Bootscan (Martin et al. 2005), Maxchi (Smith 1992) Chimera (Posada and Crandall 2001), SiScan (Gibbs, Armstrong, and Gibbs 2000), and 3Seq (Boni, Posada, and Feldman 2007). Recombination events were considered credible when an event was identified by at least three detection methods with an associated *P* value < 0.05 and with at least one method having an associated *P* value < 0.001 coupled with supporting phylogenetic evidence. Reassortment events were considered credible when, along with phylogenetic evidence, an event was identified by at least two detection methods with an associated *P* value < 0.05, with at least one method having an associated *P* value < 0.001. Intra-component recombination events were identified using the single CSDs. Reassortment events were identified using the CD. Specifically, recombination events identified by RDP4.27 that had associated breakpoints which spanned an entire component were identified as reassortment events.

Because of the large number of sequences being analyzed, as well as issues with accurately aligning all six components, a dataset containing all sequences from all components was not used to detect evidence of possible inter-component recombination. Therefore, all intra-component recombinant regions with an unknown minor parent were further analyzed using BLASTn (Altschul et al. 1990) to determine whether transferred sequence fragments identified as having unknown origins could have credibly been derived from different BBTV components.

### 2.5 Phylogenetic analysis of BBTV geographic distributions

Maximum likelihood (ML) phylogenetic trees were constructed using the recombination-free CSDs with PHYML 3 (Guindon et al. 2010) applying the best fit nucleotide substitution model for each dataset determined using jModelTest (Posada 2008) with 100 bootstrap replicates to determine branch support. For the recombination and reassortment-free FGD, an ML tree was constructed using RAxML (Stamatakis 2014) with 100 bootstrap replicates. All phylogenetic trees were rooted with ABTV sequences, and branches with <60 per cent bootstrap support were collapsed using Mesquite v2.75 (http://mesquiteproject.org/). RAxML was used for these particular trees rather than PHYML because it has been specifically optimized to construct phylogenetic trees from sequences containing large amounts of missing data (Izquierdo-Carrasco, Smith, and Stamatakis 2011).

To assess the time scales over which BBTV movements have likely occurred and identify the locations of the ancestral sequences involved, we analyzed the recombination and reassortment-free CD dataset (RF-CD; *n* = 224 dated sequences) under a constant population size and strict-clock discrete diffusion phylogeographical model with stochastic search variable selection (BSSVS) (Lemey et al. 2009) implemented in BEAST v1.8.1 (Drummond and Rambaut 2007). Seven geographical locations were considered: Africa (Burundi, Cameroon, Congo, and Democratic Republic of Congo), the Indian Subcontinent (India, Pakistan, and Sri Lanka), SEA (China, Indonesia, Philippines, Taiwan, and Thailand), Australia, Hawaii, Samoa, and Tonga. The Pacific Islands were classified as independent locations as we had a specific interest in determining whether statistically supported movements between the different Pacific island states and the rest of the world could be reliably inferred.

Bayes factor (BF) tests were used to determine the approximate statistical support for the inferred BBTV dispersal pathways, where a BF of less than five is not well supported, a BF of more than five implies substantial support, and BFs of between 10 and 100 are indicative of strong support (Kass and Raftery 1995; Lemey et al. 2009). Ten replicate runs of the Markov chain were run until the effective sample sizes for all of the model parameters were more than 200 and checked for convergence using TRACER v1.6 (http://tree.bio.ed.ac.uk/software/tracer/).

To determine whether sampling bias due to uneven sampling sizes from the different locations considered has systematically influenced our parameter estimates, the BEAST analyses were also carried out with the location states of the sequences randomized and the location state probabilities of the root node compared with those determined for the datasets analyzed without randomization. SPREAD (Bielejec et al. 2011) was used to produce a graphical animation in keyhole markup language (kml) format illustrating the historical spatio-temporal movement dynamics of BBTV that can be viewed in Google Earth.

## 3 Results and discussion

### 3.1 Sample collection and sequencing

Although BBTV populations seriously constrain banana production throughout much of the eastern hemisphere, the worldwide genetic diversity of BBTV remains poorly understood. We therefore amplified, cloned, and sequenced 855 complete BBTV components (DNA-R, *n* = 137; DNA-U3, *n* = 138; DNA-S, *n* = 146; DNA-M, *n* = 146; DNA-C, *n* = 143; DNA-N, *n* = 145) from 171 BBTV infected banana pants from fourteen countries spanning the known geographical range of this virus (Fig. 2, Supplementary Table S1, accession numbers KM607005–KM607859).

A subset of the newly determined genome component sequences constitute 94 complete BBTV genomes (i.e., instances where all six components have been sequenced from a single sample). These 94 genomes include those sampled in countries/territories from which either no BBTV sequence data were previously available (Congo and Samoa) or for which no full genomes have previously been sequenced (Burundi, Democratic Republic of Congo, Egypt, Indonesia, the Philippines, and Hawaii). This new sequence data more than doubles the number of publically available BBTV full-genome component sequences. All GenBank accession numbers for these and other publically available BBTV sequences used in this study can be found in Supplementary Table S1.

In total, 1,191 BBTV and 13 ABTV component sequences (ABTV DNA-M *n* = 3, all other components *n* = 2) were assembled into seven datasets: CD (*n* = 317), CSD DNA-R (*n* = 242), CSD DNA-U3 (*n* = 190), CSD DNA-S (*n* = 225), CSD DNA-M (*n* = 186), CSD DNA-C (*n* = 180), and CSD DNA-N (*n* = 181). These sequences have collectively been recovered from a total of 317 plant samples (170 in this study) from twenty-five countries (fourteen sampled in this study; Fig. 2 and Supplementary Table S1). An FGD containing isolates with all six component sequences was assembled from the CD and contained 121 full BBTV genomes and two full ABTV genomes.

### 3.2 Classification of the genome segments and full genomes

The DNA-U3 components were most diverse, sharing >74 per cent pairwise identity followed by DNA-S and DNA-M (both sharing >82% pairwise identity), and the DNA-N, DNA-C, and DNA-R components that shared >83 per cent, >85 per cent, and >88 per cent pairwise identity, respectively. Collectively, the segments in the FGD shared >85 per cent pairwise identity. For the FGD, the genome sequences which shared >85 per cent but <94 per cent pairwise identity were subdivided into groups A–E. Within these groups, genomes with >98 per cent pairwise identity were further divided into subgroups A1, B1, C1–3, D1–8, and E1 (Fig. 2; Supplementary Table S1).

With the exception of DNA-S, the genetic diversity among the currently sampled AG genome components is generally greater than that among the corresponding SPG components: DNA-R (AG > 91%, SPG > 94%), DNA-U3 (AG > 76%, SPG > 81%), DNA-M (AG > 89%, SPG > 91%), DNA-C (AG > 89%, SPG > 94%), and DNA-N (AG > 89%, SPG > 91%) components. In the case of DNA-S, the AG sequences are >92 per cent identical, whereas the SPG sequences are >87 per cent identical.

The percentage pairwise identities of genome components sampled from five major regions of the world (Africa, the Indian subcontinent, SEA/Far East, the Pacific Islands, and Australia) indicated that the greatest degree of BBTV sequence diversity occurs within the SEA/Far East/Indian subcontinent regions (Table 1). This is true for the FGD and all individual components. The significant diversity observed in Africa is contributed mainly by the AG-like DNA-R, -M, and -C components of the Egyptian isolate, 8–150,510 (Fig. 2). This suggests that the true global diversity of BBTV could be best inferred by increased sampling effort in these regions. DNA-M diversity is highest within the Indian subcontinent, whereas for DNA-U3, the diversity is highest in SEA (Table 1).

**Table 1.** Percentage pairwise identities of individual BBTV genome components that have been sampled from different geographical regions.

Full genome/component	Region	Pairwise identity (%)	Number of pairwise comparisons	Average pairwise identity (%)	Standard deviation (%)
Full genome	Africa	>91.3	276	98.2	2.0
	Australia	>97.3	231	99.0	0.5
	Indian subcontinent	>87.1	66	95.2	3.4
	Pacific islands	>97.3	780	97.9	0.7
	SEA	>90.1	276	96.5	2.6
DNA-R	Africa	>90.1	378	98.2	2.2
	Australia	>98.6	666	99.5	0.3
	Indian subcontinent	>89.5	946	97.9	2.6
	Pacific islands	>95.5	1,830	98.7	0.9
	SEA	>89.1	2,415	96.8	2.2
DNA-U3	Africa	>87.2	351	96.3	3.4
	Australia	>96.7	406	98.5	0.8
	Indian subcontinent	>82.5	253	93.1	4.1
	Pacific islands	>91.3	2,278	91.3	1.8
	SEA	>75.9	820	90.8	6.4
DNA-S	Africa	>96.8	595	98.6	0.6
	Australia	>88.9	561	98.5	2.0
	Indian subcontinent	>87.8	595	97.2	2.4
	Pacific islands	>96.9	2,145	98.6	0.6
	SEA	>88.2	1,378	96.7	2.9
DNA-M	Africa	>82.1	351	97.3	4.2
	Australia	>92.4	666	97.8	2.2
	Indian subcontinent	>83.4	183	94.6	5.7
	Pacific islands	>91.8	2,346	96.6	2.1
	SEA	>89.2	496	95.9	3.3
DNA-C	Africa	>86.7	351	97.8	3.0
	Australia	>97.6	703	99.0	0.4
	Indian subcontinent	>86.4	105	96.3	3.4
	Pacific islands	>97.5	2,080	98.7	0.4
	SEA	>86.3	528	95.9	3.5
DNA-N	Africa	>97.2	300	98.9	0.5
	Australia	>98.9	595	99.5	0.2
	Indian subcontinent	>85.0	120	96.3	4.4
	Pacific islands	>83.8	2,278	98.0	2.4
	SEA	>84.7	595	95.9	4.1

### 3.3 Reassortment analyses

Given that BBTV genome components are individually encapsidated, mixed infections will often result in genome component reassortment (Hu et al. 2007; Stainton et al. 2012). To ensure the accuracy of our FGD phylogenetic analyses, it was vital that we identified and removed from our datasets genome components that had been acquired by reassortment. Towards this end, the CD was analyzed for evidence of reassortment using RDPv4.27 (Martin et al. 2010), with manual identification of reassortment events as detected recombination events that had inferred breakpoint locations spanning entire components (Stainton et al. 2012). Given that this analysis involved almost four times more full genomes than previous BBTV reassortment analyses, it is not surprising that of the seventy-five isolates detected as reassortants, only 10 had been detected previously (Hu et al. 2007; Stainton et al. 2012).

**Figure 3.**
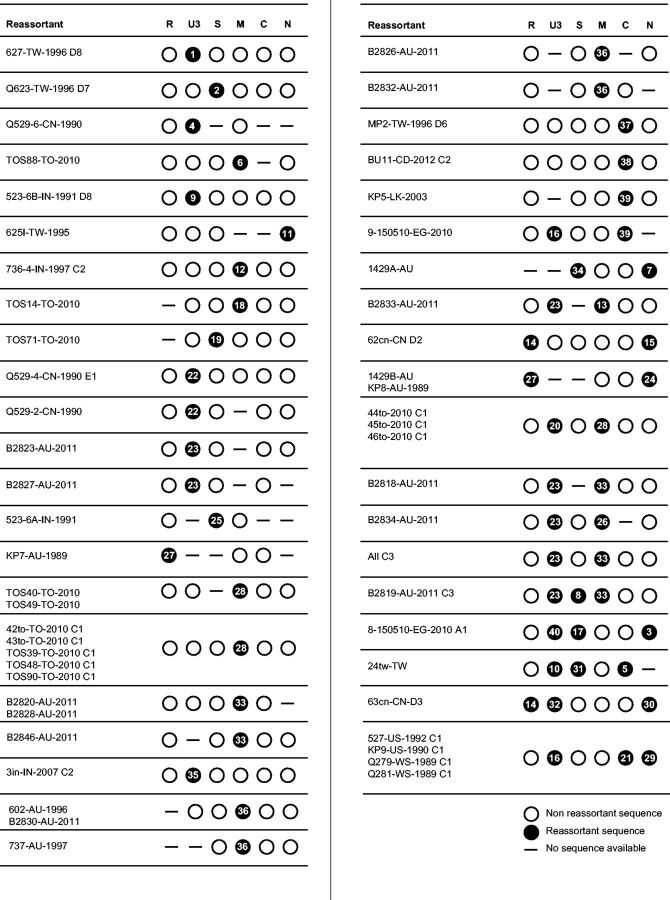
Detected reassortment events. As not all reassortant isolates consist of full genomes, circles depict component sequences, which are available and a dash indicates where no component sequence is available. Components are shown as either non-reassortant sequences (white filled circles) or as reassortant sequences (black circles) with the corresponding reassortant event number. Further information on reassortment events can be found in Supplementary Table S2.

These seventy-five isolates carried evidence of forty different reassortment events (Fig. 3, Supplementary Table S2). All components were represented among these events, albeit with some components having been transferred more than others. Component DNA-U3 was found to be the most commonly transferred component (eleven events), followed by DNA-M (eight events), DNA-S and DNA-N (both with seven events), DNA-C (five events), and DNA-R (two events).

Similar reassortment analyses in *Cardamom bushy dwarf virus* (CBDV) and viruses in the genus Nanovirus that lack a DNA-U3 component have also found that DNA-M and/or DNA-N are among the most frequently transferred nanovirus components during reassortment (Grigoras et al. 2014; Savory and Ramakrishnan 2014). However, DNA-U3, which is only present in Babuviruses (BBTV, ABTV, and CBDV), was not found to be among the most frequently transferred genome components during CBDV reassortment (Savory and Ramakrishnan 2014), suggesting that patterns of component transfer are not absolutely conserved between different species.

Of the seventy-five reassortant genomes that we detected, thirty-four had one detectable reassortment event, thirty-three had two, and eight had three. Overall, ∼38 per cent of all isolates with at least three sequenced components (75/196) show evidence of at least one component having been acquired by reassortment. Crucially, twelve of the forty reassortment events were each detected in multiple genomes. This strongly suggests that these events occurred in an ancestor of these genomes and therefore that reassortment yielded viable viruses that went on to become epidemiologically relevant.

Our detection of reassortment events between AG and SPG genomes sampled in Egypt, China, India, and Taiwan (Fig. 2) is consistent with the geographic range of the AG and SPG lineages overlapping in these regions. This overlap suggests that the Indian/Southeast Asian/Far Eastern region is likely the geographic hotspot of BBTV diversity and might even be the region where the most recent common ancestor of all currently sampled BBTV isolates originated.

### 3.4 Recombination analyses

A number of studies have identified potential recombination events in BBTV (Fu et al. 2009; Islam et al. 2010; Hyder et al. 2011; Stainton et al. 2012; Banerjee et al. 2014), and, as with reassortment, it was important to account for these events during our subsequent phylogenetic analyses. We analyzed the CSDs to identify recombinant sequences, the locations of recombination breakpoints, and the identities of likely parental viruses.

**Figure 4.**
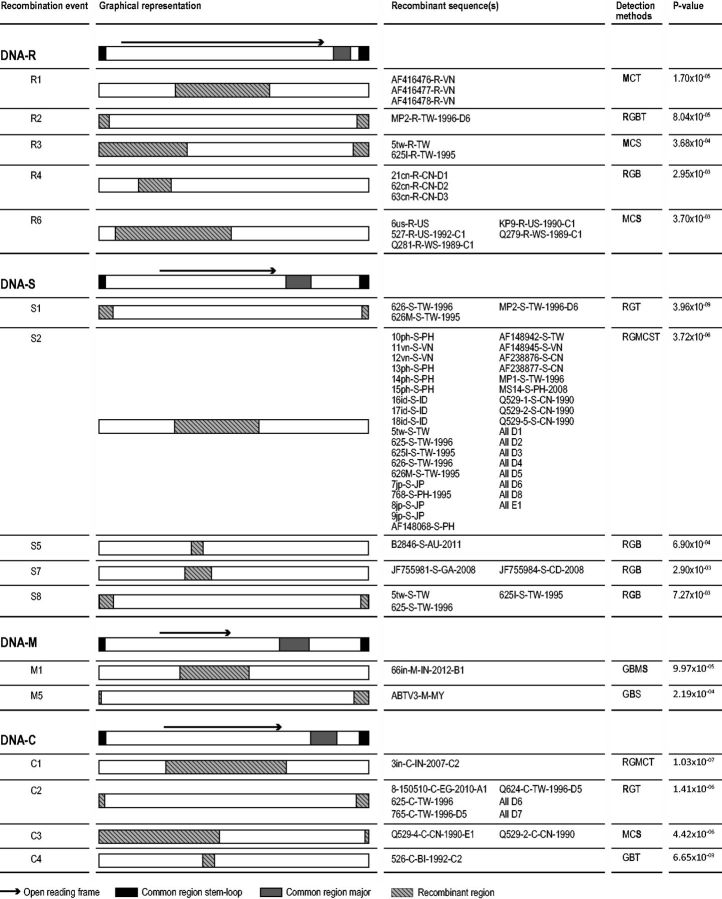
Recombination events detected in DNA-R, DNA-S, DNA-M, and DNA-C. Methods which detected the event are shown by abbreviations: R, RDP; G, GENCONV; B, BOOTSCAN; M, MAXCHI; C, CHIMERA; S, SISCAN; T, 3SEQ. The highest detected *P* value is shown with the detection method marked in bold. Further information on recombination events can be found in Supplementary Tables S3 and S5–S7.

**Figure 5.**
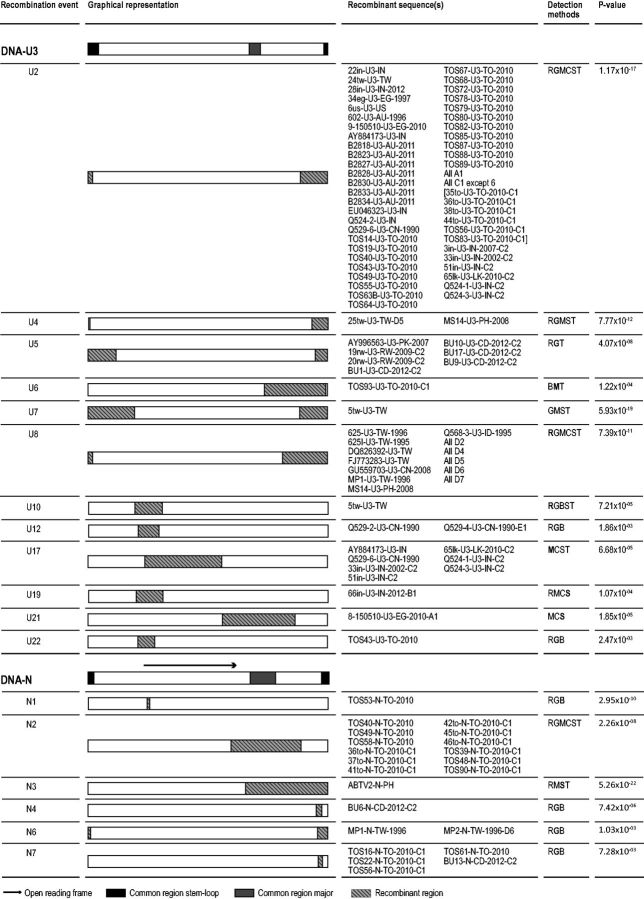
Recombination events detected in DNA-U3 and DNA-N. Methods which detected the event are shown by abbreviations: R, RDP; G, GENCONV; B, BOOTSCAN; M, MAXCHI; C, CHIMERA; S, SISCAN; T, 3SEQ. The highest detected *P* value is shown with the detection method marked in bold. Further information on recombination events can be found in Supplementary Tables S4 and S8.

These analyses revealed that all components displayed at least some evidence of recombination (Figs 4 and 5; Supplementary Tables S3–S8), with the greatest number of recombination events being detected in DNA-U3 (twelve events) and the fewest in DNA-M (two events).

All components carried evidence of recombinant regions involving the CR-SL region (with breakpoints falling within and/or on either side of this region) but only DNA-U3 and -N have recombination regions involving the CR-M, all of which had breakpoints falling on either side of this region. Of the 18 recombination events that were identified within multiple isolates, nine are seen within isolates from multiple countries. As with the reassortment events that are observed in multiple different genomes, these recombination events apparently occurred within genomes that were ancestral to two or more of the sequences analyzed here and indicate that at least some BBTV recombinants are epidemiologically relevant.

Twenty-two recombination events were detected within the components encoding genes of known function, DNA-R, -M, -N, -S, and -C. As has been found in previous nanovirus recombination studies (Hyder et al. 2011; Stainton et al. 2012; Grigoras et al. 2014; Savory and Ramakrishnan 2014), we detected similar numbers of recombination breakpoints within the non-coding and coding regions (twenty-four and twenty breakpoints, respectively).

In total, thirteen events resulted in recombinant genes that could express chimeric proteins. However, all thirteen of these events involved recombination between closely related BBTV variants, meaning that these recombination events would have had only a minimal impact on encoded protein amino acid sequences (Lefeuvre et al. 2009). Another possible sign of protein coding sequences having an impact on recombination patterns in BBTV is that the DNA-U3 component, which has no confirmed protein coding function, has a higher concentration of detectable recombination breakpoints than those of the known protein coding genes of other components. Interestingly DNA-U3 is also the component that appears to be most frequently exchanged by reassortment in BBTV. High frequencies of recombination in this component might reflect the fact that it is mostly evolving neutrally with no risk that recombinants might express defective chimeric proteins (Lefeuvre et al. 2009) and that there is therefore little conservation of coevolved epistatic interactions within this component.

Eighteen of the detected recombination events apparently involved the acquisition by BBTV isolates of genetic material derived through either inter-component recombination or recombination with non-BBTV babuvirus species (DNA-R, *n* = 2; -U3, *n* = 8; -S, *n* = 3; -C, *n* = 2; -N, *n* = 3) see Supplementary Tables S3–S8 for details. All of the recombinationally derived genome regions were analyzed using BLASTn (Altschul et al. 1990), with four of these regions—those transferred in U7 (in DNA-U3), S1 (in DNA-S), C2 (in DNA-C), and N3 (in DNA-N)—having potentially involved inter-component sequence transfers. BLASTn (Altschul et al. 1990) analysis of the U7 recombinant region indicated that this had likely involved a BBTV satellite (accession no. EU366175): a result that corroborates the finding of Fu et al*.* (2009). Although BLASTn analyses of events S1 and C2 indicated that the most likely sources of the recombinationally acquired sequences were BBTV DNA-M components, analysis of event N3 (which was detected in ABTV) indicated that it had likely involved a sequence transfer from an ABTV DNA-S component.

Our analyses indicated the remaining fourteen detected recombination events with unknown parents likely involved homologous recombination between BBTV and viruses belonging either to currently unsampled babuvirus species or to divergent currently unsampled BBTV strains. This suggests that there may exist a far greater diversity of BBTV-like babuvirus species (or perhaps divergent BBTV strains) than is presently known. Also, the fact that recombination events that are inferred to involve currently unsampled babuvirus species are primarily evident in BBTV isolates sampled in SEA/Far East region (nine of fourteen events) further suggests that this region is likely a major hotspot of ongoing recombination-driven BBTV diversification.

For recombination to occur between any particular pair of viruses, the viruses must have overlapping geographic ranges, host ranges, and cell tropisms. A number of plants, which are also hosts of *Pentalonia* spp. (*Pentalonia caladii* and *P. nigronervosa*), have been suggested as potential alternative hosts for BBTV including *Canna indica* (canna lily), *Hedychium coronarium* (white ginger lily), and *Colocasia esculenta* (taro) (Foottit et al. 2010; Duay et al. 2014). BBTV can be transmitted by *Pentalonia* spp. from an infected banana plant into *Co. esculenta* (asymptomatic) and then back into a healthy banana plant to cause disease (Ram and Summanwar 1984). *Canna indica* and *H. coronarium* have also shown weak to moderate reactions in BBTV-specific ELISA tests (Su, Wu, and Tsao 1992). However, although Pinili et al*.* (2013) reported the successful transmission of an Okinawan BBTV isolate to *C. indica, Co. esculenta,* and *Alpinia zerumbet*, further studies have failed to confirm that these species are suitable hosts for other BBTV strains (Hu et al. 1996; Geering and Thomas 1997; Manickam et al. 2002).

Regardless of the actual BBTV host-range, our results indicate that an increased sampling effort targeting uncultivated species in SEA/Far East and possibly India may lead to the identification of both alternative BBTV host species and numerous other epidemiologically relevant babuvirus species. Indeed, the only other known babuvirus species have been identified from this region: CBDV from India (Mandal et al. 2013) and ABTV from the Philippines and Malaysia (Sarawak) (Sharman et al. 2008).

### 3.5 Analysis of geographical structure within BBTV phylogenies

**Figure 6.**
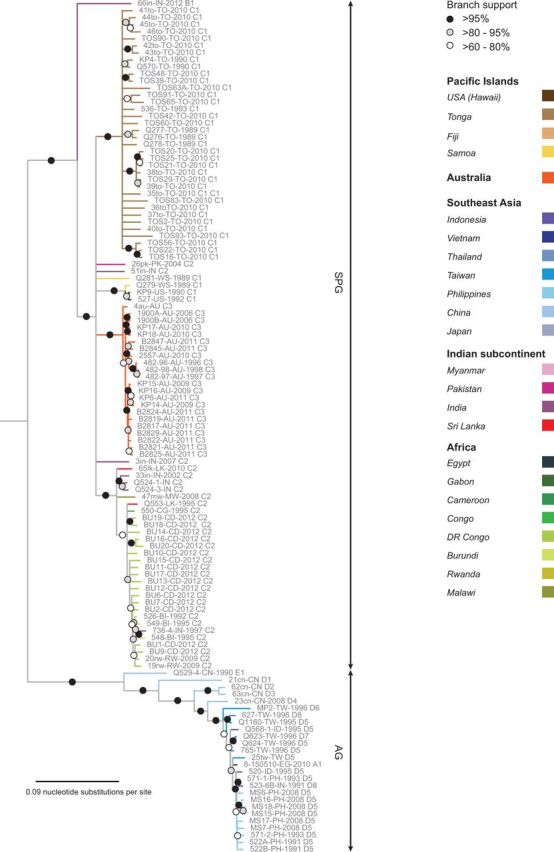
An RAxML tree of the FGD after all recombination and reassortment sequences were removed. ABTV was used to root the phylogenetic tree. Branches with <60 per cent bootstrap support have been collapsed. Full genomes are shown with isolate name, two letter country code, year of collection, and group name. GenBank accession numbers of the components which constitute each full genome can be found in Supplementary Table S1.

**Figure 7.**
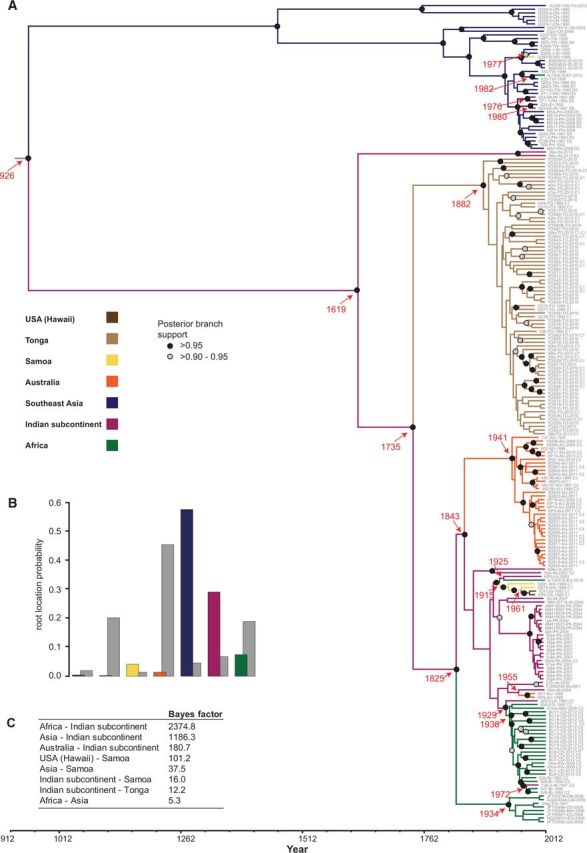
(A) An MCC tree constructed using the BBTV recombination-free CD (RF-CD) with a constant population size, strict-clock, discrete diffusion model. Branches are colored according to locations and the inferred dates (red font) of the statistically supported BBTV movement events are indicated with red arrows. Black circle on nodes indicate posterior branch support with >0.95 support and gray circles with >0.9–0.95 support. (B) Location probabilities for the root node of the tree are provided in the color-coded bar graph, and those obtained with randomization of the tip locations are shown as gray bars for each location. (C) The statistically supported epidemiological linkages between locations and their associated BF support values inferred using the Bayesian discrete diffusion phylogeography model are summarized.

Banana domestication is thought to have occurred on the Southeast Asian peninsula or its adjacent islands between 7 and 10,000 years ago (Denham et al. 2003; Perrier et al. 2011). Given the potential for human-mediated dissemination of BBTV via the subsequent worldwide movements of infected banana propagules, we aimed to examine the degree to which the phylogenetic relationships between BBTV isolates reflected their geographic origins. After removing genome components in the FGD dataset that had been derived through reassortment and fragments of components that had been derived through recombination in both the FGD and CSDs (to, respectively, yield recombination-free datasets, RF-FGD and RF-CSDs), we constructed ML phylogenetic trees for all of these datasets (Supplementary Figs S1–S6, Fig. 6). In order to date possible BBTV movement events and identify the likely origins of BBTV isolates in particular geographical regions, we additionally performed a Bayesian Monte Carlo Markov chain (MCMC) analysis and constructed an MCC tree (Fig. 7) from a recombination and reassortment-free dataset (RF-CD) representing 224 BBTV isolate sequences.

Although all of the sequences in the DNA-R, -S, -M, -C, and -N trees fell within clearly defined SPG and AG clades (Supplementary Figs 1 and S3–S6), the DNA-U3 tree contains some sequences that cannot be convincingly classified into either the SPG or AG clades (Supplementary Fig. S2).

Given the low degrees of support for most of the branches within the RF-CSD trees, we opted to focus on the RF-FGD ML and RF-CD MCC trees, in our assessment of finer scale geographic structure within each of the AG and SPG clades.

For the MCC tree, the MCMC analysis under a constant population size, strict-clock, discrete diffusion model produced an estimate of the BBTV mean nucleotide substitution rate of 2.916 × 10^−^^4^ substitutions/site/year (95% highest posterior density [HPD] 2.148 × 10^−^^4^–3.755 × 10^−^^4^), which is approximately double that reported in the analysis by Almeida et al*.* (2009) based on non-coding (∼500 nts) regions of the five components (DNA-R, DNA-S, DNA-M, DNA-C, and DNA-N) in Hawaiian isolates sampled between October and December 2005 (1.4 × 10^−^^4^ substitutions/site/year) but lower than that obtained under experimental conditions (3.9 × 10^−^^4^ substitutions/site/year) (Almeida et al. 2009). The time since the most recent common ancestor of the BBTV sequences represented in the MCC tree was 1,086 years (95% HPD 812—1,399 years).

It is immediately evident from both the RF-FGD ML (Fig. 6) and RF-CD MCC (Fig. 7) trees that there is a high degree of geographic clustering among the sub-clades within both the SPG and AG. That is, there are many well-supported monophyletic groups containing viruses all sampled from the same country. This clustering is particularly strong among the SPG isolates. For example, although the Tongan and Hawaiian SPG isolates form single well-supported monophyletic groups in both the MCC and ML trees, the Australian SPG isolates form a single cluster in the ML tree and two separate clusters in the MCC tree. This degree of clustering is indicative of these viruses all having originated from one or two founder viruses, a pattern that strongly supports the occurrence of infrequent BBTV introduction events into each of these countries/territories.

The Bayesian MCMC analysis depicted in the MCC tree indicated that the present global distribution of BBTV isolates could be accounted for by as few as fourteen individual movement events between eight statistically supported (i.e., with an associate BF > 5.0) pairs of locations (Fig. 7; Supplementary Fig. S7). Although the first of these events likely involved a movement from SEA to the Indian subcontinent approximately 1,000 years ago, the most recent involved a movement from SEA to Egypt approximately 30 years ago (Fig. 7, Supplementary Fig. S7, Supplementary Data—animated kml files).

Although SEA is a BBTV diversity hotspot and is identified in our analysis as the most probable location of the BBTV MRCA (probability = 0.557; Fig. 7), the Indian subcontinent was inferred to be the most highly connected location (involved in five of the eight statistically supported links) and is inferred to be a major hub of long-distance BBTV movements: it is both the major donor location for BBTV dispersal events to other parts of the world (seven of the fourteen supported movements) and the major recipient location of virus introductions (four of the fourteen movements). These include two dispersal events from the Indian subcontinent to Sub-Saharan Africa between 1825 and 1934, and one to Egypt (between 1929 and 1936), Australia (two events between 1843 and 1974), Tonga (one event between 1735 and 1882), and Samoa (one event between 1915 and 1934), and introduction events from SEA (the oldest between 926 and 1619, and two more recent events between 1976 and 1991) and Africa (between 1972 and 1997). Some of these movements may have been indirect. For example, reports indicate that BBTV most likely reached Australia from Fiji shortly before 1913 (Magee 1927). However, there were strong colonial and cultural links between India, Fiji, and Australia, and it is feasible that movement to Australia was from the Indian sub-continent to Australia, via Fiji. However, Fijian sequences are poorly represented in this study and may have failed to reveal such an intermediate step.

Other notable statistically supported viral dispersal events include one from Samoa to Hawaii between 1961 and 1978 (first disease report in Hawaii was in 1989) and SEA to Africa (between 1982 and 2010).

Therefore, although the global patterns of geographic structure that are evident within the BBTV phylogeny are not at all consistent with BBTV having been spread during the pre-historic dissemination of bananas across the globe, neither are they consistent with frequent, human-mediated trans-continental BBTV movements during the past few decades. They are, however, entirely consistent with more gradual, natural, or human-facilitated movements of the virus (via infected propagules and/or aphids) over the past 300 years from its centers of diversity in India, and SEA/Far East across the banana growing regions of the world. Additionally, there is clear evidence within our RF-FGD ML and RF-CD MCC trees, of frequent shorter distance BBTV movements. For example, in the AG clade sequences sampled from Taiwan, China, the Indian Subcontinent and Indonesia intermingle with one another within well-supported clades: a pattern which suggests that during the past 100 years there must have been multiple BBTV movements between these locations.

## 4 Concluding remarks

Here, we have studied the landscape of global BBTV diversity to reveal that the Indian Subcontinent, SEA, and the Far East are the current BBTV diversity hotspots. Accounting for recombination and reassortment, we phylogenetically analyzed 855 newly sequenced full BBTV genome components together with all available complete genome component sequences presently available in GenBank.

We find that the global distribution of BBTV genotypes is highly structured at the continental scale, suggesting that human-mediated inter-continental transfers of epidemiologically important BBTV genotypes have occurred relatively infrequently. Rather than the current global distribution of BBTV being attributable to either frequent long-distance movements of diseased banana planting material over the past few decades or the pre-historic spread of BBTV along with the spread of banana cultivation, our results suggest that the current distribution of the virus was primarily attained through infrequent movement events over the past 300 years primarily from its diversity hotspots in India and SEA.

## Supplementary data

Supplementary data is available at *VEVOLU Journal* online.

Supplementary Table S1
