# Supplementary material for: The global distribution of Banana bunchy top virus reveals little evidence for frequent recent, human-mediated long distance dispersal events
Source: Virus Evol. 2015 Sep 10;1(1):vev009. doi: 10.1093/ve/vev009 (PMC5014477; doi:10.1093/ve/vev009)
Supplement: Supplementary Table S1 [file Supplementary_legends.docx]

**Supplementary Figure 1:** Maximum likelihood phylogenetic tree constructed from the DNA-R recombination free dataset using the GTR+I+G4 nucleotide substitution model, rooted with DNA-R ABTV sequences. Branches with <60% bootstrap support have been collapsed. Sequences specifically highlighted in the text are shown with GenBank accession numbers, isolate names, two letter country codes, years of collection and group names where known.

**Supplementary Figure 2:** Maximum likelihood phylogenetic tree constructed from the BBTV DNA-U3 recombination free dataset using GTR+I+G4 nucleotide substitution model, rooted with DNA-U3 ABTV sequences. Branches with <60% bootstrap support have been collapsed. Sequences specifically highlighted in the text are shown with GenBank accession numbers, isolate names, two letter country codes, years of collection and group names where known.

**Supplementary Figure 3:** Maximum likelihood phylogenetic tree constructed from the BBTV DNA-S recombination free dataset using GTR+I+G4 nucleotide substitution model, rooted with DNA-S ABTV sequences. Branches with <60% bootstrap support have been collapsed. Sequences specifically highlighted in the text are shown with GenBank accession numbers, isolate names, two letter country codes, years of collection and group names where known.

**Supplementary Figure 4:** Maximum likelihood phylogenetic tree constructed from the BBTV DNA-M recombination free dataset using GTR+I+G4 nucleotide substitution model, rooted with DNA-M ABTV sequences. Branches with <60% bootstrap support have been collapsed. Sequences specifically highlighted in the text are shown with GenBank accession numbers, isolate names, two letter country codes, years of collection and group names where known.

**Supplementary Figure 5:** Maximum likelihood phylogenetic tree constructed from the BBTV DNA-C recombination free dataset using GTR+G4 nucleotide substitution model, rooted with DNA-C ABTV sequences. Branches with <60% bootstrap support have been collapsed. Sequences specifically highlighted in the text are shown with GenBank accession numbers, isolate names, two letter country codes, years of collection and group names where known.

**Supplementary Figure 6:** Maximum likelihood phylogenetic tree constructed from the BBTV DNA-N recombination free dataset using GTR+G4 nucleotide substitution model, rooted with DNA-N ABTV sequences. Branches with <60% bootstrap support have been collapsed. Sequences specifically highlighted in the text are shown with GenBank accession numbers, isolate names, two letter country codes, years of collection and group names where known.

**Supplementary Figure 7:** A maximum clade credibility tree constructed using the BBTV recombination free concatenated dataset (RF-CD) with a constant population size, strict-clock, discrete diffusion model. Branches are coloured according to locations and the inferred dates (red font) of the statistically-supported BBTV movement events are indicated with red arrows and 95% credibility HPD intervals shown in cyan coloured bars. Black circle on nodes indicate posterior branch support with >0.95 support and grey circles with >0.9 - 0.95 support.

**Supplementary Table 1:** BBTV isolate information for all sequences used in this study. In order to link components from the same sample all components are named with study code-component-country code-year-subgroup where known. Full genomes are shown in bold along with the corresponding subgroup. Accession numbers and references for all sequences are given. Countries of sample collection are identified by two letter country codes: AU-Australia, BI-Burundi, CD-Democratic Republic of Congo, CG-Congo, CM-Cameroon, CN-China, EG-Egypt, FJ-Fiji, GA-Gabon, ID-Indonesia, IN-India, JP-Japan, LK-Sri Lanka, MM-Myanmar, MW-Malawi, PH-Philippines, PK-Pakistan, RW-Rwanda, TO-Tonga, TW-Taiwan, US-United States of America [Hawaii], VN-Vietnam, WS- Samoa. ABTV sequence information is located at the bottom of the table.

**Supplementary Table 2:** Summary of reassortment detected in BBTV. All detection methods are shown with their corresponding P-values with the most significant shown in bold. Inferred minor parents are the sequences which are most likely to have contributed to the reassorted component, with inferred major parents contributing the remainder of the components.

**Supplementary Table 3:** Summary of recombination events detected in DNA-R components. All detection methods are shown with their corresponding P-values, with the most significant shown in bold.

**Supplementary Table 4:** Summary of recombination events detected in DNA-U3 components. All detection methods are shown with their corresponding P-values, with the most significant shown in bold.

**Supplementary Table 5:** Summary of recombination events detected in DNA-S components. All detection methods are shown with their corresponding P-values, with the most significant shown in bold.

**Supplementary Table 6:** Summary of recombination events detected in DNA-M components. All detection methods are shown with their corresponding P-values, with the most significant shown in bold.

**Supplementary Table 7:** Summary of recombination events detected in DNA-C components. All detection methods are shown with their corresponding P-values, with the most significant shown in bold.

**Supplementary Table 8:** Summary of recombination events detected in DNA-N components. All detection methods are shown with their corresponding P-values, with the most significant shown in bold.

**Dataset 1:** Google earth kml files of BBTV movement inferred using the RF-CD dataset.

**Dataset 2:** Sequence datasets used for the analysis.
