# Supplementary figures and images for: The global distribution of Banana bunchy top virus reveals little evidence for frequent recent, human-mediated long distance dispersal events
Source: Virus Evol. 2015 Sep 10;1(1):vev009. doi: 10.1093/ve/vev009 (PMC5014477; doi:10.1093/ve/vev009)

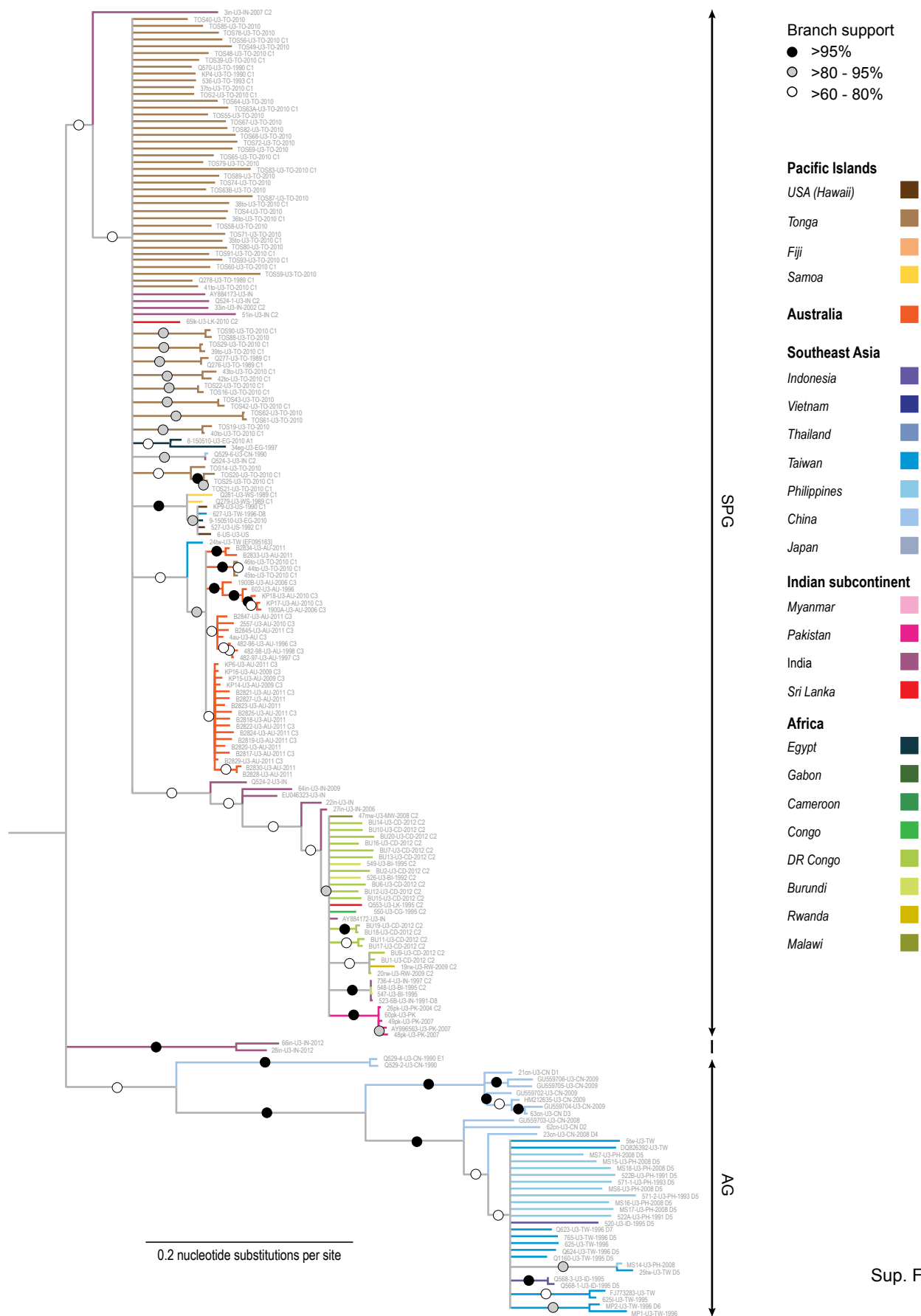

Supplement: Supplementary Table S1 [file Supp_Figure_2_A4.pdf]

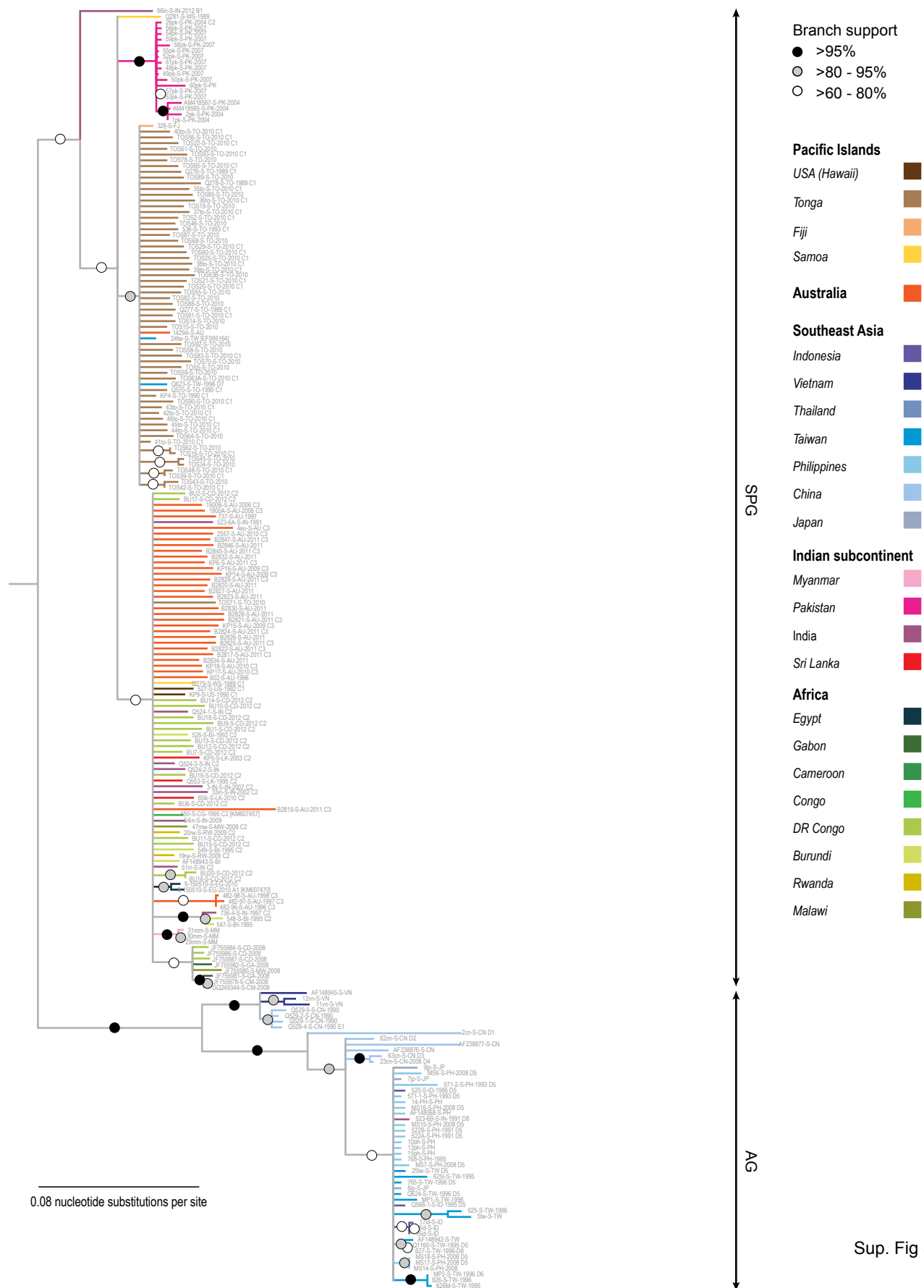

Sup. Fig 3

Supplement: Supplementary Table S1 [file Supp_Figure_3_A4.pdf]

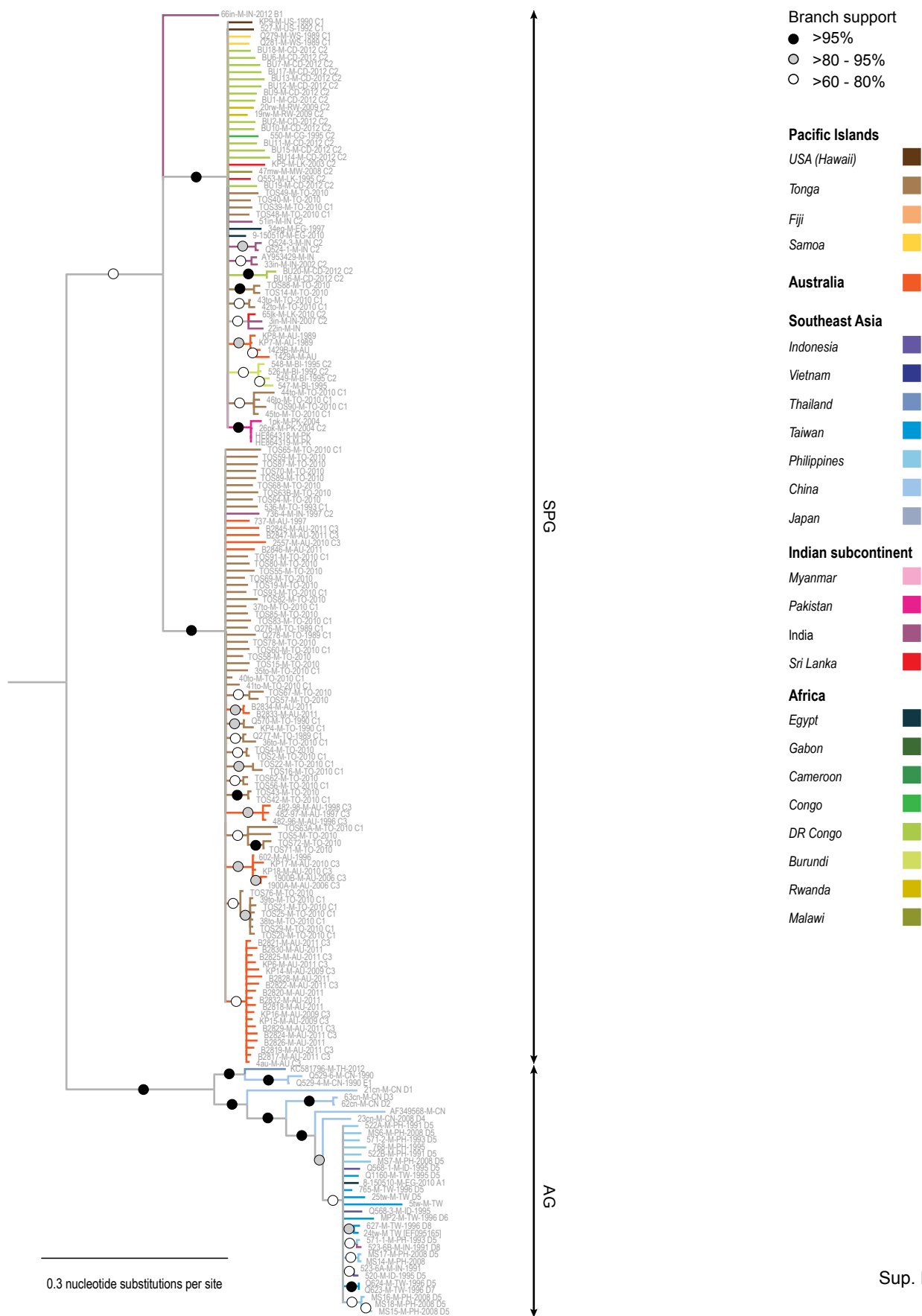

Sup. Fig 4

Supplement: Supplementary Table S1 [file Supp_Figure_4_A4.pdf]

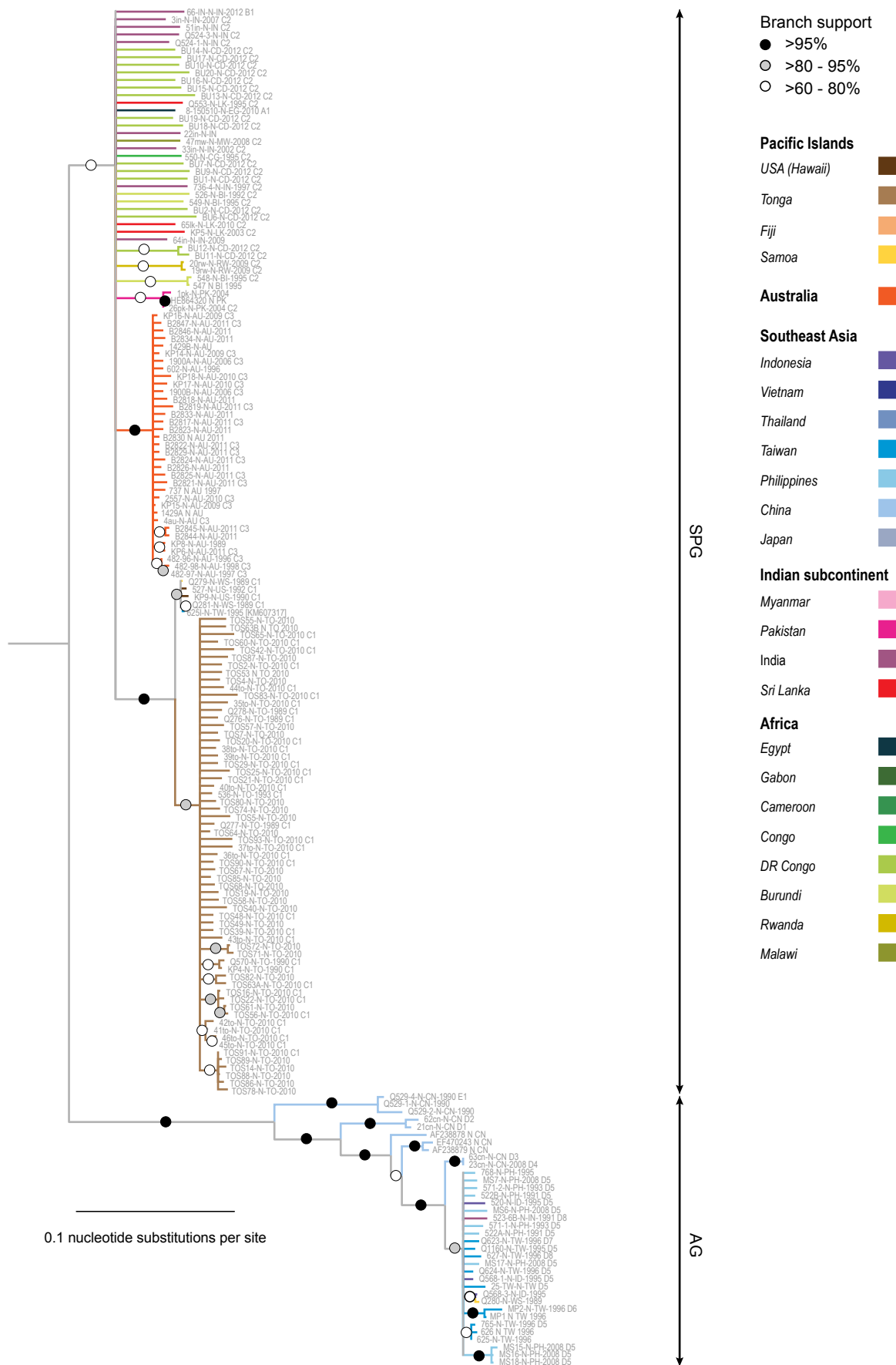

Sup. Fig 6

Supplement: Supplementary Table S1 [file Supp_Figure_6_A4.pdf]

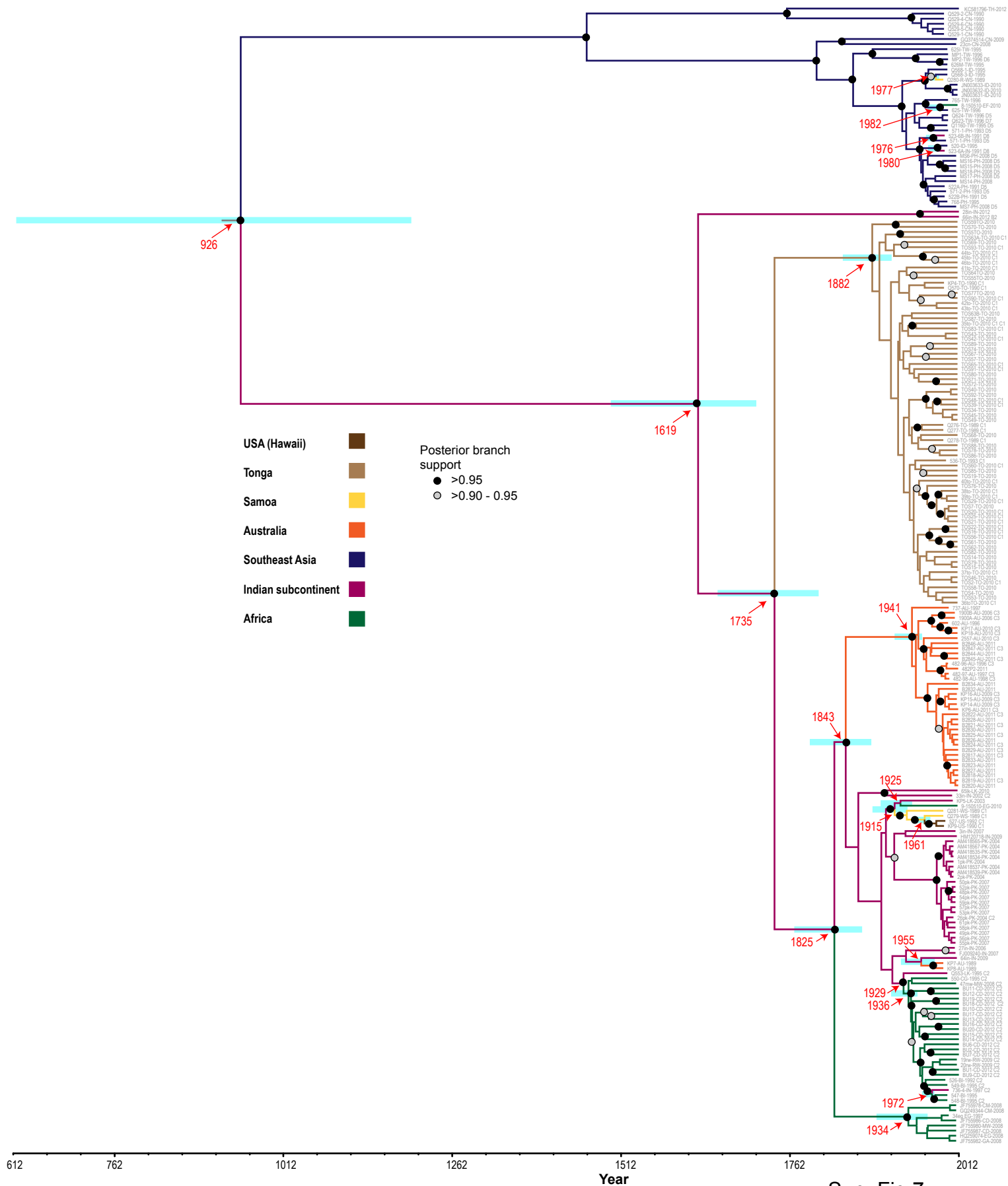

Supplement: Supplementary Table S1 [file Supp_Figure_7_A4.pdf]
