# Supplementary material for: The global distribution of Banana bunchy top virus reveals little evidence for frequent recent, human-mediated long distance dispersal events
Source: Virus Evol. 2015 Sep 10;1(1):vev009. doi: 10.1093/ve/vev009 (PMC5014477; doi:10.1093/ve/vev009)
Supplement: Supplementary Table S1 [file Supp_Figure_5_A4.pdf]

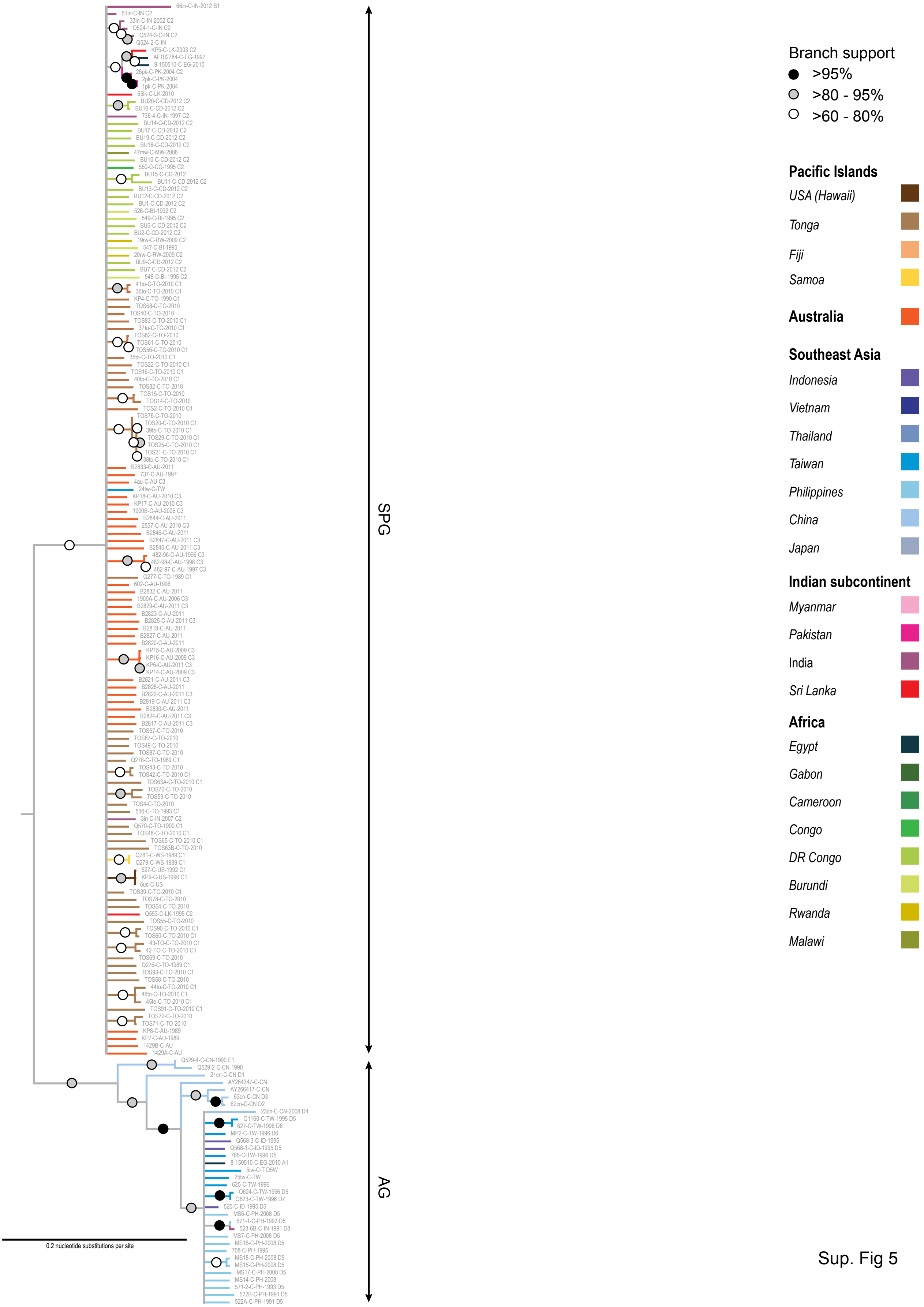
SPG
AG
0.2 nucleotide substitutions per site
Branch support
>95%
>80 - 95%
>60 - 80%
Pacific Islands
USA (Hawaii)
Tonga
Fiji
Samoa
Australia
Southeast Asia
Indonesia
Vietnam
Thailand
Taiwan
Philippines
China
Japan
Indian subcontinent
Myanmar
Pakistan
India
Sri Lanka
Africa
Egypt
Gabon
Cameroon
Congo
DR Congo
Burundi
Rwanda
Malawi

Sup. Fig 5
