# Supplementary material for: The global distribution of Banana bunchy top virus reveals little evidence for frequent recent, human-mediated long distance dispersal events
Source: Virus Evol. 2015 Sep 10;1(1):vev009. doi: 10.1093/ve/vev009 (PMC5014477; doi:10.1093/ve/vev009)
Supplement: Supplementary Table S1 [file Supp_Table_1.doc]

Supplementary Table 1

| **Country** | **Study**  **code** | **Isolate** | **Genome based grouping** | **Further isolate information** | **Sequence**  **reference** | **Year** | **DNA-R**  **Accession** | **DNA-U3**  **Accession** | **DNA-S**  **Accession** | **DNA-M**  **Accession** | **DNA-C**  **accession** | **DNA-N**  **Accession** |
| --- | --- | --- | --- | --- | --- | --- | --- | --- | --- | --- | --- | --- |
| AU | 737 | 737 |  | Pimpama, Queensland | This study | 1997 |  |  | KM607467 | KM607173 | KM607030 | KM607321 |
| **AU** | **2557** | **2557** | **C3** | **Nambour, Queensland** | **This study** | **2010** | **KM607588** | **KM607724** | **KM607442** | **KM607152** | **KM607009** | **KM607298** |
| AU | 1429A | 1429A |  | Currumbin, Queensland, Cavendish | This study |  |  |  | KM607439 | KM607148 | KM607005 | KM607294 |
| AU | 1429B | 1429B |  | Currumbin, Queensland Cavendish | This study |  | KM607585 |  |  | KM607149 | KM607006 | KM607295 |
| **AU** | **1900A** | **1900A** | **C3** | **Narangbah, Queensland** | **This study** | **2006** | **KM607586** | **KM607722** | **KM607440** | **KM607150** | **KM607007** | **KM607296** |
| **AU** | **1900B** | **1900B** | **C3** | **Narangbah, Queensland** | **This study** | **2006** | **KM607587** | **KM607723** | **KM607441** | **KM607151** | **KM607008** | **KM607297** |
| **AU** | **482-96** | **482-1996** | **C3** | **Nambour, Queensland Cavendish subgroup** | **This study** | **1996** | **KM607589** | **KM607725** | **KM607443** | **KM607153** | **KM607010** | **KM607299** |
| **AU** | **482-97** | **482-1997** | **C3** | **Nambour, Queensland Cavendish subgroup** | **This study** | **1997** | **KM607590** | **KM607726** | **KM607444** | **KM607154** | **KM607011** | **KM607300** |
| **AU** | **482-98** | **482-1998** | **C3** | **Nambour, Queensland Cavendish subgroup** | **This study** | **1998** | **KM607591** | **KM607727** | **KM607445** | **KM607155** | **KM607012** | **KM607301** |
| AU | 482P2 | 482P2 |  | **Nambour, Queensland Cavendish subgroup** Symptomless | This study | 2011 | KM607592 |  |  |  |  |  |
| **AU** | **4au** |  | **C3** | **Nambour, Queensland** |  |  | **S56276** | **L41576** | **L41574** | **L41575** | **L41578** | **L41577** |
| **AU** | **B2817** | **B2817** | **C3** | **The Channon, New South Wales** | **This study** | **2011** | **KM607614** | **KM607749** | **KM607472** | **KM607178** | **KM607035** | **KM607325** |
| AU | B2818 | B2818 |  | The Channon, New South Wales | This study | 2011 | KM607615 | KM607750 |  | KM607179 | KM607036 | KM607326 |
| **AU** | **B2819** | **B2819** | **C3** | **The Channon, New South Wales** | **This study** | **2011** | **KM607616** | **KM607751** | **KM607473** | **KM607180** | **KM607037** | **KM607327** |
| AU | B2820 | B2820 |  | The Channon, New South Wales | This study | 2011 | KM607617 |  | KM607474 | KM607181 | KM607038 |  |
| **AU** | **B2821** | **B2821** | **C3** | **The Channon, New South Wales** | **This study** | **2011** | **KM607618** | **KM607752** | **KM607475** | **KM607182** | **KM607039** | **KM607328** |
| **AU** | **B2822** | **B2822** | **C3** | **The Channon, New South Wales** | **This study** | **2011** | **KM607619** | **KM607753** | **KM607476** | **KM607183** | **KM607040** | **KM607329** |
| AU | B2823 | B2823 |  | The Channon, New South Wales | This study | 2011 | KM607620 | KM607754 | KM607477 |  | KM607041 | KM607330 |
| **AU** | **B2824** | **B2824** | **C3** | **The Channon, New South Wales** | **This study** | **2011** | **KM607621** | **KM607755** | **KM607478** | **KM607184** | **KM607042** | **KM607331** |
| **AU** | **B2825** | **B2825** | **C3** | **The Channon, New South Wales** | **This study** | **2011** | **KM607622** | **KM607756** | **KM607479** | **KM607185** | **KM607043** | **KM607332** |
| AU | B2826 | B2826 |  | The Channon, New South Wales | This study | 2011 | KM607623 |  | KM607480 | KM607186 |  | KM607333 |
| AU | B2827 | B2827 |  | The Channon, New South Wales | This study | 2011 | KM607624 | KM607757 | KM607481 |  | KM607044 |  |
| AU | B2828 | B2828 |  | The Channon, New South Wales | This study | 2011 | KM607625 |  | KM607482 | KM607187 | KM607045 |  |
| **AU** | **B2829** | **B2829** | **C3** | **The Channon, New South Wales** | **This study** | **2011** | **KM607626** | **KM607758** | **KM607483** | **KM607188** | **KM607046** | **KM607334** |
| AU | B2830 | B2830 |  | The Channon, New South Wales | This study | 2011 |  | KM607759 | KM607484 | KM607189 | KM607047 | KM607335 |
| AU | B2832 | B2832 |  | Tweed Valley, New South Wales | This study | 2011 | KM607627 |  | KM607485 | KM607190 | KM607048 |  |
| AU | B2833 | B2833 |  | Tweed Valley, New South Wales | This study | 2011 | KM607628 | KM607760 |  | KM607191 | KM607049 | KM607336 |
| AU | B2834 | B2834 |  | Tweed Valley, New South Wales | This study | 2011 | KM607629 | KM607761 | KM607486 | KM607192 |  | KM607337 |
| AU | B2844 | B2844 |  | Flaxton, Queensland | This study | 2011 | KM607630 |  |  |  | KM607050 | KM607338 |
| **AU** | **B2845** | **B2845** | **C3** | **Flaxton**, Queensland | **This study** | **2011** | **KM607631** | **KM607762** | **KM607487** | **KM607193** | **KM607051** | **KM607339** |
| AU | B2846 | B2846 |  | Yandina, Queensland | This study | 2011 | KM607632 |  | KM607488 | KM607194 | KM607052 | KM607340 |
| **AU** | **B2847** | **B2847** | **C3** | **Yandina**, Queensland | **This study** | **2011** | **KM607633** | **KM607763** | **KM607489** | **KM607195** | **KM607053** | **KM607341** |
| **AU** | **KP14** | **KP14** | **C3** | **Burringbah, New South Wales** | **This study** | **2009** | **KM607650** | **KM607780** | **KM607506** | **KM607212** | **KM607070** | **KM607358** |
| **AU** | **KP15** | **KP15** | **C3** | **Burringbah, New South Wales** | **This study** | **2009** | **KM607651** | **KM607781** | **KM607507** | **KM607213** | **KM607071** | **KM607359** |
| **AU** | **KP17** | **KP17** | **C3** | **Collected by D Peasley. Lady Finger cultivar** | **This study** | **2010** | **KM607653** | **KM607783** | **KM607509** | **KM607215** | **KM607073** | **KM607361** |
| **AU** | **KP18** | **KP18** | **C3** | **Collected by D Peasley. Lady Finger cultivar** | **This study** | **2010** | **KM607654** | **KM607784** | **KM607510** | **KM607216** | **KM607074** | **KM607362** |
| **AU** | **KP6** | **KP6** | **C3** | **New South Wales** | **This study** | **2011** | **KM607657** | **KM607786** | **KM607513** | **KM607219** | **KM607077** | **KM607365** |
| AU | KP7 | KP7 |  | Currumbin, Queensland. Cavendish | This study | 1989 | KM607658 |  |  | KM607220 | KM607078 |  |
| AU | KP8 | KP8 |  | Currumbin, Queensland. Cavendish | This study | 1989 | KM607659 |  |  | KM607221 | KM607079 | KM607366 |
| AU | 602 | 602 |  | Brisbane (Keperra), Queensland Cavendish subgroup | This study | 1996 |  | KM607741 | KM607460 | KM607170 | KM607026 | KM607315 |
| **AU** | **KP16** | **KP16** | **C3** | **Burringbah, New South Wales** | **This study** | **2009** | **KM607652** | **KM607782** | **KM607508** | **KM607214** | **KM607072** | **KM607360** |
| **BI** | **526** | **526** | **C2** | **Burundi** | **This study** | **1992** | **KM607598** | **KM607732** | **KM607451** | **KM607161** | **KM607017** | **KM607306** |
| BI | 547 | 547 |  | Symptomless | This study | 1995 |  | KM607735 | KM607454 | KM607164 | KM607020 | KM607309 |
| **BI** | **548** | **548** | **C2** |  | **This study** | **1995** | **KM607601** | **KM607736** | **KM607455** | **KM607165** | **KM607021** | **KM607310** |
| **BI** | **549** | **549** | **C2** |  | **This study** | **1995** | **KM607602** | **KM607737** | **KM607456** | **KM607166** | **KM607022** | **KM607311** |
| BI | AF148943 |  |  |  |  |  |  |  | AF148943 |  |  |  |
| **CD** | **BU1** | **BU1** | **C2** | **Kamanyola, South Kivu, Yangambi cultivar** | **This study** | **2012** | **KM607634** | **KM607764** | **KM607490** | **KM607196** | **KM607054** | **KM607342** |
| **CD** | **BU10** | **BU10** | **C2** | **Kisangani, Orientale, Libanga liaboelabokoy cultivar** | **This study** | **2012** | **KM607635** | **KM607765** | **KM607491** | **KM607197** | **KM607055** | **KM607343** |
| **CD** | **BU11** | **BU11** | **C2** | **Kisangani, Orientale, FHIA 03 cultivar** | **This study** | **2012** | **KM607636** | **KM607766** | **KM607492** | **KM607198** | **KM607056** | **KM607344** |
| **CD** | **BU12** | **BU12** | **C2** | **Kisangani, Orientale, Libanga likale cultivar** | **This study** | **2012** | **KM607637** | **KM607767** | **KM607493** | **KM607199** | **KM607057** | **KM607345** |
| **CD** | **BU13** | **BU13** | **C2** | **Kisangani, Orientale, Yangambi cultivar** | **This study** | **2012** | **KM607638** | **KM607768** | **KM607494** | **KM607200** | **KM607058** | **KM607346** |
| **CD** | **BU14** | **BU14** | **C2** | **Kisangani, Orientale, Yangambi cultivar** | **This study** | **2012** | **KM607639** | **KM607769** | **KM607495** | **KM607201** | **KM607059** | **KM607347** |
| **CD** | **BU15** | **BU15** | **C2** | **Kisangani, Orientale, Libanga likale cultivar** | **This study** | **2012** | **KM607640** | **KM607770** | **KM607496** | **KM607202** | **KM607060** | **KM607348** |
| **CD** | **BU16** | **BU16** | **C2** | **Kisangani, Orientale, Pisang awake cultivar** | **This study** | **2012** | **KM607641** | **KM607771** | **KM607497** | **KM607203** | **KM607061** | **KM607349** |
| **CD** | **BU17** | **BU17** | **C2** | **Kabondo, Orientale, Gros Michel cultivar** | **This study** | **2012** | **KM607642** | **KM607772** | **KM607498** | **KM607204** | **KM607062** | **KM607350** |
| **CD** | **BU18** | **BU18** | **C2** | **Kabondo, Orientale, Gros Michel cultivar** | **This study** | **2012** | **KM607643** | **KM607773** | **KM607499** | **KM607205** | **KM607063** | **KM607351** |
| **CD** | **BU19** | **BU19** | **C2** | **Kisangani, Orientale, Bisamunyo cultivar** | **This study** | **2012** | **KM607644** | **KM607774** | **KM607500** | **KM607206** | **KM607064** | **KM607352** |
| **CD** | **BU2** | **BU2** | **C2** | **Kamanyola, South Kivu, Yangambi cultivar** | **This study** | **2012** | **KM607645** | **KM607775** | **KM607501** | **KM607207** | **KM607065** | **KM607353** |
| **CD** | **BU20** | **BU20** | **C2** | **Kisangani, Orientale, Yangambi cultivar** | **This study** | **2012** | **KM607646** | **KM607776** | **KM607502** | **KM607208** | **KM607066** | **KM607354** |
| **CD** | **BU6** | **BU6** | **C2** | **Yangambi, Orientale, Libanga likale cultivar** | **This study** | **2012** | **KM607647** | **KM607777** | **KM607503** | **KM607209** | **KM607067** | **KM607355** |
| **CD** | **BU7** | **BU7** | **C2** | **Yangambi, Orientale, Yangambi cultivar** | **This study** | **2012** | **KM607648** | **KM607778** | **KM607504** | **KM607210** | **KM607068** | **KM607356** |
| **CD** | **BU9** | **BU9** | **C2** | **Yangambi, Orientale, Libanga likale cultivar** | **This study** | **2012** | **KM607649** | **KM607779** | **KM607505** | **KM607211** | **KM607069** | **KM607357** |
| CD | JF755984 | DRC-TV24.9 |  |  |  | 2008 |  |  | JF755984 |  |  |  |
| CD | JF755986 | DRC-23.3 |  |  |  | 2008 |  |  | JF755986 |  |  |  |
| CD | JF755987 | DRC-25.2 |  |  |  | 2008 |  |  | JF755987 |  |  |  |
| **CG** | **550** | **550** | **C2** |  | **This study** | **1995** | **KM607603** | **KM607738** | **KM607457** | **KM607167** | **KM607023** | **KM607312** |
| CM | GQ249344 |  |  |  | Unpublished | 2008 |  |  | GQ249344 |  |  |  |
| CM | JF755978 | CAM-TV4.1 |  |  |  | 2008 |  |  | JF755978 |  |  |  |
| **CN** | **21cn** | **Hainan** | **D1** |  |  |  | **AY450396** | **AY606084** | **AY494786** | **AY494788** | **AY606085** | **AY494787** |
| **CN** | **23cn** | **Haikou** | **D4** |  | **Unpublished** | **2008** | **FJ463042** | **FJ463043** | **FJ463044** | **FJ463045** | **FJ463046** | **FJ463047** |
| **CN** | **62cn** | **Haikou 4** | **D2** |  |  |  | **HQ378190** | **HM231314** | **HQ378191** | **HQ378192** | **HQ378193** | **HQ378194** |
| **CN** | **63cn** | **Haikou 2** | **D3** |  |  |  | **HQ616074** | **HQ616075** | **HQ616076** | **HQ616077** | **HQ616078** | **HQ616079** |
| CN | AF110266 | Zhangzhou |  |  |  |  | AF110266 |  |  |  |  |  |
| CN | AF238874 | NS strain |  |  |  |  | AF238874 |  |  |  |  |  |
| CN | AF238875 | NSP strain |  |  |  |  | AF238875 |  |  |  |  |  |
| CN | AF238876 | NS strain |  |  |  |  |  |  | AF238876 |  |  |  |
| CN | AF238877 | NSP strain |  |  |  |  |  |  | AF238877 |  |  |  |
| CN | AF238878 | NS strain |  |  |  |  |  |  |  |  |  | AF238878 |
| CN | AF238879 | NSP strain |  |  |  |  |  |  |  |  |  | AF238879 |
| CN | AF246123 | Guangdong-1 |  |  | Unpublished |  | AF246123 |  |  |  |  |  |
| CN | AF349568 | Zhangzhou |  |  | Unpublished |  |  |  |  | AF349568 |  |  |
| CN | AY264347 | NSP strain |  |  | Unpublished |  |  |  |  |  | AY264347 |  |
| CN | AY266417 | NS strain |  |  | Unpublished |  |  |  |  |  | AY266417 |  |
| CN | EF470243 | NSP strain |  |  | Unpublished |  |  |  |  |  |  | EF470243 |
| CN | GQ374514 | Zhangjiang |  |  | Unpublished | 2009 | GQ374514 |  |  |  |  |  |
| CN | GU559702 | ChengMai |  |  | Unpublished | 2009 |  | GU559702 |  |  |  |  |
| CN | GU559703 | LeDong |  |  | Unpublished | 2008 |  | GU559703 |  |  |  |  |
| CN | GU559704 | Haikou 1 |  |  | Unpublished | 2009 |  | GU559704 |  |  |  |  |
| CN | GU559705 | DanZhou |  |  | Unpublished | 2009 |  | GU559705 |  |  |  |  |
| CN | GU559706 | DanZhouHD |  |  | Unpublished | 2009 |  | GU559706 |  |  |  |  |
| CN | HM212635 | Haikou 3 |  |  | Unpublished | 2009 |  | HM212635 |  |  |  |  |
| CN | Q529-1 | Q529-1 |  |  | This study | 1990 | KM607676 |  | KM607533 |  |  | KM607385 |
| CN | Q529-2 | Q529-2 |  |  | This study | 1990 | KM607677 | KM607806 | KM607534 |  | KM607098 | KM607386 |
| **CN** | **Q529-4** | **Q529-4** | **E1** |  | **This study** | **1990** | **KM607678** | **KM607807** | **KM607535** | **KM607239** | **KM607099** | **KM607387** |
| CN | Q529-5 | Q529-5 |  |  | This study | 1990 |  |  | KM607536 |  |  |  |
| CN | Q529-6 | Q529-6 |  |  | This study | 1990 | KM607679 | KM607808 |  | KM607240 |  |  |
| CN | U97525 | C4 |  |  | Unpublished |  | U97525 |  |  |  |  |  |
| EG | 34eg | Kalubia |  |  | Unpublished | 1997 | AF102780 | AF102781 |  | AF102783 |  |  |
| **EG** | **8-150510** | **8-150510** | **A1** |  | **This study** | **2010** | **KM607612** | **KM607747** | **KM607470** | **KM607176** | **KM607033** | **KM607324** |
| EG | 9-150510 | 9-150510 |  |  | This study | 2010 | KM607613 | KM607748 | KM607471 | KM607177 | KM607034 |  |
| EG | AF102784 |  |  |  | Unpublished | 1997 |  |  |  |  | AF102784 |  |
| EG | AF416465 |  |  |  |  |  | AF416465 |  |  |  |  |  |
| EG | HQ259074 |  |  |  |  | 2008 | HQ259074 |  |  |  |  |  |
| FJ | 32fj |  |  |  | , |  | AF416466 |  | AF1489­44 |  |  |  |
| GA | JF755981 | GAB-TV18.2 |  |  |  | 2008 |  |  | JF755981 |  |  |  |
| GA | JF755982 | GAB-TV-17.5 |  |  |  | 2008 |  |  | JF755982 |  |  |  |
| **ID** | **520** | **520** | **D5** |  | **This study** | **1995** | **KM607593** | **KM607728** | **KM607446** | **KM607156** | **KM607013** | **KM607302** |
| ID | 16id | IG33 |  |  |  | 1998-2000 | AB186924 |  | AB186927 |  |  |  |
| ID | 17id | IG64 |  |  |  | 1998-2000 | AB186925 |  | AB186928 |  |  |  |
| ID | 18id | IJs11 |  |  |  | 1998-2000 | AB186926 |  | AB186929 |  |  |  |
| ID | JN003631 | Bali Tukad Badung II |  |  |  | 2010 | JN003631 |  |  |  |  |  |
| ID | JN003632 | Bali Tukad Petanu I |  |  |  | 2010 | JN003632 |  |  |  |  |  |
| ID | JN003633 | Bali Sempidi I |  |  |  | 2010 | JN003633 |  |  |  |  |  |
| **ID** | **Q568-1** | **Q568-1** | **D5** | **Cavendish** | **This study** | **1995** | **KM607681** | **KM607810** | **KM607538** | **KM607242** | **KM607101** | **KM607389** |
| ID | Q568-3 | Q568-3 |  | Apple Banana | This study | 1995 | KM607682 | KM607811 |  | KM607243 | KM607102 | KM607390 |
| IN | 22in | BT-1 |  |  | Unpublished |  |  | AY960129 |  | AY948439 |  | AY948438 |
| IN | 27in | Kerala 1 |  |  | Unpublished | 2006 | FJ009238 | FJ009239 |  |  |  |  |
| IN | 28in | Meghalaya |  |  | Unpublished | 2012 | JQ911667 | JQ911668 |  |  |  |  |
| **IN** | **33in** | **HB-TN** | **C2** |  |  | **2002** | **EU140342** | **EU140341** | **EU589459** | **EU190971** | **EU190969** | **EU190970** |
| **IN** | **3in** | **Lucknow** | **C2** |  |  | **2006-2007** | **DQ256267** | **EU402601** | **EF687856** | **EU516323** | **EU051379** | **EU391633** |
| **IN** | **51in** | **Bihar** | **C2** |  |  |  | **FJ605506** | **FJ605508** | **FJ605507** | **FJ609642** | **FJ609643** | **FJ609644** |
| IN | 523-6A | 523-6A |  |  | This study | 1991 | KM607596 |  | KM607449 | KM607159 |  |  |
| **IN** | **523-6B** | **523-6B** | **D8** |  | **This study** | **1991** | **KM607597** | **KM607731** | **KM607450** | **KM607160** | **KM607016** | **KM607305** |
| IN | 64in | Bangalore-GKVK |  |  | Unpublished | 2009 | JN243751 | JN243752 | JN243753 |  |  | JN243754 |
| **IN** | **66in** | **Umiam** | **B1** |  |  | **2012** | **KC119098** | **KC466373** | **KC466374** | **KC466375** | **KC466376** | **KC466377** |
| **IN** | **736-4** | **736-4** | **C2** |  | **This study** | **1997** | **KM607609** | **KM607745** | **KM607466** | **KM607172** | **KM607029** | **KM607320** |
| IN | AF416470 |  |  |  |  |  | AF416470 |  |  |  |  |  |
| IN | AY845437 |  |  |  | Unpublished |  | AY845437 |  |  |  |  |  |
| IN | AY884172 | BBTR1 |  |  |  |  |  | AY884172 |  |  |  |  |
| IN | AY884173 | BRJT9 |  |  |  |  |  | AY884173 |  |  |  |  |
| IN | AY953429 | TN |  |  | Unpublished |  |  |  |  | AY953429 |  |  |
| IN | DQ656118 | Kanpur |  |  | Unpublished |  | DQ656118 |  |  |  |  |  |
| IN | DQ656119 | Etawah |  |  | Unpublished |  | DQ656119 |  |  |  |  |  |
| IN | EU046323 | Bangalore |  |  | Unpublished |  |  | EU046323 |  |  |  |  |
| IN | FJ009240 | Kerala 2 |  |  | Unpublished | 2007 | FJ009240 |  |  |  |  |  |
| IN | HM120718 | Delhi |  |  | Unpublished | 2009 | HM120718 |  |  |  |  |  |
| **IN** | **Q524-1** | **Q524-1** | **C2** | **Poovan AB** | **This study** |  | **KM607674** | **KM607803** | **KM607530** | **KM607237** | **KM607095** | **KM607383** |
| IN | Q524-2 | Q524-2 |  | Robusta. Symptomless | This study |  |  | KM607804 | KM607531 |  | KM607096 |  |
| **IN** | **Q524-3** | **Q524-3** | **C2** | **Robusta** | **This study** |  | **KM607675** | **KM607805** | **KM607532** | **KM607238** | **KM607097** | **KM607384** |
| JP | 7jp | JN4 |  |  |  | 1999-2003 | AB108452 |  | AB108449 |  |  |  |
| JP | 8jp | JK3 |  |  |  | 1999-2003 | AB108453 |  | AB108450 |  |  |  |
| JP | 9jp | JY1 |  |  |  | 1999-2003 | AB108456 |  | AB108451 |  |  |  |
| JP | AB108454 | JM5 |  |  |  | 1999-2003 | AB108454 |  |  |  |  |  |
| JP | AB108455 | JM6 |  |  |  | 1999-2003 | AB108455 |  |  |  |  |  |
| JP | AB108457 | JY3 |  |  |  | 1999-2003 | AB108457 |  |  |  |  |  |
| JP | AB108458 | JY7 |  |  |  | 1999-2003 | AB108458 |  |  |  |  |  |
| **LK** | **65lk** | **Kandy** | **C2** |  | **Unpublished** | **2010** | **JN250593** | **JN250594** | **JN250595** | **JN250596** | **JN250597** | **JN250598** |
| **LK** | **Q553** | **Q553** | **C2** | **Mysore (AAB)** | **This study** | **1995** | **KM607680** | **KM607809** | **KM607537** | **KM607241** | **KM607100** | **KM607388** |
| MM | 29mm | My01 |  |  | Unpublished |  | AB252639 |  | AB252642 |  |  |  |
| MM | 30mm | MY02 |  |  | Unpublished |  | AB252640 |  | AB252643 |  |  |  |
| MM | 31mm | MY03 |  |  | Unpublished |  | AB252641 |  | AB252644 |  |  |  |
| MW | JF755980 | MAL-TV5.4 |  |  |  | 2008 |  |  | JF755980 |  |  |  |
| **MW** | **47mw** | **Malawi 73** | **C2** |  |  | **2008** | **JQ820453** | **JQ820454** | **JQ820455** | **JQ820456** | **JQ820457** | **JQ820458** |
| PH | 768 | 768 |  |  | This study | 1995 | KM607611 |  | KM607469 | KM607175 | KM607032 | KM607323 |
| PH | 10ph | bP5 |  |  |  |  | AB189067 |  | AB189068 |  |  |  |
| PH | 13ph | aP32 |  |  |  | 1999-2003 | AB250953 |  | AB250956 |  |  |  |
| PH | 14ph | aP34 |  |  |  | 1999-2003 | AB250954 |  | AB250957 |  |  |  |
| PH | 15ph | bP34 |  |  |  | 1999-2003 | AB250955 |  | AB250958 |  |  |  |
| **PH** | **522A** | **522A** | **D5** | **Abaca** | **This study** | **1991** | **KM607594** | **KM607729** | **KM607447** | **KM607157** | **KM607014** | **KM607303** |
| **PH** | **522B** | **522B** | **D5** | **Abaca** | **This study** | **1991** | **KM607595** | **KM607730** | **KM607448** | **KM607158** | **KM607015** | **KM607304** |
| **PH** | **571-1** | **571-1** | **D5** |  | **This study** | **1993** | **KM607604** | **KM607740** | **KM607459** | **KM607169** | **KM607025** | **KM607314** |
| **PH** | **571-2** | **571-2** | **D5** |  | **This study** | **1993** | **KM607605** | **KM607739** | **KM607458** | **KM607168** | **KM607024** | **KM607313** |
| PH | AF148068 |  |  |  |  |  |  |  | AF148068 |  |  |  |
| PH | AF416469 |  |  |  |  |  | AF416469 |  |  |  |  |  |
| PH | MS14 | MS14 |  | Abaca | This study | 2008 |  | KM607790 | KM607517 | KM607224 | KM607082 |  |
| **PH** | **MS15** | **MS15** | **D5** | **Abaca** | **This study** | **2008** | **KM607662** | **KM607791** | **KM607518** | **KM607225** | **KM607083** | **KM607370** |
| **PH** | **MS16** | **MS16** | **D5** | **Abaca** | **This study** | **2008** | **KM607663** | **KM607792** | **KM607519** | **KM607226** | **KM607084** | **KM607371** |
| **PH** | **MS17** | **MS17** | **D5** | **Abaca** | **This study** | **2008** | **KM607664** | **KM607793** | **KM607520** | **KM607227** | **KM607085** | **KM607372** |
| **PH** | **MS18** | **MS18** | **D5** | **Abaca** | **This study** | **2008** | **KM607665** | **KM607794** | **KM607521** | **KM607228** | **KM607086** | **KM607373** |
| **PH** | **MS6** | **MS6** | **D5** | **Los Banos. Fia cultivar** | **This study** | **2008** | **KM607666** | **KM607795** | **KM607522** | **KM607229** | **KM607087** | **KM607374** |
| **PH** | **MS7** | **MS7** | **D5** | **Los Banos. Lakatan Cultivar** | **This study** | **2008** | **KM607667** | **KM607796** | **KM607523** | **KM607230** | **KM607088** | **KM607375** |
| PK | 1pk | Thatha |  |  |  | 2004 | AM418538 |  | AM418566 | AM418541 | AM418569 | AM418568 |
| **PK** | **26pk** | **TJ1** | **C2** |  | **, ,** | **2004** | **AY996562** | **GQ214699** | **EF593169** | **EU095948** | **EF520722** | **EF529519** |
| PK | 2pk | Tandojam |  |  |  | 2004 | AM418536 |  | AM418540 |  | AM418564 |  |
| PK | 48pk | GH1 |  |  | , | 2007 | FJ859722 | FJ859748 | FJ859735 |  |  |  |
| PK | 49pk | JS1 |  |  | , | 2007 | FJ859732 | FJ859749 | FJ859745 |  |  |  |
| PK | 50pk | KP1 |  |  |  | 2007 | FJ859723 |  | FJ859736 |  |  |  |
| PK | 52pk | KP2 |  |  |  | 2007 | FJ859724 |  | FJ859737 |  |  |  |
| PK | 53pk | TA1 |  |  |  | 2007 | FJ859725 |  | Fj859738 |  |  |  |
| PK | 54pk | TA2 |  |  |  | 2007 | FJ859726 |  | FJ859739 |  |  |  |
| PK | 55pk | BS1 |  |  |  | 2007 | FJ859727 |  | FJ859740 |  |  |  |
| PK | 56pk | BS2 |  |  |  | 2007 | FJ859728 |  | FJ859741 |  |  |  |
| PK | 57pk | MT1 |  |  |  | 2007 | FJ859729 |  | FJ859742 |  |  |  |
| PK | 58pk | MT2 |  |  |  | 2007 | FJ859730 |  | FJ859743 |  |  |  |
| PK | 59pk | NS1 |  |  |  | 2007 | FJ859731 |  | FJ859744 |  |  |  |
| PK | 60pk | HD1 |  |  | , | 2007 | FJ859733 | FJ859750 | FJ859746 |  |  |  |
| PK | 61pk | HD2 |  |  |  | 2007 | FJ859734 |  | FJ859747 |  |  |  |
| PK | AM418534 | Nawabshah |  |  |  | 2004 | AM418534 |  |  |  |  |  |
| PK | AM418535 | Sakrand |  |  |  | 2004 | AM418535 |  |  |  |  |  |
| PK | AM418537 | Chambar |  |  |  | 2004 | AM418537 |  |  |  |  |  |
| PK | AM418539 | Hala |  |  |  | 2004 | AM418539 |  |  |  |  |  |
| PK | AM418565 | Kisanamari |  |  |  | 2004 |  |  | AM418565 |  |  |  |
| PK | AM418567 | Nasarpur |  |  |  | 2004 |  |  | AM418567 |  |  |  |
| PK | AY996563 | KHI |  |  |  | 2007 |  | AY996563 |  |  |  |  |
| PK | HE864318 |  |  |  |  |  |  |  |  | HE864318 |  |  |
| PK | HE864319 |  |  |  |  |  |  |  |  | HE864319 |  |  |
| PK | HE864320 |  |  |  |  |  |  |  |  |  |  | HE864320 |
| **RW** | **19rw** | **Rwanda 138** | **C2** |  |  | **2009** | **JQ820459** | **JQ820460** | **JQ820461** | **JQ820462** | **JQ820463** | **JQ820464** |
| **RW** | **20rw** | **Rwanda 142** | **C2** |  |  | **2009** | **JQ820465** | **JQ820466** | **JQ820467** | **JQ820468** | **JQ820469** | **JQ820470** |
| TH | KC581796 | Ubonratchathanee |  |  | Unpublished | 2012 |  |  |  | KC581796 |  |  |
| **TO** | **536** | **536** | **C1** |  | **This study** | **1993** | **KM607600** | **KM607734** | **KM607453** | **KM607163** | **KM607019** | **KM607308** |
| **TO** | **35to** | **TO166** | **C1** |  |  | **2010** | **JF957628** | **JF957640** | **JF957652** | **JF957664** | **JF957676** | **JF957688** |
| **TO** | **36to** | **TO114** | **C1** |  |  | **2010** | **JF957625** | **JF957637** | **JF957649** | **JF957661** | **JF957673** | **JF957685** |
| **TO** | **37to** | **TO121** | **C1** |  |  | **2010** | **JF957626** | **JF957638** | **JF957650** | **JF957662** | **JF957674** | **JF957686** |
| **TO** | **38to** | **TOS28** | **C1** |  |  | **2010** | **JF957636** | **JF957648** | **JF957660** | **JF957672** | **JF957684** | **JF957696** |
| **TO** | **39to** | **TOS12** | **C1** |  |  | **2010** | **JF957635** | **JF957647** | **JF957659** | **JF957671** | **JF957683** | **JF957695** |
| **TO** | **40to** | **TO124** | **C1** |  |  | **2010** | **JF957627** | **JF957639** | **JF957651** | **JF957663** | **JF957675** | **JF957687** |
| **TO** | **41to** | **TO306** | **C1** |  |  | **2010** | **JF957632** | **JF957644** | **JF957656** | **JF957668** | **JF957680** | **JF957692** |
| **TO** | **42to** | **TO314** | **C1** |  |  | **2010** | **JF957634** | **JF957646** | **JF957658** | **JF957670** | **JF957682** | **JF957694** |
| **TO** | **43to** | **TO310** | **C1** |  |  | **2010** | **JF957633** | **JF957645** | **JF957657** | **JF957669** | **JF957681** | **JF957693** |
| **TO** | **44to** | **TO208** | **C1** |  |  | **2010** | **JF957629** | **JF957641** | **JF957653** | **JF957665** | **JF957677** | **JF957689** |
| **TO** | **45to** | **TO224** | **C1** |  |  | **2010** | **JF957630** | **JF957642** | **JF957654** | **JF957666** | **JF957678** | **JF957690** |
| **TO** | **46to** | **TO290** | **C1** |  |  | **2010** | **JF957631** | **JF957643** | **JF957655** | **JF957667** | **JF957679** | **JF957691** |
| TO | AF416467 |  |  |  |  |  | AF416467 |  |  |  |  |  |
| **TO** | **KP4** | **KP4** | **C1** |  | **This study** | **1990** | **KM607655** | **KM607785** | **KM607511** | **KM607217** | **KM607075** | **KM607363** |
| **TO** | **Q276** | **Q276** | **C1** | **Banana** | **This study** | **1989** | **KM607669** | **KM607798** | **KM607525** | **KM607232** | **KM607090** | **KM607377** |
| **TO** | **Q277** | **Q277** | **C1** | **Banana** | **This study** | **1989** | **KM607670** | **KM607799** | **KM607526** | **KM607233** | **KM607091** | **KM607378** |
| **TO** | **Q278** | **Q278** | **C1** | **Banana** | **This study** | **1989** | **KM607671** | **KM607800** | **KM607527** | **KM607234** | **KM607092** | **KM607379** |
| **TO** | **Q570** | **Q570** | **C1** |  | **This study** | **1990** | **KM607683** | **KM607812** | **KM607539** | **KM607244** | **KM607103** | **KM607391** |
| TO | TOS14 | TOS14 |  | Collected by Halapua Hakalo. Cultivar- Siaine. Tongatapu, village Kolofo'ou | This study | 2010 |  | KM607815 | KM607542 | KM607247 | KM607106 | KM607394 |
| TO | TOS15 | TOS15 |  | Collected by Halapua Hakalo. Cultivar- Siaine. Tongatapu, village Kolofo'ou | This study | 2010 | KM607686 |  | KM607543 | KM607248 | KM607107 |  |
| **TO** | **TOS16** | **TOS16** | **C1** | **Collected by Mosese Lui. Cultivar- Hopa. Tongatapu, village Fua'amotu** | **This study** | **2010** | **KM607687** | **KM607816** | **KM607544** | **KM607249** | **KM607108** | **KM607395** |
| TO | TOS19 | TOS19 |  | Collected by Takia Pifeleti. Cultivar- Banana. Tongatapu, village Kapetā | This study | 2010 | KM607688 | KM607817 | KM607545 | KM607250 |  | KM607396 |
| **TO** | **TOS2** | **TOS2** | **C1** | **Collected by Takia Pifeleti. Cultivar- Banana. Tongatapu, village Kapetā** | **This study** | **2010** | **KM607689** | **KM607818** | **KM607546** | **KM607251** | **KM607109** | **KM607397** |
| **TO** | **TOS20** | **TOS20** | **C1** | **Collected by Vaivelata Salesio. Cultivar- Banana. Tongatapu, village Mau'ofanga** | **This study** | **2010** | **KM607690** | **KM607819** | **KM607547** | **KM607252** | **KM607110** | **KM607398** |
| **TO** | **TOS21** | **TOS21** | **C1** | **Collected by Vaivelata Salesio. Cultivar- Banana. Tongatapu, village Mau'ofanga** | **This study** | **2010** | **KM607691** | **KM607820** | **KM607548** | **KM607253** | **KM607111** | **KM607399** |
| **TO** | **TOS22** | **TOS22** | **C1** | **Collected by Mosese Lui. Cultivar- Hopa. Tongatapu, village Fua'amotu** | **This study** | **2010** | **KM607692** | **KM607821** | **KM607549** | **KM607254** | **KM607112** | **KM607400** |
| **TO** | **TOS25** | **TOS25** | **C1** | **Collected by Fetu'u. Cultivar- Banana. Tongatapu, village Kolomotuá** | **This study** | **2010** | **KM607693** | **KM607822** | **KM607550** | **KM607255** | **KM607113** | **KM607401** |
| **TO** | **TOS29** | **TOS29** | **C1** | **Collected by Likamonū Hua'kau. Cultivar- Banana. Tongatapu, village Āhau** | **This study** | **2010** | **KM607694** | **KM607823** | **KM607551** | **KM607256** | **KM607114** | **KM607402** |
| TO | TOS34 | TOS34 |  | Collected by Ofa Pakofe. Cultivar- Siaine. Tongatapu, village Tokomololo | This study | 2010 | KM607695 |  | KM607552 |  |  |  |
| **TO** | **TOS39** | **TOS39** | **C1** | **Collected by Sīmisi Tonga. Cultivar- Hopa. Tongatapu, village Hu'atolitoli** | **This study** | **2010** | **KM607696** | **KM607824** | **KM607553** | **KM607257** | **KM607115** | **KM607403** |
| TO | TOS4 | TOS4 |  | Collected by Viliami Látū. Cultivar- Banana. Tongatapu, village Haáteiho | This study | 2010 | KM607697 | KM607825 |  | KM607258 | KM607116 | KM607404 |
| TO | TOS40 | TOS40 |  | Collected by Vilivaea Teisina. Cultivar- Misipeka. Tongatapu, village Ha'alalo | This study | 2010 | KM607698 | KM607826 |  | KM607259 | KM607117 | KM607405 |
| **TO** | **TOS42** | **TOS42** | **C1** | **Collected by A Piutau. Cultivar- Siaine. Tongatapu, village Kolomotu'a** | **This study** | **2010** | **KM607699** | **KM607827** | **KM607554** | **KM607260** | **KM607118** | **KM607406** |
| TO | TOS43 | TOS43 |  | Collected by A Piutau. Cultivar- Hopa. Tongatapu, village Kolomotu'a | This study | 2010 |  | KM607828 | KM607555 | KM607261 | KM607119 |  |
| TO | TOS45 | TOS45 |  | Collected by Vilivaea Teisina. Cultivar- Siaine. Tongatapu, village Ha'alalo | This study | 2010 | KM607700 |  | KM607556 |  |  |  |
| TO | TOS46 | TOS46 |  | Collected by Alikei Langa'oi. Cultivar- Siaine. Tongatapu, village Tokomololo | This study | 2010 | KM607701 |  | KM607557 |  |  |  |
| **TO** | **TOS48** | **TOS48** | **C1** | **Collected by Semi Mafi. Cultivar- Hopa. Tongatapu, village Umusi** | **This study** | **2010** | **KM607702** | **KM607829** | **KM607558** | **KM607262** | **KM607120** | **KM607407** |
| TO | TOS49 | TOS49 |  | Collected by Semi Mafi. Tongatapu, village Umusi | This study | 2010 | KM607703 | KM607830 |  | KM607263 | KM607121 | KM607408 |
| TO | TOS5 | TOS5 |  | Collected by Lingitoni Manu. Cultivar- Banana. Tongatapu, village Popua | This study | 2010 | KM607704 |  | KM607559 | KM607264 |  | KM607409 |
| TO | TOS53 | TOS53 |  | Collected by Tevita Tu'ipulotu. Cultivar- Banana. Tongatapu, village Tofoa | This study | 2010 |  |  |  |  |  | KM607410 |
| TO | TOS55 | TOS55 |  | Tongatapu, village Halaleva | This study | 2010 |  | KM607831 | KM607560 | KM607265 | KM607122 | KM607411 |
| **TO** | **TOS56** | **TOS56** | **C1** | **Tongatapu, village Kolofo'ou** | **This study** | **2010** | **KM607705** | **KM607832** | **KM607561** | **KM607266** | **KM607123** | **KM607412** |
| TO | TOS57 | TOS57 |  | Tongatapu, village Kolomotu'a | This study | 2010 | KM607706 |  |  | KM607267 | KM607124 | KM607413 |
| TO | TOS58 | TOS58 |  | Tongatapu, village Hofoa | This study | 2010 |  | KM607833 | KM607562 | KM607268 | KM607125 | KM607414 |
| TO | TOS59 | TOS59 |  | Tongatapu, village Havelu | This study | 2010 |  | KM607834 | KM607563 | KM607269 | KM607126 |  |
| **TO** | **TOS60** | **TOS60** | **C1** | **Tongatapu, village Halao'vave** | **This study** | **2010** | **KM607707** | **KM607835** | **KM607564** | **KM607270** | **KM607127** | **KM607415** |
| TO | TOS61 | TOS61 |  | Tongatapu, village Popua | This study | 2010 |  | KM607836 | KM607565 |  | KM607128 | KM607416 |
| TO | TOS62 | TOS62 |  | Tongatapu, village Mau'ofanga | This study | 2010 | KM607708 | KM607837 | KM607566 | KM607271 | KM607129 |  |
| **TO** | **TOS63A** | **TOS63A** | **C1** | **Tongatapu, village Nautoka** | **This study** | **2010** | **KM607709** | **KM607838** | **KM607567** | **KM607272** | **KM607130** | **KM607417** |
| TO | TOS63B | TOS63B |  | Tongatapu, village Ha'asini | This study | 2010 |  | KM607839 | KM607568 | KM607273 | KM607131 | KM607418 |
| TO | TOS64 | TOS64 |  | Tongatapu, village Nilifoua | This study | 2010 |  | KM607840 | KM607569 | KM607274 | KM607132 | KM607419 |
| **TO** | **TOS65** | **TOS65** | **C1** | **Tongatapu, village Fua'amotu** | **This study** | **2010** | **KM607710** | **KM607841** | **KM607570** | **KM607275** | **KM607133** | **KM607420** |
| TO | TOS67 | TOS67 |  | Tongatapu, village Kolonga | This study | 2010 |  | KM607842 |  | KM607276 | KM607134 | KM607421 |
| TO | TOS68 | TOS68 |  | Tongatapu, village Tatakamoetonga | This study | 2010 |  | KM607843 | KM607571 | KM607277 | KM607135 | KM607422 |
| TO | TOS69 | TOS69 |  | Tongatapu, village Mua | This study | 2010 |  | KM607844 |  | KM607278 | KM607136 |  |
| TO | TOS7 | TOS7 |  | Collected by Lotohua Pulini. Cultivar- Siane. Tongatapu, village Ha'ateiho | This study | 2010 | KM607711 |  |  |  |  | KM607423 |
| TO | TOS70 | TOS70 |  | Tongatapu, village Hoi | This study | 2010 |  |  | KM607572 | KM607279 | KM607137 |  |
| TO | TOS71 | TOS71 |  | Tongatapu, village Alaki | This study | 2010 |  | KM607845 | KM607573 | KM607280 | KM607138 | KM607424 |
| TO | TOS72 | TOS72 |  | Tongatapu, village Makaunga | This study | 2010 | KM607712 | KM607846 |  | KM607281 | KM607139 | KM607425 |
| TO | TOS74 | TOS74 |  | Tongatapu, village Pea | This study | 2010 |  | KM607847 |  |  |  | KM607426 |
| TO | TOS76 | TOS76 |  | Tongatapu, village Ha'akame | This study | 2010 | KM607713 |  |  | KM607282 | KM607140 |  |
| TO | TOS77 | TOS77 |  | Tongatapu, village Tokomololo | This study | 2010 | KM607714 |  |  |  |  |  |
| TO | TOS78 | TOS78 |  | Tongatapu, village Folaha | This study | 2010 |  | KM607848 | KM607574 | KM607283 | KM607141 | KM607427 |
| TO | TOS79 | TOS79 |  | Tongatapu, village Ha'ateiho | This study | 2010 |  | KM607849 |  |  |  |  |
| TO | TOS80 | TOS80 |  | Tongatapu, village Longoteme | This study | 2010 |  | KM607850 |  | KM607284 |  | KM607428 |
| TO | TOS82 | TOS82 |  | Tongatapu, village Houma | This study | 2010 |  | KM607851 | KM607575 | KM607285 | KM607142 | KM607429 |
| **TO** | **TOS83** | **TOS83** | **C1** | **Tongatapu, village Utulau** | **This study** | **2010** | **KM607715** | **KM607852** | **KM607576** | **KM607286** | **KM607143** | **KM607430** |
| TO | TOS85 | TOS85 |  | Tongatapu, village Kolovaj | This study | 2010 | KM607716 | KM607853 | KM607577 | KM607287 |  | KM607431 |
| TO | TOS86 | TOS86 |  | Tongatapu, village Fatai | This study | 2010 | KM607717 |  |  |  |  | KM607432 |
| TO | TOS87 | TOS87 |  | Tongatapu, village Masilamea | This study | 2010 |  | KM607854 | KM607578 | KM607288 | KM607144 | KM607433 |
| TO | TOS88 | TOS88 |  | Tongatapu, village Nukunuku | This study | 2010 | KM607718 | KM607855 | KM607579 | KM607289 |  | KM607434 |
| TO | TOS89 | TOS89 |  | Tongatapu, village Fo'ui | This study | 2010 |  | KM607856 | KM607580 | KM607290 |  | KM607435 |
| **TO** | **TOS90** | **TOS90** | **C1** | **Tongatapu, village Matahau** | **This study** | **2010** | **KM607719** | **KM607857** | **KM607581** | **KM607291** | **KM607145** | **KM607436** |
| **TO** | **TOS91** | **TOS91** | **C1** | **Tongatapu, village Lakepa** | **This study** | **2010** | **KM607720** | **KM607858** | **KM607582** | **KM607292** | **KM607146** | **KM607437** |
| TO | TOS92 | TOS92 |  | Tongatapu, village Tofoa | This study | 2010 |  |  | KM607583 |  |  |  |
| **TO** | **TOS93** | **TOS93** | **C1** | **Tongatapu, village Vaololoa** | **This study** | **2010** | **KM607721** | **KM607859** | **KM607584** | **KM607293** | **KM607147** | **KM607438** |
| TW | 625 | 625 |  |  | This study | 1996 | KM607606 | KM607742 | KM607461 |  | KM607027 | KM607316 |
| TW | 626 | 626 |  | Mild isolate | This study | 1996 |  |  | KM607463 |  |  | KM607318 |
| **TW** | **627** | **627** | **D8** | **Symptomless** | **This study** | **1996** | **KM607608** | **KM607744** | **KM607465** | **KM607171** | **KM607028** | **KM607319** |
| **TW** | **765** | **765** | **D5** |  | **This study** | **1996** | **KM607610** | **KM607746** | **KM607468** | **KM607174** | **KM607031** | **KM607322** |
| TW | 24tw | V-1 |  |  |  |  | EF095161 | EF095163 | EF095164 | EF095165 | EF095166 |  |
| **TW** | **25tw** | **Severe phenotype** | **D5** |  |  |  | **DQ826390** | **DQ826391** | **DQ826393** | **DQ826394** | **DQ826395** | **DQ826396** |
| TW | 5tw | TW3 |  |  |  |  | EU366169 | EU366170 | EU366171 | EU366172 | EU366173 |  |
| TW | 625I | 625I |  | Sample ex HJ Su. Banana | This study | 1995 | KM607607 | KM607743 | KM607462 |  |  | KM607317 |
| TW | 626M | 626M |  | Sample ex HJ Su. Banana | This study | 1995 |  |  | KM607464 |  |  |  |
| TW | AF148942 |  |  |  |  |  |  |  | AF148942 |  |  |  |
| TW | AF416468 |  |  |  |  |  | AF416468 |  |  |  |  |  |
| TW | DQ826392 | Severe strain clone b |  |  |  |  |  | DQ826392 |  |  |  |  |
| TW | EF095162 | V-1 clone a |  |  |  |  | EF095162 |  |  |  |  |  |
| TW | FJ773283 | TW3 clone a |  |  |  |  |  | FJ773283 |  |  |  |  |
| TW | MP1 | MP1 |  | Mild isolate | This study | 1996 |  | KM607788 | KM607515 |  |  | KM607368 |
| **TW** | **MP2** | **MP2** | **D6** | **Mild isolate** | **This study** | **1996** | **KM607661** | **KM607789** | **KM607516** | **KM607223** | **KM607081** | **KM607369** |
| **TW** | **Q1160** | **Q1160** | **D5** | **Sample ex HJ Su** | **This study** | **1995** | **KM607668** | **KM607797** | **KM607524** | **KM607231** | **KM607089** | **KM607376** |
| **TW** | **Q623** | **Q623** | **D7** | **Severe isolate** | **This study** | **1996** | **KM607684** | **KM607813** | **KM607540** | **KM607245** | **KM607104** | **KM607392** |
| **TW** | **Q624** | **Q624** | **D5** | **Severe isolate** | **This study** | **1996** | **KM607685** | **KM607814** | **KM607541** | **KM607246** | **KM607105** | **KM607393** |
| **US** | **527** | **527** | **C1** | **Hawaii** | **This study** | **1992** | **KM607599** | **KM607733** | **KM607452** | **KM607162** | **KM607018** | **KM607307** |
| US | 6us | Hawaiian |  |  |  |  | U18077 | U18078 |  |  | U18079 |  |
| **US** | **KP9** | **KP9** | **C1** | **Hawaii, Oahu Island. Williams cultivar** | **This study** | **1990** | **KM607660** | **KM607787** | **KM607514** | **KM607222** | **KM607080** | **KM607367** |
| VN | 11vn | Isolate V6 |  |  |  |  | AB113659 |  | AB113661 |  |  |  |
| VN | 12vn | Isolate V14 |  |  |  |  | AB113660 |  | AB113662 |  |  |  |
| VN | AF148945 |  |  |  |  |  |  |  | AF148945 |  |  |  |
| VN | AF416464 |  |  |  |  |  | AF416464 |  |  |  |  |  |
| VN | AF416472 | Son La region |  |  |  |  | AF416472 |  |  |  |  |  |
| VN | AF416473 | Dien Bien Phu region |  |  |  |  | AF416473 |  |  |  |  |  |
| VN | AF416474 | Bac Ninh region |  |  |  |  | AF416474 |  |  |  |  |  |
| VN | AF416475 | Hue region |  |  |  |  | AF416475 |  |  |  |  |  |
| VN | AF416476 | Buon Ma Thout Region |  |  |  |  | AF416476 |  |  |  |  |  |
| VN | AF416477 | Da Nang region |  |  |  |  | AF416477 |  |  |  |  |  |
| VN | AF416478 | Ho Chi Minh City region |  |  |  |  | AF416478 |  |  |  |  |  |
| VN | AF416479 | Yen Bai region |  |  |  |  | AF416479 |  |  |  |  |  |
| **WS** | **Q279** | **Q279** | **C1** | **Banana** | **This study** | **1989** | **KM607672** | **KM607801** | **KM607528** | **KM607235** | **KM607093** | **KM607380** |
| WS | Q280 | Q280 |  | Banana | This study | 1989 |  |  |  |  |  | KM607381 |
| **WS** | **Q281** | **Q281** | **C1** | **Banana** | **This study** | **1989** | **KM607673** | **KM607802** | **KM607529** | **KM607236** | **KM607094** | **KM607382** |
| **MY** | **ABTV1** | **Q767** |  |  |  |  | **EF546813** | **EF546809** | **EF546810** | **EF546811** | **EF546812** | **EF546808** |
| **PH** | **ABTV2** | **Q1108** |  |  |  |  | **EF546807** | **EF546803** | **EF546804** | **EF546805** | **EF546806** | **EF546802** |
| MY | ABTV3 | Malaysian strain |  |  |  |  |  |  |  | AF102148 |  |  |

1. Abdel-Salam SM, Dahot MU, Sadik AS (2012) Molecular comparative analysis of component 1 (DNA-R) of an Egyptian isolate of banana bunchy top nanovirus isolated from banana aphid (Pentalonia nigronervosa). Journal of Genetic Engineering and Biotechnology 10:55-65

2. Amin I, Qazi J, Mansoor S, Ilyas M, Briddon RW (2008) Molecular characterisation of Banana bunchy top virus (BBTV) from Pakistan. Virus Genes 36:191-198

3. Anandhi J, Vijila C, Viswanath GS, Lokeswari TS (2007) Screening banana plants for banana bunchy top virus with primers specific to Indian isolates. Journal of Plant Diseases and Protection 114:101-107

4. Banerjee A, Roy S, Behere GT, Roy SS, Dutta SK, Ngachan SV (2014) Identification and characterization of a distinct banana bunchy top virus isolate of Pacific-Indian Oceans group from North-East India. Virus Res 183:41-49

5. Bashir R, Javed F, Ahmed R, Mansoor S (2012) Use of rolling circle amplification for the identification of unknown components of banana bunchy top virus from Pakistan. Pakistan Journal of Life and Social Sciences 10:91-97

6. Bell KE, Dale JL, Ha CV, Vu MT, Revill PA (2002) Characterisation of Rep-encoding components associated with banana bunchy top nanovirus in Vietnam. Arch Virol 147:695-707

7. Burns TM, Harding R. M., Dale JL (1995) The genome organisation of banana bunchy top virus: analysis of six ssDNA components. J Gen Virol 76:1471-1482

8. Fu HC, Hu JM, Hung TH, Su HJ, Yeh HH (2009) Unusual events involved in banana bunchy top virus strain evolution. Phytopathology 99:812-822

9. Furuya N, Somowiyarjo S, Natsuaki K (2004) Virus detection from local banana cultivars and the first molecular characterization of Banana bunchy top virus in Indonesia. Journal of Agricultural Sciencetokyo University of Agriculture 49:75-81

10. Furuya N, Kawano S, Natsuaki KT (2005) Characterization and genetic status of banana bunchy top virus isolated from Okinawa, Japan. J Gen Plant Pathol 71:68-73

11. Furuya N, Dizon TO, Natsuaki KT (2006) Molecular Characterization of Banana bunchy top virus and Cucumber mosaic virus from Abaca (Musa textilis Nee). Journal of Agricultural Sciencetokyo University of Agriculture 51:92-101

12. He ZF, Li HP, Xiao HG, Fan HZ (2000) Cloning and sequencing of DNA component 1 of two BBTV strains. Chih Wu Ping Li Hsueh Pao 30:364-369

13. He ZF, Li HP, Xiao HG, Fan HZ (2001) Cloning and sequencing of DNA component 3 of two BBTV strains. Nongye Shengwu Jisu Xuebao 9:145-148

14. He ZF, Li HP, Xiao HG, Fan HZ (2001) Cloning and sequencing of DNA component 6 of two BBTV strains. Hua Nan Nung Yeh Ta Hsueh Hsueh Pao 22:33-36

15. Hu JM, Fu HC, Lin CH, Su HJ, Yeh HH (2007) Reassortment and concerted evolution in Banana bunchy top virus genomes. J Virol 81:1746-1761

16. Hyder MZ, Raza SQ, Hameed S, Khalid S, Saqlan Naqvi SM (2007) Phylogenetic relationship of TJ1 isolate of Banana bunchy top virus from Pakistan by DNA-R sequence analysis. Can J Plant Pathol 29:63-68

17. Hyder MZ (2009) Sequencing and Genetic Characterization of major components of Banana Bunchy Top Virus. Department of Biochemistry. Arid Agriculture University, Pakistan

18. Hyder MZ, Shah SH, Hameed S, Naqvi SMS (2011) Evidence of recombination in the Banana bunchy top virus genome. Infect Genet Evol 11:1293-1300

19. Islam MN, Naqvi AR, Jan AT, Haq QMR (2010) Genetic Diversity and Possible Evidence of Recombination among Banana Bunchy Top Virus (BBTV) Isolates. International Research Journal of Microbiology Vol 1:001-012

20. James AP (2011) Viruses of Banana in East Africa. Queensland University of Technology

21. Jun TEZ, Zhi-Xin L (2005) Cloning and sequencing of DNA components of Banana bunchy top virus Hainan isolate. Chinese Journal of Agricultural Biotechnology 2:91-97

22. Karan M, Harding RM, Dale JL (1994) Evidence for two groups of banana bunchy top virus isolates. J Gen Virol 75:3541-3546

23. Kumar P, Hanna R, Alabi O, Soko M, Oben T, Vangu G, Naidu R (2011) Banana bunchy top virus in sub-Saharan Africa: investigations on virus distribution and diversity. Virus Res 159:171

24. La P, Cai WQ, Fang RX (2000) Cloning and sequencing of banana bunchy top virus genome component I. Chinese Journal of Virology 16:158-161

25. Pinili MS, Nyana D, Suastika G, Natsuaki K (2011) Molecular analysis of Banana bunchy top virus first isolated in Bali, Indonesia. J Agric Sci Tokyo Univ Agric 56:125-134

26. Selvarajan R, Sheeba MM, Balasubramanian V, Rajmohan R, Dhevi NL, Sasireka T (2010) Molecular Characterization of Geographically Different Banana bunchy top virus Isolates in India. Indian Journal of Virology 21:110-116

27. Sharman M, Thomas JE, Skabo S, Holton TA (2008) Abaca bunchy top virus, a new member of the genus Babuvirus (family Nanoviridae). Arch Virol 153:135-147

28. Stainton D, Kraberger S, Walters M, Wiltshire EJ, Rosario K, Halafihi M, Lolohea S, Katoa I, Faitua TH, Aholelei W, Taufa L, Thomas JE, Collings DA, Martin DP, Varsani A (2012) Evidence of inter-component recombination, intra-component recombination and reassortment in banana bunchy top virus. J Gen Virol 93:1103-1119

29. Su HJ, Tsao LY, Wu ML, Hung TH (2003) Biological and molecular categorization of strains of Banana bunchy top virus. Journal of Phytopathology 151:290-296

30. Vishnoi R, Raj S, Prasad V (2009) Molecular characterization of an Indian isolate of Banana bunchy top virus based on six genomic DNA components. Virus Genes 38:334-344

31. Wanitchakorn R, Harding RM, Dale JL (2000) Sequence variability in the coat protein gene of two groups of banana bunchy top isolates. Arch Virol 145:593-602

32. Xie WS, Hu JS (1995) Molecular cloning, sequence analysis, and detection of banana bunchy top virus in Hawaii. Phytopathology 85:339-347

33. Yu NT, Zhang YL, Feng TC, Wang JH, Kulye M, Yang WJ, Lin ZS, Xiong Z, Liu ZX (2012) Cloning and sequence analysis of two banana bunchy top virus genomes in Hainan. Virus Genes 44:488-494
