# Supplementary material for: The global distribution of Banana bunchy top virus reveals little evidence for frequent recent, human-mediated long distance dispersal events
Source: Virus Evol. 2015 Sep 10;1(1):vev009. doi: 10.1093/ve/vev009 (PMC5014477; doi:10.1093/ve/vev009)
Supplement: Supplementary Table S1 [file Supp_Table_2.docx]

Supplementary Table 2

| **Event Number** | **Reassorted component** | **Recombinant Sequence(s)** | **Sequence(s) used to infer minor parent(s)** | **Sequence(s) used to infer major parent(s)** | **Detection Methods** | **p-value** |
| --- | --- | --- | --- | --- | --- | --- |
| 1 | U3 | 627-TW-1996-D8 | 24tw-TW  48pk-PK-2007  49pk-PK-2007  60pk-PK  64in-IN-2009  9-150510-EG-2010  B2820-AU-2011  B2823-AU-2011  B2827-AU-2011  B2834-AU-2011  TOS19-TO-2010  TOS85-TO-2010  TOS88-TO-2010  19rw-RW-2009-C2  20rw-RW-2009-C2  26pk-PK-2004-C2  3in-IN-2007-C2  33in-IN-2002-C2  51in-IN-C2  526-BI-1992-C2  549-BI-1995-C2  65lk-LK-2010-C2  BU1-CD-2012-C2  BU10-CD-2012-C2  BU11-CD-2012-C2  BU17-CD-2012-C2  BU20-CD-2012-C2  BU9-CD-2012-C2  Q524-1-IN-C2  Q524-3-IN-C2  All C1 except 5  *35to-TO-2010-C1*  *36to-TO-2010-C1*  *38to-TO-2010-C*  *TOS56-TO-2010-C1*  *TOS83-TO-2010-C1* | 625I-TW-1995  625-TW-1996  523-6B-IN-1991-D8  All D2 1/1  All D4 1/1  All D5 16/16  All D6 1/1  All D7 1/1 | RGMCS**T** | **3.51x10^-135^** |
| 2 | S | Q623-TW-1996-D7 | 547-BI-1995  602-AU-1996  9-150510-EG-2010  B2820-AU-2011  B2828-AU-2011  B2830-AU-2011  B2834-AU-2011  TOS14-TO-2010  TOS19-TO-2010  TOS43-TO-2010  TOS55-TO-2010  TOS58-TO-2010  TOS59-TO-2010  TOS62-TO-2010  TOS63B-TO-2010  TOS64-TO-2010  TOS68-TO-2010  TOS70-TO-2010  TOS71-TO-2010  TOS78-TO-2010  TOS82-TO-2010  TOS85-TO-2010  TOS87-TO-2010  TOS88-TO-2010  TOS89-TO-2010  All B1 1/1  All C1 40/40  All C2 33/33  All C3 except 1  *B2819-AU-2011-C3* | All D2 1/1  All D4 1/1  All D6 1/1  All D5 except  *25tw-TW-D5* | GMCS**T** | **8.44x10^-96^** |
| 3 | N | 8-150510-EG-2010-A1 | 1pk-PK-2004  1429B-AU  B2818-AU-2011  B2823-AU-2011  B2833-AU-2011  B2844-AU-2011  B2846-AU-2011  KP8-AU-1989  TOS4-TO-2010  TOS40-TO-2010  TOS49-TO-2010  TOS57-TO-2010  TOS72-TO-2010  All B1 1/1  All C1 40/40  All C2 33/33  All C3 22/22 | 625-TW-1996  768-PH-1995  Q568-3-ID-1995  All D2 1/1  All D3 1/1  All D4 1/1  All D5 16/16  All D6 1/1  All D7 1/1  All D8 2/2  All E1 1/1 | R**G**MCST | **6.07x10^-70^** |
| 4 | U3 | Q529-6-CN-1990 | 9-150510-EG-2010  TOS19-TO-2010  TOS40-TO-2010  TOS49-TO-2010  TOS72-TO-2010  TOS85-TO-2010  TOS88-TO-2010  B2818-AU-2011  B2820-AU-2011  B2833-AU-2011  B2834-AU-2011  34eg-EG-1997  Q524-3-IN-C2  3in-IN-2007-C2  33in-IN-2002-C2  51in-IN-C2  65lk-LK-2010-C2  BU10-CD-2012-C2  BU11-CD-2012-C2  BU17-CD-2012-C2  Q524-1-IN-C2  All C3 22/22  All C1 except 5  *35to-TO-2010-C1*  *36to-TO-2010-C1*  *38to-TO-2010-C1*  *TOS56-TO-2010-C1*  *TOS83-TO-2010-C1* | Q529-4-CN-1990-E1 | RGMCS**T** | **1.17x10^-86^** |
| 5 | C | 24tw-TW | 1pk-PK-2004  1429B-AU  9-150510-EG-2010  B2818-AU-2011  B2820-AU-2011  B2828-AU-2011  B2832-AU-2011  B2833-AU-2011  B2846-AU-2011  KP7-AU-1989  KP8-AU-1989  TOS15-TO-2010  TOS4-TO-2010  TOS40-TO-2010  TOS49-TO-2010  TOS57-TO-2010  TOS62-TO-2010  TOS72-TO-2010  TOS76-TO-2010  All B1 1/1  All C1 40/40  All C2 33/33  All C3 22/22 | 25tw-TW-D5  765tw-TW-1996-D5  MS7-PH-2008-D5  Q1160-TW-1995-D5  Q624-TW-1996-D5  All A1 1/1  All D6 1/1  All D7 1/1 | RGMCS**T** | **1.49x10^-69^** |
| 6 | M | TOS88-TO-2010 | 1pk-PK-2004  22in-IN  547-BI-1995  527-US-1992-C1  KP9-US-1990-C1  Q279-WS-1989-C1  All C2 except 1  *736-4-IN-1997-C2* | TOS19-TO-2010  TOS5-TO-2010  TOS55-TO-2010  TOS63B-TO-2010  TOS64-TO-2010  TOS67-TO-2010  TOS68-TO-2010  TOS71-TO-2010  TOS72-TO-2010  TOS78-TO-2010  TOS80-TO-2010  TOS82-TO-2010  TOS85-TO-2010  TOS87-TO-2010  TOS89-TO-2010  37to-TO-2010-C1  39to-TO-2010-C1  40to-TO-2010-C1  41to-TO-2010-C1  536-TO-1993-C1  KP4-TO-1990-C1  Q276-TO-1989-C1  Q277-TO-1989-C1  Q278-TO-1989-C1  Q570-TO-1990-C1  TOS16-TO-2010-C1  TOS2-TO-2010-C1  TOS20-TO-2010-C1  TOS21-TO-2010-C1  TOS22-TO-2010-C1  TOS25-TO-2010-C1  TOS29-TO-2010-C1  TOS42-TO-2010-C1  TOS60-TO-2010-C1  TOS63A-TO-2010-C1  TOS65-TO-2010-C1  TOS91-TO-2010-C1  TOS93-TO-2010-C1 | RGMCS**T** | **3.38x10^-25^** |
| 7 | N | 1429A-AU | 602-AU-1996  B2823-AU-2011  B2830-AU-2011  1900A-AU-2006-C3  B2817-AU-2011-C3  B2819-AU-2011-C3  B2822-AU-2011-C3  KP14-AU-2009-C3  KP6-AU-2011-C3 | TOS14-TO-2010  TOS55-TO-2010  TOS58-TO-2010  TOS61-TO-2010  TOS68-TO-2010  TOS78-TO-2010  TOS82-TO-2010  TOS87-TO-2010  All C1 except 9  *44to-TO-2010-C1*  *45to-TO-2010-C1*  *46to-TO-2010-C*  *Q276-TO-1989-C1*  *Q277-TO-1989-C1*  *Q279-WS-1989-C1*  *TOS60-TO-2010-C1*  *TOS65-TO-2010-C1*  *TOS83-TO-2010-C1* | RGMCS**T** | **1.55x10^-25^** |
| 8 | S | B2819-AU-2011-C3 | Unknown | 602-AU-1996  737-AU-1997  B2830-AU-2011  B2834-AU-2011  TOS19-TO-2010  TOS43-TO-2010  TOS55-TO-2010  TOS58-TO-2010  TOS59-TO-2010  TOS62-TO-2010  TOS63B-TO-2010  TOS68-TO-2010  TOS70-TO-2010  TOS71-TO-2010  TOS78-TO-2010  TOS82-TO-2010  TOS85-TO-2010  TOS87-TO-2010  TOS89-TO-2010  35to-TO-2010-C1  36to-TO-2010-C1  37to-TO-2010-C1  38to-TO-2010-C1  39to-TO-2010-C1  40to-TO-2010-C1  41to-TO-2010-C1  536-TO-1993-C1  KP4-TO-1990-C1  Q276-TO-1989-C1  Q277-TO-1989-C1  Q278-TO-1989-C1  Q570-TO-1990-C1  TOS16-TO-2010-C1  TOS2-TO-2010-C1  TOS20-TO-2010-C1  TOS21-TO-2010-C1  TOS22-TO-2010-C1  TOS25-TO-2010-C1  TOS29-TO-2010-C1  TOS42-TO-2010-C1  TOS56-TO-2010-C1  TOS60-TO-2010-C1  TOS63A-TO-2010-C1  TOS65-TO-2010-C1  TOS83-TO-2010-C1  TOS91-TO-2010-C1  TOS93-TO-2010-C1  736-4-IN-1997-C2  All C3 except 6  *B2817-AU-2011-C3*  *B2819-AU-2011-C3*  *B2824-AU-2011-C3*  *B2829-AU-2011-C3*  *B2845-AU-2011-C3*  *KP15-AU-2009-C3* | R**G**MCST | **2.83x10^-24^** |
| 9 | U3 | 523-6B-IN-1991-D8 | 22in-IN  34eg-EG-1997  6us-US  602-AU-1996  64in-IN-2009  9-150510-EG-2010  B2818-AU-2011  B2820-AU-2011  B2823-AU-2011  B2827-AU-2011  B2830-AU-2011  B2833-AU-2011  B2834-AU-2011  Q524-2-IN  TOS14-TO-2010  TOS19-TO-2010  TOS4-TO-2010  TOS40-TO-2010  TOS43-TO-2010  TOS49-TO-2010  TOS55-TO-2010  TOS62-TO-2010  TOS63B-TO-2010  TOS64-TO-2010  TOS67-TO-2010  TOS68-TO-2010  TOS72-TO-2010  TOS78-TO-2010  TOS80-TO-2010  TOS82-TO-2010  TOS85-TO-2010  TOS87-TO-2010  TOS88-TO-2010  TOS89-TO-2010  All B1 1/1  All C1 40/40  19rw-RW-2009-C2  20rw-RW-2009-C2  3in-IN-2007-C2  33in-IN-2002-C2  51in-IN-C2  65lk-LK-2010-C2  BU1-CD-2012-C2  BU10-CD-2012-C2  BU11-CD-2012-C2  BU12-CD-2012-C2  BU17-CD-2012-C2  BU20-CD-2012-C2  BU6-CD-2012-C2  BU9-CD-2012-C2  Q524-1-IN-C2  Q524-3-IN-C2  All C3 22/22 | 625-TW-1996  MP1-TW-1996  MS14-PH-2008  Q568-3-ID-1995  All D2 1/1  All D4 1/1  All D5 16/16  All D6 1/1  All D7 1/1 | RGMCS**T** | **7.04x10^-78^** |
| 10 | U3 | 24tw-TW | 34eg-EG-1997  9-150510-EG-2010  B2818-AU-2011  B2820-AU-2011  B2833-AU-2011  B2834-AU-2011  TOS19-TO-2010  TOS72-TO-2010  TOS85-TO-2010  All B1 1/1  37to-TO-2010-C1  39to-TO-2010-C1  40to-TO-2010-C1  41to-TO-2010-C1  536-TO-1993-C1  KP4-TO-1990-C1  Q276-TO-1989-C1  Q277-TO-1989-C1  Q278-TO-1989-C1  Q570-TO-1990-C1  TOS16-TO-2010-C1  TOS2-TO-2010-C1  TOS20-TO-2010-C1  TOS21-TO-2010-C1  TOS22-TO-2010-C1  TOS25-TO-2010-C1  TOS29-TO-2010-C1  TOS42-TO-2010-C1  TOS60-TO-2010-C1  TOS63A-TO-2010-C1  TOS65-TO-2010-C1  TOS91-TO-2010-C1  TOS93-TO-2010-C1  19rw-RW-2009-C2  20rw-RW-2009-C2  3in-IN-2007-C2  33in-IN-2002-C2  51in-IN-C2  65lk-LK-2010-C2  BU1-CD-2012-C2  BU10-CD-2012-C2  BU11-CD-2012-C2  BU12-CD-2012-C2  BU13-CD-2012-C2  BU17-CD-2012-C2  BU20-CD-2012-C2  BU6-CD-2012-C2  BU7-CD-2012-C2  BU9-CD-2012-C2  Q524-1-IN-C2  Q524-3-IN-C2  All C3 22/22 | Q568-3-ID-1995  All D2 1/1  All D4 1/1  All D5 16/16  All D6 1/1  All D7 1/1 | RGMCS**T** | **2.46x10^-81^** |
| 11 | N | 625I-TW-1995 | TOS19-TO-2010  TOS5-TO-2010  TOS85-TO-2010  TOS88-TO-2010  All C1 40/40 | 768-PH-1995  Q529-1-CN-1990  625-TW-1996  All D1 1/1  All D2 1/1  All D3 1/1  All D4 1/1  All D6 1/1  All D8 2/2  All E1 1/1  All D5 except 3  *522B-PH-1991-D5*  *571-1-PH-1993-D5*  *571-2-PH-1993-D5* | RGMCS**T** | **1.53x10^-55^** |
| 12 | M | 736-4-IN-1997-C2 | TOS15-TO-2010  TOS19-TO-2010  TOS4-TO-2010  TOS43-TO-2010  TOS5-TO-2010  TOS55-TO-2010  TOS62-TO-2010  TOS63B-TO-2010  TOS68-TO-2010  TOS78-TO-2010  TOS82-TO-2010  TOS85-TO-2010  TOS87-TO-2010  TOS89-TO-2010  35to-TO-2010-C1  36to-TO-2010-C1  37to-TO-2010-C1  38to-TO-2010-C1  39to-TO-2010-C1  40to-TO-2010-C1  536-TO-1993-C1  KP4-TO-1990-C1  Q276-TO-1989-C1  Q277-TO-1989-C1  Q278-TO-1989-C1  Q570-TO-1990-C1  TOS16-TO-2010-C1  TOS2-TO-2010-C1  TOS20-TO-2010-C1  TOS21-TO-2010-C1  TOS22-TO-2010-C1  TOS25-TO-2010-C1  TOS29-TO-2010-C1  TOS42-TO-2010-C1  TOS56-TO-2010-C1  TOS60-TO-2010-C1  TOS63A-TO-2010-C1  TOS65-TO-2010-C1  TOS83-TO-2010-C1  TOS91-TO-2010-C1 | 547-BI-1995  All C2 except 2  *3in-IN-2007-C2*  *736-4-IN-1997-C2* | RGMCS**T** | **9.34x10^-23^** |
| 13 | M | B2833-AU-2011 | 1429B-AU  KP8-AU-1989  BU14-CD-2012-C2 | TOS4-TO-2010  TOS58-TO-2010  TOS63B-TO-2010  TOS64-TO-2010  TOS78-TO-2010  TOS82-TO-2010  TOS87-TO-2010  TOS22-TO-2010-C1  35to-TO-2010-C1  37to-TO-2010-C1  38to-TO-2010-C1  39to-TO-2010-C1  40to-TO-2010-C1  536-TO-1993-C1  KP4-TO-1990-C1  Q570-TO-1990-C1  TOS16-TO-2010-C1  TOS2-TO-2010-C1  TOS20-TO-2010-C1  TOS21-TO-2010-C1  TOS25-TO-2010-C1  TOS29-TO-2010-C1  TOS42-TO-2010-C1  TOS56-TO-2010-C1  TOS60-TO-2010-C1  TOS63A-TO-2010-C1  TOS83-TO-2010-C1  TOS91-TO-2010-C1  TOS93-TO-2010-C1 | RM**S** | **3.33x10^-22^** |
| 14 | R | 62cn-CN-D2 | 21cn-CN-D1 | 768-PH-1995  Q568-3-ID-1995  625-TW-1996  All D4 1/1  All D6 1/1  All D7 1/1  All D8 2/2  All D5 except 3  *522B-PH-1991-D5*  *571-1-PH-1993-D5*  *571-2-PH-1993-D5* | RGMC**S**T | **1.59x10^-15^** |
| 15 | N | 62cn-CN-D2 | 21cn-CN-D1 | 768-PH-1995  625-TW-1996  All D3 1/1  All D4 1/1  All D8 2/2  All D5 except 3  *522B-PH-1991-D5*  *571-1-PH-1993-D5*  *571-2-PH-1993-D5* | GMS**T** | **3.85x10^-27^** |
| 16 | U3 | 9-150510-EG-2010  527-US-1992-C1^#^  KP9-US-1990-C1^#^  Q279-WS-1989-C1  Q281-WS-1989-C1 | TOS19-TO-2010  TOS4-TO-2010  TOS62-TO-2010  TOS63B-TO-2010  TOS85-TO-2010  All C1 except 8  *43to-TO-2010-C1*  *44to-TO-2010-C1*  *46to-TO-2010-C1*  *527-US-1992-C1*  *KP9-US-1990-C1*  *Q279-WS-1989-C1*  *Q281-WS-1989-C1*  *TOS83-TO-2010-C1* | All C2 except 8  *26pk-PK-2004-C2*  *3in-IN-2007-C2*  *33in-IN-2002-C2*  *51in-IN-C2*  *65lk-LK-2010-C2*  *736-4-IN-1997-C2*  *KP5-LK-2003-C2*  *Q524-1-IN-C2* | RGM**S**T | **7.39x10^-14^** |
| 17 | S | 8-150510-EG-2010-A1 | 1pk-PK-2004  9-150510-EG-2010  TOS15-TO-2010  TOS19-TO-2010  TOS5-TO-2010  TOS62-TO-2010  TOS85-TO-2010  All B1 1/1  All C1 40/40  All C2 except 1  *736-4-IN-1997-C2* | 5tw-TW  768-PH-1995  All D4 1/1  All D5 16/16  All D6 1/1  All D8 2/2 | RGBMS**T** | **1.34x10^-86^** |
| 18 | M | TOS14-TO-2010 | 1pk-PK-2004  1429B-AU  22in-IN  547-BI-1995  KP9-US-1990-C1  Q279-WS-1989-C1  All C2 except 1  *736-4-IN-1997-C2* | TOS19-TO-2010  TOS4-TO-2010  TOS55-TO-2010  TOS58-TO-2010  TOS62-TO-2010  TOS63B-TO-2010  TOS64-TO-2010  TOS67-TO-2010  TOS68-TO-2010  TOS72-TO-2010  TOS78-TO-2010  TOS80-TO-2010  TOS82-TO-2010  TOS85-TO-2010  TOS87-TO-2010  TOS89-TO-2010  35to-TO-2010-C1  36to-TO-2010-C1  37to-TO-2010-C1  38to-TO-2010-C1  39to-TO-2010-C1  40to-TO-2010-C1  41to-TO-2010-C1  536-TO-1993-C1  KP4-TO-1990-C1  Q276-TO-1989-C1  Q277-TO-1989-C1  Q278-TO-1989-C1  Q570-TO-1990-C1  TOS16-TO-2010-C1  TOS2-TO-2010-C1  TOS20-TO-2010-C1  TOS21-TO-2010-C1  TOS22-TO-2010-C1  TOS25-TO-2010-C1  TOS29-TO-2010-C1  TOS42-TO-2010-C1  TOS56-TO-2010-C1  TOS60-TO-2010-C1  TOS63A-TO-2010-C1  TOS65-TO-2010-C1  TOS83-TO-2010-C1  TOS91-TO-2010-C1 | RGBMCS**T** | **3.37x10^-19^** |
| 19 | S | TOS71-TO-2010 | 602-AU-1996  B2820-AU-2011  B2823-AU-2011  B2828-AU-2011  B2830-AU-2011  B2834-AU-2011  19rw-RW-2009-C2  20rw-RW-2009-C2  3in-IN-2007-C2  47mw-MW-2008-C2  526-BI-1992-C2  549-BI-1995-C2  550-CG-1995-C2  BU1-CD-2012-C2  BU11-CD-2012-C2  BU12-CD-2012-C2  BU13-CD-2012-C2  BU14-CD-2012-C2  BU15-CD-2012-C2  BU16-CD-2012-C2  BU17-CD-2012-C2  BU18-CD-2012-C2  BU19-CD-2012-C2  BU2-CD-2012-C2  BU20-CD-2012-C2  BU6-CD-2012-C2  BU7-CD-2012-C2  Q553-LK-1995-C2  All C3 except 1  [B2819-AU-2011-C3] | TOS14-TO-2010  TOS19-TO-2010  TOS55-TO-2010  TOS61-TO-2010  TOS62-TO-2010  TOS63B-TO-2010  TOS64-TO-2010  TOS68-TO-2010  TOS78-TO-2010  TOS82-TO-2010  TOS85-TO-2010  TOS87-TO-2010  All C1 except 8  *43to-TO-2010-C1*  *44to-TO-2010-C1*  *527-US-1992-C1*  *KP9-US-1990-C1*  *Q279-WS-1989-C1*  *Q281-WS-1989-C1*  *TOS90-TO-2010-C1*  *TOS93-TO-2010-C1* | RGB**T** | **1.10x10^-11^** |
| 20 | U3 | 44to-TO-2010-C1  45to-TO-2010-C1  46to-TO-2010-C1 | B2818-AU-2011  B2823-AU-2011  B2830-AU-2011  B2833-AU-2011  B2834-AU-2011  All C3 except 2  *1900B-AU-2006-C3*  *KP17-AU-2010-C3* | TOS93-TO-2010-C1 | RGBM**S**T | **2.76x10^-22^** |
| 21 | C | KP9-US-1990-C1  527-US-1992-C1  Q279-WS-1989-C1  Q281-WS-1989-C1 | TOS19-TO-2010  TOS4-TO-2010  TOS63B-TO-2010  TOS64-TO-2010  TOS67-TO-2010  TOS68-TO-2010  TOS80-TO-2010  TOS89-TO-2010  35to-TO-2010-C1  36to-TO-2010-C1  37to-TO-2010-C1  41to-TO-2010-C1  KP4-TO-1990-C1  Q570-TO-1990-C1  TOS2-TO-2010-C1  TOS20-TO-2010-C1  TOS21-TO-2010-C1  TOS22-TO-2010-C1  TOS25-TO-2010-C1  TOS29-TO-2010-C1  TOS42-TO-2010-C1  TOS56-TO-2010-C1  TOS60-TO-2010-C1  TOS65-TO-2010-C1  TOS83-TO-2010-C1  TOS93-TO-2010-C1 | 22in-IN  33in-IN-2002-C2  47mw-MW-2008-C2  51in-IN-C2  526-BI-1992-C2  548-BI-1995-C2  550-CG-1995-C2  65lk-LK-2010-C2  BU1-CD-2012-C2  BU10-CD-2012-C2  BU12-CD-2012-C2  BU13-CD-2012-C2  BU15-CD-2012-C2  BU16-CD-2012-C2  BU17-CD-2012-C2  BU18-CD-2012-C2  BU19-CD-2012-C2  BU6-CD-2012-C2  BU7-CD-2012-C2  KP5-LK-2003-C2  Q553-LK-1995-C2  20rw-RW-2009-C2  19rw-RW-2009-C2 | RBMS**T** | **8.82x10^-35^** |
| 22 | U3 | Q529-4-CN-1990-E1  Q529-2-CN-1990 | TOS14-TO-2010  TOS19-TO-2010  TOS88-TO-2010  TOS91-TO-2010-C1  39to-TO-2010-C1  40to-TO-2010-C1  42to-TO-2010-C1  Q276-TO-1989-C1  Q277-TO-1989-C1  TOS20-TO-2010-C1  TOS21-TO-2010-C1  TOS39-TO-2010-C1  TOS48-TO-2010-C1  TOS60-TO-2010-C1  TOS65-TO-2010-C1  TOS90-TO-2010-C1  TOS93-TO-2010-C1  33in-IN-2002-C2  51in-IN-C2  1900A-AU-2006-C3  1900B-AU-2006-C3  KP17-AU-2010-C3  KP18-AU-2010-C3 | 625I-TW-1995  625-TW-1996  All D1 1/1  All D2 1/1  All D4 1/1  All D6 1/1  All D5 except 5  *522A-PH-1991-D5*  *571-2-PH-1993-D5*  *MS17-PH-2008-D5*  *MS6-PH-2008-D5*  *MS7-PH-2008-D5* | **M**CS | **4.78x10^-05^** |
| 23 | U3 | B2818-AU-2011  B2823-AU-2011  B2827-AU-2011  B2833-AU-2011  B2834-AU-2011  All C3 22/22 | TOS19-TO-2010  TOS40-TO-2010  TOS62-TO-2010  TOS72-TO-2010  38to-TO-2010-C1  39to-TO-2010-C1  40to-TO-2010-C1  42to-TO-2010-C1  536-TO-1993-C1  KP4-TO-1990-C1  Q277-TO-1989-C1  Q278-TO-1989-C1  Q570-TO-1990-C1  TOS16-TO-2010-C1  TOS20-TO-2010-C1  TOS21-TO-2010-C1  TOS22-TO-2010-C1  TOS25-TO-2010-C1  TOS29-TO-2010-C1  TOS39-TO-2010-C1  TOS42-TO-2010-C1  TOS48-TO-2010-C1  TOS56-TO-2010-C1  TOS60-TO-2010-C1  TOS63A-TO-2010-C1  TOS65-TO-2010-C1  TOS91-TO-2010-C1 | 27-IN-2006  64in-IN-2009  526-BI-1992-C2  548-BI-1995-C2  549-BI-1995-C2  736-4-IN-1997-C2  BU10-CD-2012-C2  BU11-CD-2012-C2  BU14-CD-2012-C2  BU16-CD-2012-C2  BU17-CD-2012-C2  BU2-CD-2012-C2  BU7-CD-2012-C2  BU9-CD-2012-C2  Q524-1-IN-C2  Q524-3-IN-C2  Q553-LK-1995-C2 | RGBMC**S** | **3.03x10^-14^** |
| 24 | N | 1429B-AU KP8-AU-1989 | 602-AU-1996  737-AU-1997  B2818-AU-2011  B2826-AU-2011  B2830-AU-2011  B2834-AU-2011  B2846-AU-2011  All C3 22/22 | Unknown | RGBMC**T** | **1.77x10^-17^** |
| 25 | S | 523-6A-IN-1991 | 29mm-MM  30mm-MM  31mm-MM  64in-IN-2009  9-150510-EG-2010  B2820-AU-2011  B2826-AU-2011  B2828-AU-2011  B2832-AU-2011  B2834-AU-2011  B2846-AU-2011  TOS15-TO-2010  TOS19-TO-2010  TOS5-TO-2010  TOS62-TO-2010  TOS85-TO-2010  All B1 1/1  All C1 40/40  All C2 33/33  All C3 except 1  *B2819-AU-2011-C3* | 10ph-PH  13ph-PH  14ph-PH  15ph-PH  16id-ID  17id-ID  18id-ID  5tw-TW  7jp-JP  768-PH-1995  8jp-JP  9jp-JP  All D1 1/1  All D4 1/1  All D5 16/16  All D6 1/1  All D8 2/2 | RGBMCS**T** | **2.15x10^-64^** |
| 26 | M | B2834-AU-2011 | TOS15-TO-2010  TOS19-TO-2010  TOS4-TO-2010  TOS5-TO-2010  TOS55-TO-2010  TOS57-TO-2010  TOS58-TO-2010  TOS62-TO-2010  TOS63B-TO-2010  TOS64-TO-2010  TOS68-TO-2010  TOS72-TO-2010  TOS78-TO-2010  TOS82-TO-2010  TOS85-TO-2010  TOS89-TO-2010  35to-TO-2010-C1  36to-TO-2010-C1  37to-TO-2010-C1  38to-TO-2010-C1  39to-TO-2010-C1  40to-TO-2010-C1  41to-TO-2010-C1  536-TO-1993-C1  KP4-TO-1990-C1  Q276-TO-1989-C1  Q277-TO-1989-C1  Q278-TO-1989-C1  Q570-TO-1990-C1  TOS16-TO-2010-C1  TOS2-TO-2010-C1  TOS20-TO-2010-C1  TOS21-TO-2010-C1  TOS22-TO-2010-C1  TOS25-TO-2010-C1  TOS29-TO-2010-C1  TOS42-TO-2010-C1  TOS56-TO-2010-C1  TOS60-TO-2010-C1  TOS63A-TO-2010-C1  TOS65-TO-2010-C1  TOS83-TO-2010-C1  TOS91-TO-2010-C1  TOS93-TO-2010-C1 | 1pk-PK-2004  9-150510-EG-2010  Q281-WS-1989-C1  BU17-CD-2012-C2  All C2 except 2  *26pk-PK-2004-C2*  *736-4-IN-1997-C2* | RGBMC**S**T | **8.43x10^-20^** |
| 27 | R | KP8-AU-1989  1429B-AU  KP7-AU-1989 | B2818-AU-2011  B2820-AU-2011  B2826-AU-2011  B2828-AU-2011  B2832-AU-2011  B2834-AU-2011  B2846-AU-2011  All C3 22/22 | Unknown | RBMC**S**T | **2.13x10^-21^** |
| 28 | M | TOS40-TO-2010  TOS49-TO-2010  42to-TO-2010-C1  43to-TO-2010-C1  44to-TO-2010-C1  45to-TO-2010-C1  46to-TO-2010-C1  TOS39-TO-2010-C1  TOS48-TO-2010-C1  TOS90-TO-2010-C1 | 547-BI-1995  All C2 except 3  *26pk-PK-2004-C2*  *736-4-IN-1997-C2*  *BU17-CD-2012-C2* | TOS15-TO-2010  TOS19-TO-2010  TOS43-TO-2010  TOS55-TO-2010  TOS62-TO-2010  TOS63B-TO-2010  TOS64-TO-2010  TOS68-TO-2010  TOS78-TO-2010  TOS82-TO-2010  TOS85-TO-2010  TOS87-TO-2010  TOS89-TO-2010  37to-TO-2010-C1  35to-TO-2010-C1  36to-TO-2010-C1  38to-TO-2010-C1  39to-TO-2010-C1  40to-TO-2010-C1  536-TO-1993-C1  KP4-TO-1990-C1  Q276-TO-1989-C1  Q277-TO-1989-C1  Q278-TO-1989-C1  Q570-TO-1990-C1  TOS16-TO-2010-C1  TOS2-TO-2010-C1  TOS20-TO-2010-C1  TOS21-TO-2010-C1  TOS22-TO-2010-C1  TOS25-TO-2010-C1  TOS29-TO-2010-C1  TOS42-TO-2010-C1  TOS56-TO-2010-C1  TOS60-TO-2010-C1  TOS65-TO-2010-C1  TOS83-TO-2010-C1  TOS91-TO-2010-C1  TOS93-TO-2010-C1 | RGBMCS**T** | **6.63x10^-18^** |
| 29 | N | 527-US-1992-C1  KP9-US-1990-C1  Q279-WS-1989-C1  Q281-WS-1989-C1 | TOS19-TO-2010  TOS4-TO-2010  TOS5-TO-2010  TOS55-TO-2010  TOS57-TO-2010  TOS63B-TO-2010  TOS64-TO-2010  TOS67-TO-2010  TOS68-TO-2010  TOS71-TO-2010  TOS72-TO-2010  TOS80-TO-2010  TOS82-TO-2010  TOS85-TO-2010  TOS87-TO-2010  TOS89-TO-2010  35to-TO-2010-C1  38to-TO-2010-C1  39to-TO-2010-C1  536-TO-1993-C1  KP4-TO-1990-C1  Q276-TO-1989-C1  Q277-TO-1989-C1  Q278-TO-1989-C1  Q570-TO-1990-C1  TOS16-TO-2010-C1  TOS2-TO-2010-C1  TOS20-TO-2010-C1  TOS21-TO-2010-C1  TOS22-TO-2010-C1  TOS25-TO-2010-C1  TOS29-TO-2010-C1  TOS56-TO-2010-C1  TOS60-TO-2010-C1  TOS63A-TO-2010-C1  TOS65-TO-2010-C1  TOS83-TO-2010-C1  TOS91-TO-2010-C1  TOS93-TO-2010-C1 | 1pk-PK-2004  22in-IN  547-BI-1995  All C2 except 1  *736-4-IN-1997-C2* | GBMCS**T** | **8.44x10^-09^** |
| 30 | N | 63cn-CN-D3 | 23cn-CN-2008-D4 | Unknown | GB**S** | **5.89x10^-05^** |
| 31 | S | 24tw-TW | 1pk-PK-2004  32fj-FJ  64in-IN-2009  9-150510-EG-2010  B2820-AU-2011  B2826-AU-2011  B2828-AU-2011  B2832-AU-2011  B2846-AU-2011  TOS15-TO-2010  TOS19-TO-2010  TOS34-TO-2010  TOS45to-TO-2010  TOS46to-TO-2010  TOS5-TO-2010  TOS62-TO-2010  TOS85-TO-2010  TOS88-TO-2010  All B1 1/1  All C1 except 8  *42to-TO-2010-C1*  *43to-TO-2010-C1*  *44to-TO-2010-C1*  *45to-TO-2010-C1*  *46to-TO-2010-C1*  *TOS39-TO-2010-C1*  *TOS48-TO-2010-C1*  *TOS90-TO-2010-C1*  All C2 except 1  *33in-IN-2002-C2*  All C3 except 1  *B2819-AU-2011-C3* | 14ph-PH  15ph-PH  16id-ID  17id-ID  18id-ID  5tw-TW  625-TW-1996  768-PH-1995  All D4 1/1  All D5 16/16  All D6 1/1  All D8 2/2  All E1 1/1 | RGBMS**T** | **8.39x10^-66^** |
| 32 | U3 | 63cn-CN-D3 | 21cn-CN-D1 | MS14-PH-2008  All D2 1/1  All D4 1/1  All D5 except 4  *522A-PH-1991-D5*  *522B-PH-1991-D5*  *765tw-TW-1996-D5*  *Q624-TW-1996-D5* | RGBMCS**T** | **2.47x10^-21^** |
| 33 | M | B2818-AU-2011^#^  B2820-AU-2011  B2828-AU-2011^#^  B2846-AU-2011  All C3 22/22 | TOS19-TO-2010  TOS5-TO-2010  TOS55-TO-2010  TOS57-TO-2010  TOS58-TO-2010  TOS64-TO-2010  TOS68-TO-2010  TOS72-TO-2010  TOS78-TO-2010  TOS82-TO-2010  TOS85-TO-2010  TOS89-TO-2010  35to-TO-2010-C1  36to-TO-2010-C1  37to-TO-2010-C1  38to-TO-2010-C1  39to-TO-2010-C1  40to-TO-2010-C1  41to-TO-2010-C1  536-TO-1993-C1  KP4-TO-1990-C1  Q276-TO-1989-C1  Q277-TO-1989-C1  Q278-TO-1989-C1  Q570-TO-1990-C1  TOS16-TO-2010-C1  TOS2-TO-2010-C1  TOS20-TO-2010-C1  TOS21-TO-2010-C1  TOS22-TO-2010-C1  TOS42-TO-2010-C1  TOS56-TO-2010-C1  TOS60-TO-2010-C1  TOS63A-TO-2010-C1  TOS65-TO-2010-C1  TOS83-TO-2010-C1  TOS91-TO-2010-C1  TOS93-TO-2010-C1 | 547-BI-1995  All C2 except 2  *26pk-PK-2004-C2*  *736-4-IN-1997-C2* | RGBMC**S**T | **6.87x10^-20^** |
| 34 | S | 1429A-AU | TOS63A-TO-2010-C1 | 33in-IN-2002-C2 | RM**T** | **8.15x10^-04^** |
| 35 | U3 | 3in-IN-2007-C2 | Unknown | 65lk-LK-2010-C2 | GB**S** | **3.48x10^-16^** |
| 36 | M | 602-AU-1996  737-AU-1997  B2826-AU-2011[T]  B2830-AU-2011  B2832-AU-2011 | TOS22-TO-2010-C1 | 3in-IN-2007-C2 | RGS**T** | **3.84x10^-12^** |
| 37 | C | MP2-TW-1996-D6 | 520-ID-1995-D5  Q568-1-ID-1995-D5  Q568-3-ID-1995 | Unknown | R**G**BS | **3.95x10^-06^** |
| 38 | C | BU11-CD-2012-C2 | TOS4-TO-2010  TOS58-TO-2010  TOS62-TO-2010  TOS71-TO-2010  TOS83-TO-2010-C1  TOS93-TO-2010-C1  35to-TO-2010-C1  Q278-TO-1989-C1 | Q553-LK-1995-C2 | RB**M**S | **9.33x10^-03^** |
| 39 | C | 9-150510-EG-2010  KP5-LK-2003 | Unknown | 548-BI-1995 | M**S** | **1.51X10^-08^** |
| 40 | U3 | 8-150510-EG-2010 A1 | 571-1-PH-1993-D5 | Unknown | RGMCS**T** | **9.02x10^-8^** |

RDP (R) GENCONV (G), BOOTSCAN (B), MAXCHI (M), CHIMERA (C), SISCAN (S) and 3SEQ (T)

Minor Parent = Parent contributing the smaller fraction of sequence.

Major Parent = Parent contributing the larger fraction of sequence.

Unknown = Only one parent and a recombinant need be in the alignment for a recombination event to be detectable. The sequence listed as unknown was used to infer the existence of a missing parental sequence.

# = Trace evidence was identified for this sequence
