# Supplementary material for: The global distribution of Banana bunchy top virus reveals little evidence for frequent recent, human-mediated long distance dispersal events
Source: Virus Evol. 2015 Sep 10;1(1):vev009. doi: 10.1093/ve/vev009 (PMC5014477; doi:10.1093/ve/vev009)
Supplement: Supplementary Table S1 [file Supp_Table_3.docx]

Supplementary Table 3

| **Recombination Event Number** | **Breakpoints in Alignment** | **Recombinant Sequence(s)** | **Sequence(s) used to infer minor parent(s)** | **Sequence(s) used to infer major parent(s)** | **Detection Methods** | **p-value** |
| --- | --- | --- | --- | --- | --- | --- |
| R1 | 325-732 | AF416476-R-VN  AF416477-R-VN^#^  AF416478-R-VN | Unknown | 10ph-R-PH  11vn-R-VN  13ph-R-PH  14ph-R-PH  15ph-R-PH  16id-R-ID  17id-R-ID  18id-R-ID  24tw-R-TW  523-6A-R-IN-1991  7jp-R-JP  768-R-PH-1995  8jp-R-JP  9jp-R-JP  AB108454-R-JP  AB108455-R-JP  AB108457-R-JP  AB108458-R-JP  AF110266-R-CN  AF246123-R-CN  AF416468-R-TW  AF416469-R-PH  JN003631-R-ID-2010  JN003632-R-ID-2010  JN003633-R-ID-2010  Q568-3-R-ID-1995  627-R-TW-1996-D8  All D5 except 6  *571-2-R-PH-1993-D5*  *MS15-R-PH-2008-D5*  *MS16-R-PH-2008-D5*  *MS18-R-PH-2008-D5*  *Q1160-R-TW-1995-D5*  *Q624-R-TW-1996-D5)* | **M**CT | **1.70x10^-05^** |
| R2 | 1102-46 | MP2-R-TW-1996-D6 | Unknown | 8-150510-R-EG-2010-A1  10ph-R-PH  13ph-R-PH  14ph-R-PH  15ph-R-PH  16id-R-ID  17id-R-ID  18id-R-ID  24tw-R-TW  523-6A-R-IN-1991  625-R-TW-1996  7jp-R-JP  768-R-PH-1995  8jp-R-JP  9jp-R-JP  AB108454-R-JP  AB108455-R-JP  AB108457-R-JP  AB108458-R-JP  AF416468-R-TW  AF416469-R-PH  AF416476-R-VN  JN003631-R-ID-2010  JN003632-R-ID-2010  JN003633-R-ID-2010  Q568-3-R-ID-1995  All D5 16/16  All D7 1/1  All D8 2/2 | **R**GBT | **8.04x10^-05^** |
| R3 | 1088-378 | 5tw-R-TW  625I-R-TW-1995^#^ | Q279-R-WS-1989-C1  Q281-R-WS-1989-C1 | 21cn-R-CN-D1  62cn-R-CN-D2  63cn-R-CN-D3 | **M**CS | **3.68x10^-04^** |
| R4 | 169-312 | 21cn-R-CN-D1  62cn-N-R-CN-D2  63cn-R-CN-D3 | Q281-R-WS-1989-C1  TOS93-R-TO-2010-C1 | Unknown | **R**GB | **2.95x10^-03^** |
| R6 | 97-579 | 6us-R-US^#^  527-R-US-1992-C1^#^  KP9-R-US-1990-C1^#^  Q279-R-WS-1989-C1^#^  Q281-R-WS-1989-C1 | TOS91-R-TO-2010-C1 | 33in-R-IN-2002-C2 | MC**S** | **3.70x10^-03^** |

RDP (R) GENCONV (G), BOOTSCAN (B), MAXCHI (M), CHIMERA (C), SISCAN (S) and 3SEQ (T)

Minor Parent = Parent contributing the smaller fraction of sequence.

Major Parent = Parent contributing the larger fraction of sequence.

Unknown = Only one parent and a recombinant need be in the alignment for a recombination event to be detectable. The sequence listed as unknown was used to infer the existence of a missing parental sequence.

# = Trace evidence was identified for this sequence
