# Supplementary material for: The global distribution of Banana bunchy top virus reveals little evidence for frequent recent, human-mediated long distance dispersal events
Source: Virus Evol. 2015 Sep 10;1(1):vev009. doi: 10.1093/ve/vev009 (PMC5014477; doi:10.1093/ve/vev009)
Supplement: Supplementary Table S1 [file Supp_Table_4.docx]

Supplementary Table 4

| **Recombination Event Number** | **Breakpoints in Alignment** | **Recombinant Sequence(s)** | **Sequence(s) used to infer minor parent(s)** | **Sequence(s) used to infer major parent(s)** | **Detection Methods** | **p-value** |
| --- | --- | --- | --- | --- | --- | --- |
| U2 | 1558-1778 | 27in-U3-IN-2006  48pk-U3-PK-2007^#^  49pk-U3-PK-2007^#^  547-U3-BI-1995^#^  60pk-U3-PK  64in-U3-IN-2009^#^  AY884172-U3-IN^#^  AY996563-U3-PK-2007^#^  TOS4-U3-TO-2010  TOS58-U3-TO-2010  TOS59-U3-TO-2010  TOS61-U3-TO-2010  TOS62-U3-TO-2010  TOS69-U3-TO-2010  TOS71-U3-TO-2010  TOS74-U3-TO-2010  35to-U3-TO-2010-C1  36to-U3-TO-2010-C1  38to-U3-TO-2010-C1  TOS56-U3-TO-2010-C1  TOS83-U3-TO-2010-C1  523-6B-U3-IN-1991-D8^#^  All C2 except 7  *3in-U3-IN-2007-C2*  *33in-U3-IN-2002-C2*  *51in-U3-IN-C2*  *65lk-U3-LK-2010-C2*  *Q524-1-U3-IN-C2*  *Q524-3-U3-IN-C2* | Unknown | 22in-U3-IN  24tw-U3-TW  28in-U3-IN-2012  34eg-U3-EG-1997  6us-U3-US  602-U3-AU-1996  9-150510-U3-EG-2010  AY884173-U3-IN  B2818-U3-AU-2011  B2823-U3-AU-2011  B2827-U3-AU-2011  B2828-U3-AU-2011  B2830-U3-AU-2011  B2833-U3-AU-2011  B2834-U3-AU-2011  EU046323-U3-IN  Q524-2-U3-IN  Q529-6-U3-CN-1990  TOS14-U3-TO-2010  TOS19-U3-TO-2010  TOS40-U3-TO-2010  TOS43-U3-TO-2010  TOS49-U3-TO-2010  TOS55-U3-TO-2010  TOS63B-U3-TO-2010  TOS64-U3-TO-2010  TOS67-U3-TO-2010  TOS68-U3-TO-2010  TOS72-U3-TO-2010  TOS78-U3-TO-2010  TOS79-U3-TO-2010  TOS80-U3-TO-2010  TOS82-U3-TO-2010  TOS85-U3-TO-2010  TOS87-U3-TO-2010  TOS88-U3-TO-2010  TOS89-U3-TO-2010  All A1 1/1  3in-U3-IN-2007-C2  33in-U3-IN-2002-C2  51in-U3-IN-C2  65lk-U3-LK-2010-C2  Q524-1-U3-IN-C2  Q524-3-U3-IN-C2  All C3 22/22  627-U3-TW-1996-D8  All C1 except 6  *35to-U3-TO-2010-C1*  *36to-U3-TO-2010-C1*  *38to-U3-TO-2010-C1*  *44to-U3-TO-2010-C1*  *TOS56-U3-TO-2010-C1*  *TOS83-U3-TO-2010-C1* | R**G**MCST | **1.17x10^-17^** |
| U4 | 1674-17 | 25tw-U3-TW-D5  MS14-U3-PH-2008 | Unknown | 625-U3-TW-1996  AY996563-U3-PK-2007  DQ826392-U3-TW  GU559703-U3-CN-2008  Q568-3-U3-ID-1995  BU10-U3-CD-2012-C2  BU17-U3-CD-2012-C2  All D2 1/1  All D4 1/1  All D7 1/1  All D5 except 2  *571-2-U3-PH-1993-D5*  *25-TW-U3-TW-D5 (recombinant)* | R**G**MST | 7.77x10^-12^ |
| U5 | 1706-214 | AY996563-U3-PK-2007  19rw-U3-RW-2009-C2^#^  20rw-U3-RW-2009-C2^#^  BU1-U3-CD-2012-C2^#^  BU10-U3-CD-2012-C2  BU17-U3-CD-2012-C2  BU9-U3-CD-2012-C2^#^ | 625-U3-TW-1996  All D2 1/1  522A-U3-PH-1991-D5  522B-U3-PH-1991-D5  Q1160-U3-TW-1995-D5  Q568-1-U3-ID-1995-D5 | 24tw-U3-TW  34eg-U3-EG-1997  6us-U3-US  602-U3-AU-1996  9-150510-U3-EG-2010  AY884173-U3-IN  B2818-U3-AU-2011  B2820-U3-AU-2011  B2823-U3-AU-2011  B2827-U3-AU-2011  B2828-U3-AU-2011  B2830-U3-AU-2011  EU046323-U3-IN  Q524-2-U3-IN  Q529-2-U3-CN-1990  Q529-6-U3-CN-1990  TOS14-U3-TO-2010  TOS40-U3-TO-2010  TOS43-U3-TO-2010  TOS49-U3-TO-2010  TOS55-U3-TO-2010  TOS63B-U3-TO-2010  TOS64-U3-TO-2010  TOS67-U3-TO-2010  TOS68-U3-TO-2010  TOS72-U3-TO-2010  TOS78-U3-TO-2010  TOS79-U3-TO-2010  TOS80-U3-TO-2010  TOS82-U3-TO-2010  TOS85-U3-TO-2010  TOS87-U3-TO-2010  TOS89-U3-TO-2010  All A1 1/1  3in-U3-IN-2007-C2  33in-U3-IN-2002-C2  51in-U3-IN-C2  65lk-U3-LK-2010-C2  Q524-1-U3-IN-C2  Q524-3-U3-IN-C2  627-U3-TW-1996-D8  All E1 1/1  All C1 except 9  *35to-U3-TO-2010-C1*  *36to-U3-TO-2010-C1*  *38to-U3-TO-2010-C1*  *TOS25-U3-TO-2010-C1*  *TOS29-U3-TO-2010-C1*  *TOS42-U3-TO-2010-C1*  *TOS56-U3-TO-2010-C1*  *TOS83-U3-TO-2010-C1*  *TOS90-U3-TO-2010-C1*  All C3 except 2  *482-98-U3-AU-1998-C3*  *KP18-U3-AU-2010-C3* | R**G**T | 4.07x10^-08^ |
| U6 | 1321-1789 | TOS93-U3-TO-2010-C1 | Unknown | Q529-6-U3-CN-1990  TOS55-U3-TO-2010  TOS80-U3-TO-2010  TOS65-U3-TO-2010-C1 | B**M**T | **1.22x10^-04^** |
| U7 | 1585-353 | 5tw-U3-TW | Unknown | 625-U3-TW-1996  625I-U3-TW-1995  DQ826392-U3-TW  FJ773283-U3-TW  GU559702-U3-CN-2009  GU559703-U3-CN-2008  GU559704-U3-CN-2009  GU559705-U3-CN-2009  GU559706-U3-CN-2009  HM212635-U3-CN-2009  MP1-U3-TW-1996  MS14-U3-PH-2008  Q529-2-U3-CN-1990  Q568-3-U3-ID-1995  All D1 1/1  All D2 1/1  All D3 1/1  All D4 1/1  All D5 16/16  All D6 1/1  All D7 1/1  All E1 1/1 | GMS**T** | **5.93x10^-19^** |
| U8 | 1608-38 | 625-U3-TW-1996  625I-U3-TW-1995^#^  DQ826392-U3-TW  FJ773283-U3-TW^#^  GU559703-U3-CN-2008  MP1-U3-TW-1996  MS14-U3-PH-2008  Q568-3-U3-ID-1995  All D2 1/1  All D4 1/1  All D5 16/16  All D6 1/1  All D7 1/1 | Unknown | GU559702-U3-CN-2009  GU559704-U3-CN-2009  GU559705-U3-CN-2009  GU559706-U3-CN-2009  HM212635-U3-CN-2009  All D1 1/1  All D3 1/1 | **R**GMCST | **7.39x10^-11^** |
| U10 | 356*-567 | 5tw-U3-TW | TOS19-U3-TO-2010  TOS58-U3-TO-2010  TOS64-U3-TO-2010  All A1 1/1  38to-U3-TO-2010-C1  43to-U3-TO-2010-C1  527-U3-US-1992-C1  536-U3-TO-1993-C1  KP4-U3-TO-1990-C1  Q279-U3-WS-1989-C1  Q570-U3-TO-1990-C1  TOS16-U3-TO-2010-C1  TOS39-U3-TO-2010-C1  TOS42-U3-TO-2010-C1  TOS65-U3-TO-2010-C1  TOS90-U3-TO-2010-C1 | 571-1-U3-PH-1993-D5 | **R**GBST | **7.21x10^-05^** |
| U12 | 374-534 | Q529-4-U3-CN-1990-E1  Q529-2-U3-CN-1990^#^ | TOS64-U3-TO-2010  TOS39-U3-TO-2010-C1  43to-U3-TO-2010-C1 | Unknown | R**G**B | **1.86x10^-03^** |
| U17 | 460-1011 | AY884173-U3-IN  Q529-6-U3-CN-1990  33in-U3-IN-2002-C2  51in-U3-IN-C2  65lk-U3-LK-2010-C2  Q524-1-U3-IN-C2  Q524-3-U3-IN-C2^#^ | Unknown | 8-150510-U3-EG-2010-A1 | **M**CST | **6.68x10^-05^** |
| U19 | 357-563 | 66in-U3-IN-2012-B1 | 21cn-U3-CN-D1 | 482-98-U3-AU-1998-C3 | RMC**S** | **1.07x10^-04^** |
| U21 | 1010-1558 | 8-150510-U3-EG-2010-A1 | Unknown | B2818-U3-AU-2011  B2820-U3-AU-2011  B2823-U3-AU-2011  B2827-U3-AU-2011  24-TW-U3-TW  602-U3-AU-1996  B2828-U3-AU-2011  B2830-U3-AU-2011  B2833-U3-AU-2011  B2834-U3-AU-2011  44to-U3-TO-2010-C1  45to-U3-TO-2010-C1  46to-U3-TO-2010-C1  All C3 except 2  *KP14-U3-AU-2009-C3*  *KP17-U3-AU-2010-C3* | MC**S** | **1.85x10^-05^** |
| U22 | 371-504 | TOS43-U3-TO-2010 | Unknown | TOS42-U3-TO-2010-C1 | R**G**B | **2.47x10^-03^** |

RDP (R) GENCONV (G), BOOTSCAN (B), MAXCHI (M), CHIMERA (C), SISCAN (S) and 3SEQ (T)

Minor Parent = Parent contributing the smaller fraction of sequence.

Major Parent = Parent contributing the larger fraction of sequence.

Unknown = Only one parent and a recombinant need be in the alignment for a recombination event to be detectable. The sequence listed as unknown was used to infer the existence of a missing parental sequence.

# = Trace evidence was identified for this sequence
