# Supplementary material for: The global distribution of Banana bunchy top virus reveals little evidence for frequent recent, human-mediated long distance dispersal events
Source: Virus Evol. 2015 Sep 10;1(1):vev009. doi: 10.1093/ve/vev009 (PMC5014477; doi:10.1093/ve/vev009)
Supplement: Supplementary Table S1 [file Supp_Table_5.docx]

Supplementary Table 5

| **Recombination Event Number** | **Breakpoints in Alignment** | **Recombinant Sequence(s)** | **Sequence(s) used to infer minor parent(s)** | **Sequence(s) used to infer major parent(s)** | **Detection Methods** | **p-value** |
| --- | --- | --- | --- | --- | --- | --- |
| S1 | 1193-68 | 626-S-TW-1996  626M-S-TW-1995  MP2-S-TW-1996-D6 | Unknown | 10ph-S-PH  13ph -S-PH  14ph -S-PH  15-ph -S-PH  16id-S-ID  17id -S-ID  18id -S-ID  7jp-S-JP  768-S-PH-1995  8jp-S-JP  9jp-S-JP  AF148068-S-PH  AF148942-S-TW  AF238877-S-CN  MP1-S-TW-1996  MS14-S-PH-2008  All D2 1/1  All D3 1/1  All D4 1/1  All D5 16/16  All D8 2/2 | RG**T** | **3.96x10^-09^** |
| S2 | 362-730 | 10ph-S-PH  11vn-S-VN^#^  12vn-S-VN^#^  13ph-S-PH  14ph-S-PH  15ph-S-PH  16id-S-ID  17id-S-ID  18id-S-ID  5tw-S-TW  625-S-TW-1996  625I-S-TW-1995  626-S-TW-1996  626M-S-TW-1995  7jp-S-JP  768-S-PH-1995  8jp-S-JP  9jp-S-JP  AF148068-S-PH  AF148942-S-TW  AF148945-S-VN^#^  AF238876-S-CN  AF238877-S-CN^#^  MP1-S-TW-1996  MS14-S-PH-2008  Q529-1-S-CN-1990  Q529-2-S-CN-1990^#^  Q529-5-S-CN-1990^#^  All D1 1/1  All D2 1/1  All D3 1/1  All D4 1/1  All D5 16/16  All D6 1/1  All D8 2/2  All E1 1/1 | B2846-S-AU-2011  BU18-S-CD-2012-C2  BU9-S-CD-2012-C2 | Unknown | RGMCS**T** | **3.72x10^-06^** |
| S5 | 421-478 | B2846-S-AU-2011 | Unknown | 1pk-S-PK-2004  2pk-S-PK-2004  AM418565-S-PK-2004  AM418567-S-PK-2004  GQ249344-S-CM-2008  JF755978-S-CM-2008  JF755980-S-MW-2008  JF755981-S-GA-2008  JF755982-S-GA-2008  JF755984-S-CD-2008  JF755986-S-CD-2008  JF755987-S-CD-2008 | RG**B** | **6.90x10^-04^** |
| S7 | 392*-514 | JF755981-S-GA-2008  JF755984-S-CD-2008 | Unknown | B2819-S-AU-2011-C3 | RG**B** | **2.90x10^-03^** |
| S8 | 1193-64 | 5tw-S-TW^#^  625-S-TW-1996^#^  625I-S-TW-1995 | Unknown | 571-2-S-PH-1993-D5 | R**G**B | **7.27x10^-03^** |

RDP (R) GENCONV (G), BOOTSCAN (B), MAXCHI (M), CHIMERA (C), SISCAN (S) and 3SEQ (T)

Minor Parent = Parent contributing the smaller fraction of sequence.

Major Parent = Parent contributing the larger fraction of sequence.

Unknown = Only one parent and a recombinant need be in the alignment for a recombination event to be detectable. The sequence listed as unknown was used to infer the existence of a missing parental sequence.

# = Trace evidence was identified for this sequence
