# Supplementary material for: The global distribution of Banana bunchy top virus reveals little evidence for frequent recent, human-mediated long distance dispersal events
Source: Virus Evol. 2015 Sep 10;1(1):vev009. doi: 10.1093/ve/vev009 (PMC5014477; doi:10.1093/ve/vev009)
Supplement: Supplementary Table S1 [file Supp_Table_6.docx]

Supplementary Table 6

| **Recombination Event Number** | **Breakpoints in Alignment** | **Recombinant Sequence(s)** | **Sequence(s) used to infer minor parent(s)** | **Sequence(s) used to infer major parent(s)** | **Detection Methods** | **p-value** |
| --- | --- | --- | --- | --- | --- | --- |
| M1 | 406-759 | 66in-M-IN-2012-B1 | 1429A-M-AU  547-M-BI-1995  33in-M-IN-2002-C2  548-M-BI-1995-C2  BU12-M-CD-2012-C2  BU13-M-CD-2012-C2  BU17-M-CD-2012-C2 | TOS72-M-TO-2010  TOS15-M-TO-2010  TOS19-M-TO-2010  TOS43-M-TO-2010  TOS55-M-TO-2010  TOS57-M-TO-2010  TOS59-M-TO-2010  TOS63B-M-TO-2010  TOS67-M-TO-2010  TOS68-M-TO-2010  TOS69-M-TO-2010  TOS71-M-TO-2010  TOS76-M-TO-2010  TOS78-M-TO-2010  TOS80-M-TO-2010  TOS82-M-TO-2010  TOS85-M-TO-2010  TOS87-M-TO-2010  TOS89-M-TO-2010  B2828-M-AU-2011  B2830-M-AU-2011  B2833-M-AU-2011  B2834-M-AU-2011  B2846-M-AU-2011  35to-M-TO-2010-C1  37to-M-TO-2010-C1  40to-M-TO-2010-C1  41to-M-TO-2010-C1  536-M-TO-1993-C1  KP4-M-TO-1990-C1  Q276-M-TO-1989-C1  Q277-M-TO-1989-C1  Q570-M-TO-1990-C1  TOS16-M-TO-2010-C1  TOS29-M-TO-2010-C1  TOS56-M-TO-2010-C1  TOS63A-M-TO-2010-C1  TOS65-M-TO-2010-C1  TOS83-M-TO-2010-C1  TOS91-M-TO-2010-C1  736-4-M-IN-1997-C2  1900B-M-AU-2006-C3  2557-M-AU-2010-C3  482-98-M-AU-1998-C3  B2845-M-AU-2011-C3  KP14-M-AU-2009-C3  KP17-M-AU-2010-C3 | GBM**S** | **9.97x10^-05^** |
| M5 | 1285-15 | ABTV3-M-MY | 1pk-M-PK-2004  1429B-M-AU  22in-M-IN  24tw-M-TW  523-6A-M-IN-1991  768-M-PH-1995  AF349568-M-CN  AY953429-M-IN  HE864318-M-PK  HE864319-M-PK  KC581796-M-TH-2012  KP7-M-AU-1989  KP8-M-AU-1989  MS14-M-PH-2008  Q529-6-M-CN-1990  Q568-3-M-ID-1995  TOS40-M-TO-2010  TOS49-M-TO-2010  TOS88-M-TO-2010  42to-M-TO-2010-C1  43to-M-TO-2010-C1  45to-M-TO-2010-C1  527-M-US-1992-C1  KP9-M-US-1990-C1  Q281-M-WS-1989-C1  TOS39-M-TO-2010-C1  TOS48-M-TO-2010-C1  All D1 1/1  All D2 1/1  All D3 1/1  All D4 1/1  All D8 2/2  All E1 1/1  All D5 except 5  *25tw-M-TW-D5*  *765-M-TW-1996-D5*  *S7-M-PH-2008-D5*  *Q1160-M-TW-1995-D5*  *Q624-M-TW-1996-D5*  All C2 except 8  *33in-M-IN-2002-C2*  *51in-M-IN-C2*  *549-M-BI-1995-C2*  *736-4-M-IN-1997-C2*  *BU14-M-CD-2012-C2*  *BU15-M-CD-2012-C2*  *BU16-M-CD-2012-C2*  *BU20-M-CD-2012-C2* | ABTV2-M-PH | GB**S** | **2.19x10^-04^** |

RDP (R) GENCONV (G), BOOTSCAN (B), MAXCHI (M), CHIMERA (C), SISCAN (S) and 3SEQ (T)

Minor Parent = Parent contributing the smaller fraction of sequence.

Major Parent = Parent contributing the larger fraction of sequence.

Unknown = Only one parent and a recombinant need be in the alignment for a recombination event to be detectable. The sequence listed as unknown was used to infer the existence of a missing parental sequence.

# = Trace evidence was identified for this sequence
