# Supplementary material for: The global distribution of Banana bunchy top virus reveals little evidence for frequent recent, human-mediated long distance dispersal events
Source: Virus Evol. 2015 Sep 10;1(1):vev009. doi: 10.1093/ve/vev009 (PMC5014477; doi:10.1093/ve/vev009)
Supplement: Supplementary Table S1 [file Supp_Table_8.docx]

Supplementary Table 8

| **Recombination Event Number** | **Breakpoints in Alignment** | **Recombinant Sequence(s)** | **Sequence(s) used to infer minor parent(s)** | **Sequence(s) used to infer major parent(s)** | **Detection Methods** | **p-value** |
| --- | --- | --- | --- | --- | --- | --- |
| N1 | 300-318 | TOS53-N-TO-2010 | Unknown | 1pk-N-PK-2004  1429A-N-AU  1429B-N-AU  22in-N-IN  547-N-BI-1995  602-N-AU-1996  625I-N-TW-1995  626-N-TW-1996  64in-N-IN-2009  737-N-AU-1997  AF238878-N-CN  AF238879-N-CN  B2818-N-AU-2011  B2823-N-AU-2011  B2826-N-AU-2011  B2830-N-AU-2011  B2833-N-AU-2011  B2834-N-AU-2011  B2844-N-AU-2011  B2846-N-AU-2011  EF470243-N-CN  HE864320-N-PK  KP8-N-AU-1989  Q529-1-N-CN-1990  Q529-2-N-CN-1990  All A1 1/1  All C1 40/40  All C2 33/33  All D1 1/1  All D2 1/1  All D3 1/1  All D4 1/1  All D6 1/1  All D8 2/2  All C3 except 1  *B2819-N-AU-2011-C3*  All ungrouped TO except 1  *TOS53-N-TO-2010 (recombinant)*  All D5 except 5  *522B-N-PH-1991-D5*  *571-2-N-PH-1993-D5*  *MS7-N-PH-2008-D5*  *Q568-1-N-ID-1995-D5*  *Q624-N-TW-1996-D5* | RG**B** | **2.95x10^-10^** |
| N2 | 732-1091 | TOS40-N-TO-2010  TOS49-N-TO-2010  TOS58-N-TO-2010  36to-N-TO-2010-C1  37to-N-TO-2010-C1  41to-N-TO-2010-C1  42to-N-TO-2010-C1  45to-N-TO-2010-C1  46to-N-TO-2010-C1  TOS39-N-TO-2010-C1  TOS48-N-TO-2010-C1  TOS90-N-TO-2010-C1 | 1pk-N-PK-2004  1429A-N-AU  1429B-N-AU  602-N-AU-1996  737-N-AU-1997  B2818-N-AU-2011  B2823-N-AU-2011  B2826-N-AU-2011  B2830-N-AU-2011  B2833-N-AU-2011  B2834-N-AU-2011  B2844-N-AU-2011  B2846-N-AU-2011  HE864320-N-PK  KP8-N-AU-1989  All A1 1/1  19rw-N-RW-2009-C2  20rw-N-RW-2009-C2  26pk-N-PK-2004-C2  3in-N-IN-2007-C2  BU9-N-CD-2012-C2  Q553-N-LK-1995-C2  All C3 22/22 | TOS14-N-TO-2010  TOS19-N-TO-2010  TOS4-N-TO-2010  TOS5-N-TO-2010  TOS53-N-TO-2010  TOS55-N-TO-2010  TOS57-N-TO-2010  TOS61-N-TO-2010  TOS63B-N-TO-2010  TOS64-N-TO-2010  TOS67-N-TO-2010  TOS68-N-TO-2010  TOS7-N-TO-2010  TOS71-N-TO-2010  TOS74-N-TO-2010  TOS78-N-TO-2010  TOS80-N-TO-2010  TOS82-N-TO-2010  TOS85-N-TO-2010  TOS86-N-TO-2010  TOS87-N-TO-2010  TOS88-N-TO-2010  TOS89-N-TO-2010  TOS93-N-TO-2010-C1  35to-N-TO-2010-C1  38to-N-TO-2010-C1  39to-N-TO-2010-C1  40to-N-TO-2010-C1  43to-N-TO-2010-C1  536-N-TO-1993-C1  KP4-N-TO-1990-C1  Q276-N-TO-1989-C1  Q277-N-TO-1989-C1  Q278-N-TO-1989-C1  Q570-N-TO-1990-C1  TOS2-N-TO-2010-C1  TOS20-N-TO-2010-C1  TOS21-N-TO-2010-C1  TOS22-N-TO-2010-C1  TOS25-N-TO-2010-C1  TOS29-N-TO-2010-C1  TOS42-N-TO-2010-C1  TOS56-N-TO-2010-C1  TOS60-N-TO-2010-C1  TOS63A-N-TO-2010-C1  TOS65-N-TO-2010-C1  TOS91-N-TO-2010-C1 | RGMCS**T** | **2.26x10^-08^** |
| N3 | 804-1229 | ABTV2-N-PH | Unknown | ABTV1-N-MY | RM**S**T | **5.26x10^-22^** |
| N4 | 1167-1198 | BU6-N-CD-2012-C2 | ABTV1-N-MY | 1pk-N-PK-2004  1429A-N-AU  1429B-N-AU  22in-N-IN  547-N-BI-1995  602-N-AU-1996  64in-N-IN-2009  737-N-AU-1997  B2818-N-AU-2011  B2823-N-AU-2011  B2826-N-AU-2011  B2830-N-AU-2011  B2833-N-AU-2011  B2834-N-AU-2011  B2844-N-AU-2011  B2846-N-AU-2011  HE864320-N-PK  KP8-N-AU-1989  TOS4-N-TO-2010  TOS74-N-TO-2010  TOS78-N-TO-2010  TOS82-N-TO-2010  TOS88-N-TO-2010  All A1 1/1  4to-N-TO-2010-C1  45to-N-TO-2010-C1  46to-N-TO-2010-C1  TOS39-N-TO-2010-C1  TOS42-N-TO-2010-C1  TOS83-N-TO-2010-C1  TOS93-N-TO-2010-C1  All C2 except 3  *BU13-N-CD-2012-C2*  *KP5-N-LK-2003-C2*  *BU6-N-CD-2012-C2 (recombinant)*  All C3 except 3  *482-96-N-AU-1996-C3*  *482-97-N-AU-1997-C3*  *482-98-N-AU-1998-C3* | R**G**B | **7.42x10^-06^** |
| N6 | 1172-16 | MP1-N-TW-1996  MP2-N-TW-1996-D6^#^ | Unknown | Q280-N-WS-1989  768-N-PH-1995  Q568-3-N-ID-1995  625-N-TW-1996  626-N-TW-1996  523-6B-N-IN-1991-D8  All D5 except 1  *25tw-N-TW-D5* | R**G**B | **1.03x10^-03^** |
| N7 | 1179-1202 | TOS16-N-TO-2010-C1  TOS22-N-TO-2010-C1  TOS56-N-TO-2010-C1  TOS61-N-TO-2010  BU13-N-CD-2012-C2^#^ | ABTV2-N-PH | TOS93-N-TO-2010-C1 | R**G**B | **7.28x10^-03^** |

RDP (R) GENCONV (G), BOOTSCAN (B), MAXCHI (M), CHIMERA (C), SISCAN (S) and 3SEQ (T)

Minor Parent = Parent contributing the smaller fraction of sequence.

Major Parent = Parent contributing the larger fraction of sequence.

Unknown = Only one parent and a recombinant need be in the alignment for a recombination event to be detectable. The sequence listed as unknown was used to infer the existence of a missing parental sequence.

# = Trace evidence was identified for this sequence
