# Supplementary material for: The global distribution of Banana bunchy top virus reveals little evidence for frequent recent, human-mediated long distance dispersal events
Source: Virus Evol. 2015 Sep 10;1(1):vev009. doi: 10.1093/ve/vev009 (PMC5014477; doi:10.1093/ve/vev009)
Supplement: Supplementary Table S1 [file Supp_Table_7.docx]

Supplementary Table 7

| **Recombination Event Number** | **Breakpoints in Alignment** | **Recombinant Sequence(s)** | **Sequence(s) used to infer minor parent(s)** | **Sequence(s) used to infer major parent(s)** | **Detection Methods** | **p-value** |
| --- | --- | --- | --- | --- | --- | --- |
| C1 | 277-780 | 3in-C-IN-2007-C2 | 66in-C-IN-2012-B1 | 1pk-C-PK-2004  1429A-C-AU  1429B-C-AU  2pk-C-PK-2004  24tw-C-TW  547-C-BI-1995  6us-C-US  602-C-AU-1996  737-C-AU-1997  9-150510-C-EG-2010  B2818-C-AU-2011  B2820-C-AU-2011  B2823-C-AU-2011  B2827-C-AU-2011  B2828-C-AU-2011  B2830-C-AU-2011  B2832-C-AU-2011  B2833-C-AU-2011  B2844-C-AU-2011  B2846-C-AU-2011  KP7-C-AU-1989  KP8-C-AU-1989  Q524-2-C-IN  TOS14-C-TO-2010  TOS15-C-TO-2010  TOS4-C-TO-2010  TOS40-C-TO-2010  TOS43-C-TO-2010  TOS49-C-TO-2010  TOS55-C-TO-2010  TOS57-C-TO-2010  TOS58-C-TO-2010  TOS59-C-TO-2010  TOS61-C-TO-2010  TOS62-C-TO-2010  TOS63B-C-TO-2010  TOS64-C-TO-2010  TOS67-C-TO-2010  TOS68-C-TO-2010  TOS69-C-TO-2010  TOS70-C-TO-2010  TOS71-C-TO-2010  TOS72-C-TO-2010  TOS76-C-TO-2010  TOS78-C-TO-2010  TOS82-C-TO-2010  TOS87-C-TO-2010  All C3 22/22  All C1 except 1 *TOS93-C-TO-2010-C1*  All C2 except 2  *3in-C-IN-2007-C2*  *BU10-C-CD-2012-C2* | RGMC**T** | **1.03x10^-07^** |
| C2 | 1068-28 | 8-150510-C-EG-2010-A1  625-C-TW-1996  765-C-TW-1996-D5  Q624-C-TW-1996-D5  All D6 1/1  All D7 1/1 | Unknown | AY264347-C-CN  AY266417-C-CN  Q568-3-C-ID-1995  MS17-C-PH-2008-D5  Q1160-C-TW-1995-D5  Q568-1-C-ID-1995-D5  523-6B-C-IN-1991-D8  All D3 1/1 | **R**GT | **1.41x10^-06^** |
| C3 | 1108-500 | Q529-2-C-CN-1990  Q529-4-C-CN-1990-E1 | Unknown | 21cn-C-CN-D1 | MC**S** | **4.42x10^-06^** |
| C4 | 430-482* | 526-C-BI-1992-C2 | Unknown | BU18-C-CD-2012-C2 | GB**T** | **6.65x10^-03^** |

RDP (R) GENCONV (G), BOOTSCAN (B), MAXCHI (M), CHIMERA (C), SISCAN (S) and 3SEQ (T)

Minor Parent = Parent contributing the smaller fraction of sequence.

Major Parent = Parent contributing the larger fraction of sequence.

Unknown = Only one parent and a recombinant need be in the alignment for a recombination event to be detectable. The sequence listed as unknown was used to infer the existence of a missing parental sequence.

# = Trace evidence was identified for this sequence
